# Genetic polymorphisms and transcription profiles associated with intracranial aneurysm: a key role for NOTCH3

**DOI:** 10.18632/aging.102111

**Published:** 2019-07-23

**Authors:** Mengqi Li, Xinlong Dong, Shi Chen, Weihan Wang, Chao Yang, Bochuan Li, Degang Liang, Weidong Yang, Xiaozhi Liu, Xinyu Yang

**Affiliations:** 1 Department of Neurosurgery, Tianjin Medical University General Hospital, Tianjin 300052, China; 2 Tianjin Neurological Institute, Key Laboratory of Post-trauma Neuro-repair and Regeneration in Central Nervous System, Ministry of Education, Tianjin Key Laboratory of Injuries, Variations and Regeneration of Nervous System, Tianjin 300052, China; 3 Department of Neurosurgery, Tianjin Fifth Central Hospital, Tianjin 300450, China; 4 Department of Neurosurgery, Fuzhou Second Hospital Affiliated to Xiamen University, Fuzhou 350007, China; 5 Department of Cardiovascular Surgery, Tianjin Medical University General Hospital, Tianjin 300052, China; 6 Collaborative Innovation Center of Tianjin for Medical Epigenetics and Department of Physiology and Pathophysiology, Tianjin Medical University, Tianjin 300052, China

**Keywords:** NOTCH3, intracranial aneurysm, next-generation sequencing, RNA sequencing, data mining

## Abstract

Intracranial aneurysm (IA) incidence is about 1~2%. However, the specific mechanisms of IA onset and development need further study. Our objective was to discover novel IA-related genes to determine possible etiologies further. We performed next-generation sequencing on nineteen Chinese patients with familial IA and one patient with sporadic IA. We obtained mRNA expression data of 129 samples from Gene Expression Omnibus (GEO) and made statistical computing to discover differentially expressed genes (DEGs). The screened IA-related gene *NOTCH3* was determined by bioinformatic data mining. We verified the IA-related indicators of NOTCH3. Association was found between IA and the *NOTCH3* SNPs rs779314594, rs200504060 and rs2285981. Levels of *NOTCH3* mRNA were lower in IA tissue than in control tissue, but higher in peripheral blood neutrophils from IA patients than in neutrophils from controls. Levels of NOTCH3 protein were lower in IA tissue than in cerebral artery tissue. NOTCH3 also decreased the expression of angiogenesis factors in human umbilical vein endothelial cells. Variation in NOTCH3 and alteration of its expression in cerebral artery or neutrophils may contribute to IA. Our findings also describe a bioinformatic-experimental approach that may prove useful for probing the pathophysiology of other complex diseases.

## INTRODUCTION

Intracranial aneurysm (IA) occurs in 1%–2% of the general population [1]. IA rupture is a major contributing factor of hemorrhagic stroke [2]. Both environmental and genetic factors are related to the onset and development of IA [3, 4]. Smoking, sex and blood pressure can be considered as the most relevant environmental factors for the rupture of IA [5]. A family history of IA is frequently observed, and this is indicated that genetics may be a major risk factor for IA [6]. Despite this, how environmental and genetic factors lead to IA? This problem needs to be further study.

There are some new findings of IA. Hemodynamic factors, such as irregular shear stress and oscillatory shear index, may contribute to the development and rupture of IA [7, 8]. Single-nucleotide polymorphisms (SNPs) related to IA overlap with the regulatory region of genes expressed in the circle of Willis, which is the location with the highest incidence of aneurysms [9]. The circle of Willis shows anomalies more often among patients with ruptured IA than among those with unruptured IA [10]. Inflammatory cells and factors also play a role in IA onset, growth, and rupture [3, 11–13]. Recently, a case-control study demonstrated that IA-associated mRNA expression was elevated in the peripheral blood neutrophil of aneurysm patients [14, 15]. IA has been associated with some inflammatory factors such as IL-1β, IL-6, TNF-α, MMP9, MMP2, NF-κB, MCP-1, and VCAM1 [8, 11–13, 16–20]. However, we do not know exactly which genetic abnormality is responsible for intracranial aneurysms, and this is also what we should study and explore at present.

IA is associated with many hereditary diseases, and many genes have been associated with IA [21]. Build on these latest IA-related findings, here we used next-generation sequencing, transcriptome sequencing, comprehensive data mining, and studies with human umbilical vein endothelial cells (HUVECs) to screen for genes associated with IA. We discovered that NOTCH3 is associated with the disease, first from *in silico* analyses, followed by verification using immunohistochemistry of IA and cerebral artery tissue.

## RESULTS

### Preliminary screening of high-throughput data for IA-related genes

**Figure 1:**
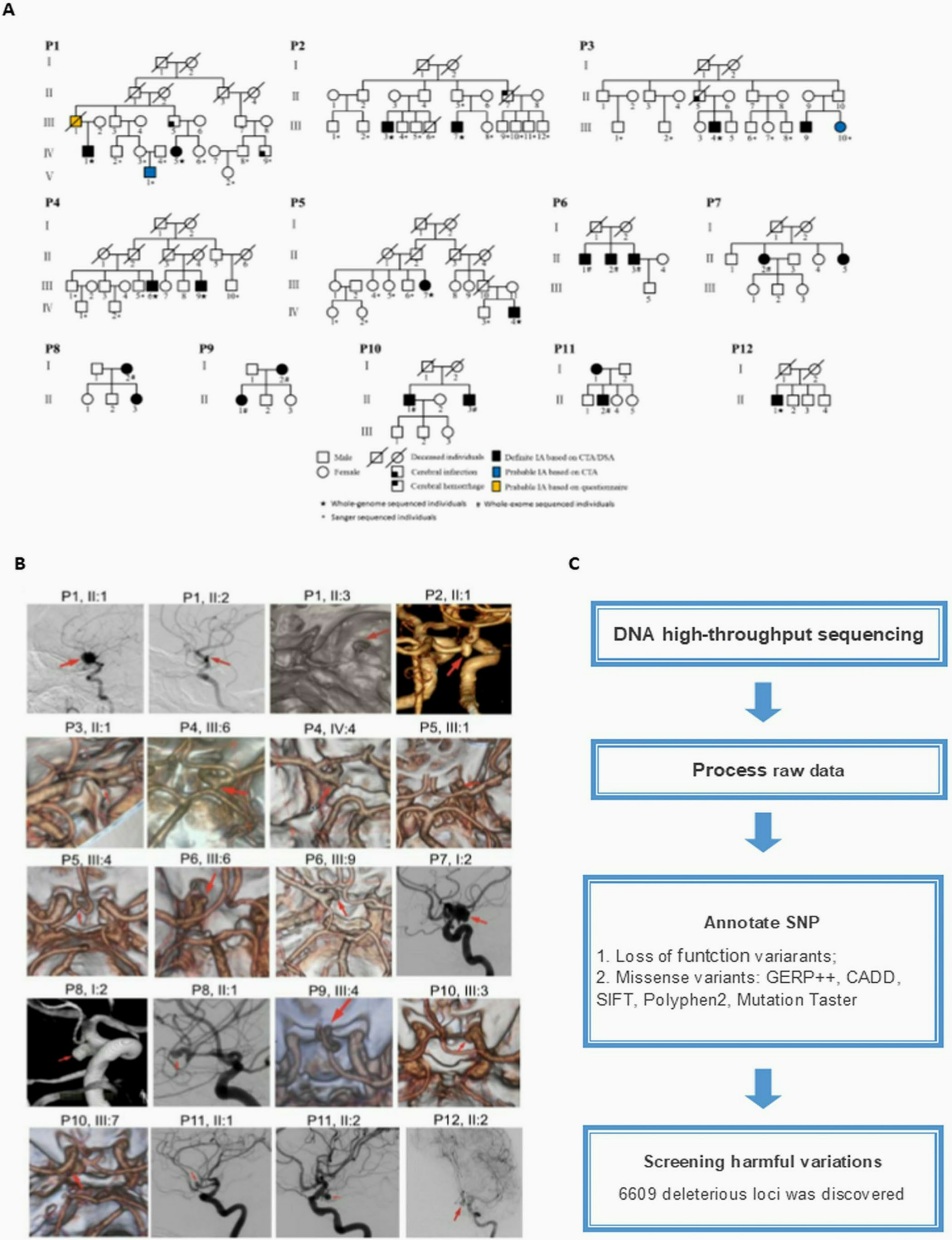
(**A**) Pedigrees of the 20 cases with intracranial aneurysm (IA). (**B**) IA reconstruction from digital subtraction angiography, magnetic resonance angiography, and computed tomography angiography. The red arrows indicate the location of the aneurysm. (**C**) Workflow for filtering deleterious single-nucleotide polymorphisms (SNPs).

**Figure 2:**
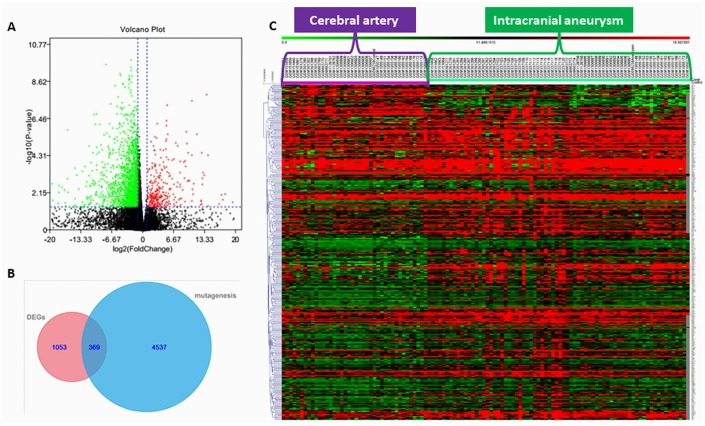
(**A**) Volcano plot of significantly up-regulated (red) and down-regulated (green) DEGs. (**B**) Venn diagram showing the number of genes from next-generation sequencing (NGS) that were considered mutant (mutagenesis), the number of genes from transcriptome sequencing data in the GEO database considered differentially expressed genes (DEGs), and the number of genes common to the NGS and transcriptome sequencing datasets. (**C**) Heat map showing expression of genes overlapping between the NGS and transcriptome sequencing datasets.

We preliminarily screened IA-related genes from high throughput genomic and transcriptome data. In order to screen IA-related mutant genes, we performed high-throughput genome sequencing for 20 Chinese subjects (19 were familial cases, while 1 was a sporadic case) with IA as a discovery cohort (Figure 1A and 1B). A total of 6649 deleterious SNPs in 4906 genes was discovered from profiling our next generation sequencing data, which was annotated and filtered based on functional changes by SAMtools, ANNOVAR, PolyPhen-2 and SIFT software (Figure 1C). Transcriptome sequencing data from GEO database revealed 1422 DEGs, of which 147 were upregulated and 1275 were downregulated in IA compared to healthy cerebral artery (Supplementary Table 1). The screened DEGs are shown in the volcano plot (Figure 2A).

### Determining key IA-related gene(s)

**Figure 3:**
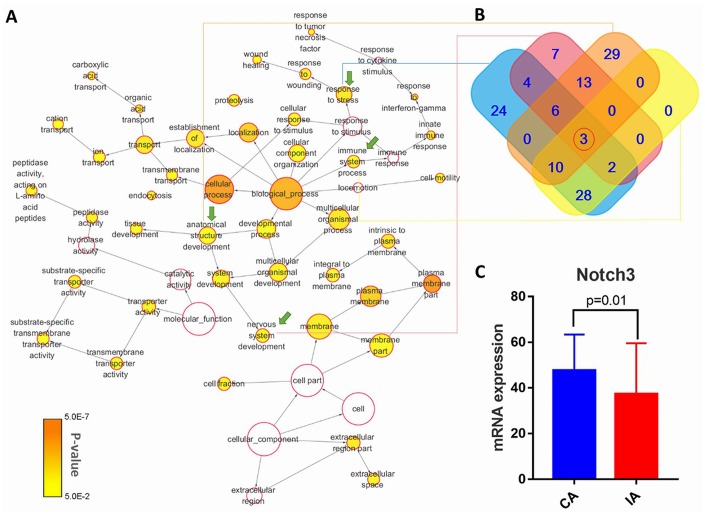
(**A**) Schematic showing enrichment of functions from intersections of filtered mutant genes and DEGs. The color of the node indicates the significance of gene representation, and its size corresponds to the number of genes in that gene ontology (GO) category. The IA-related GO-terms that were carried forward are indicated by green arrows. (**B**) Diagram showing unique and shared genes from the selected IA-related GO terms. (**C**) NOTCH3 mRNA expression data from IA and cerebral artery (CA) tissue, as deposited in the GEO database.

There were 369 genes shared between the mutant genes identified in our next-generation sequencing and the DEGs identified from GEO transcriptome sequencing data (Figure 2B and 2C) [22]. These shared genes were classified based on their functionality according to GO categories tool BiNGO (Figure 3A) [23]. Next, we screened the most relevant GO classifications. As previously published work, IA onset and development can be promoted due to abnormal hemodynamic stress, immune response, or cerebrovascular hypoplasia [7–10, 24]. Considering this, we focused on four GO categories that seemed to be IA-related: response to stress (FDR-adjusted P-value = 6.93×10^-3^), immune system process (FDR-adjusted P-value = 3.57×10^-2^), anatomical structure development (FDR-adjusted P-value = 3.97×10^-2^), and nervous system development (FDR-adjusted P-value = 1.49×10^-2^) (Table 1). From the intersection of the genes of their GO categories, we further narrowed our focus to three genes (TACC3, TRPM2, and NOTCH3) that were shared across all four groups (Figure 3B). However, we found that only NOTCH3 of these screened three genes was associated with cerebral artery disease in previous work [25]. Therefore, we hypothesized that NOTCH3 might be the key IA-related gene. Indeed, mRNA expression data from the GEO database showed NOTCH3 to be significantly downregulated in IA tissue compared to cerebral artery tissue (Figure 3C).

**Table 1:** Genes classified under Gene Ontology (GO) terms related to intracranial aneurysm

**GO-ID and GO-terms**	**p value**		
	**unadjusted**	**False discovery rate-adjusted**	**Genes in intracranial aneurysm-related GO-terms**
6950 response to stress	2.14E-05	6.93E-03	SLC23A2,AVIL,HFE,BRCA1,TRH,CXCL16,CHAF1A,CDH3,DMBT1, CREB3L4,EXO1,ADORA3,KYNU,GTSE1,IKBKE,TNFRSF4,AP3B1, NRG1,AVPR1A,DGKZ,TLR1,ATRN,CCKBR,SMO,RTN4RL1,ADAM9, MASP2,TLR4,SCG2,PPARD,NOTCH3,PNKP,PROZ,AGER,C2,CYP27B1,TRPM2,LMAN1,CLN3,ADAMTS13,APOL1,NGFR,POLQ,GCKR,VDR, F12,XRCC3,HPS1,LY86,XRCC1,USP28,DEF6,SYT7,CRYGD,RAD51, NEDD4,TRPV4,POLE2,TACC3,FOXA3,FOXA2
7399 nervous system development	7.19E-05	1.49E-02	RET, NOTCH3, LAMA1, AVIL, CPNE6,CHRD,SEMA3E,PPP1R9A,AGER,IGSF9,CELSR3,NPAS2, TRPM2,SALL1,SIX4,CHL1,SLIT1,GPC2, NKX6-1, ZNF488,SH3GL1,NGFR,SEMA6A,EDN3,UNC5B,LRRN4,LIMK1,NRG1, SEMA4F,MYO7A,AVPR1A,SYNJ2,SEMA4G,TAL2,SMO,DOK5,RTN4RL1, NEDD4,KCNQ2,TACC3,EPHA2,PPARD,FOXA2
2376 immune system process	4.14E-04	3.57E-02	NOTCH3,FLT3,HFE,STXBP2,CTSW,TREM2,CD1B,CXCL14,RASGRP4, C2,RELB,CXCL16,CYP27B1, TRPM2,DMBT1,SIX4,EXO1,KYNU, APOL1,CD34,IKBKE,TNFRSF4,EDN3,VDR,F12,LY86,AP3B1,TLR1,PC, TACC3,ADAM9,MASP2,TLR4,SCG2,BCAR1
48856 anatomical structure development	4.79E-04	3.97E-02	RET,FLT3,AVIL,TREH,CPNE6,PPP1R9A,CELSR3,TGM1,ADAMTS2, CYP26B1,SALL1,DMBT1,SIX4,CHL1,EXO1,SALL4,TRPS1,COL10A1, BTRC,ZNF488,SH3GL1,FOXD1,SEMA6A,EDN3,UNC5B,NRG1,MYO7A, AVPR1A,COL2A1,SPINT1,TAL2,SMO,ANGPTL6,DOK5,RTN4RL1, KCNQ2,MMP19,ADAM9,SCG2,EPHA2,PPARD,NOTCH3,TMPRSS6, LAMA1,CHRD,SEMA3E,PPL,AGER,IGSF9,NPAS2,RASGRP4,RELB, CYP27B1,TRPM2,CHST11,RAB26,SLIT1,GPC2,NKX61,ARSE, NGFR,CDSN,VDR,LRRN4,LIMK1,PCDH8,SEMA4F,SYNJ2,SEMA4G, ALOX15B,DAB2,PC,NEDD4,ITGA11,TACC3,HOXD4,FOXA2

### Association of IA with *NOTCH3* SNPs

**Figure 4:**
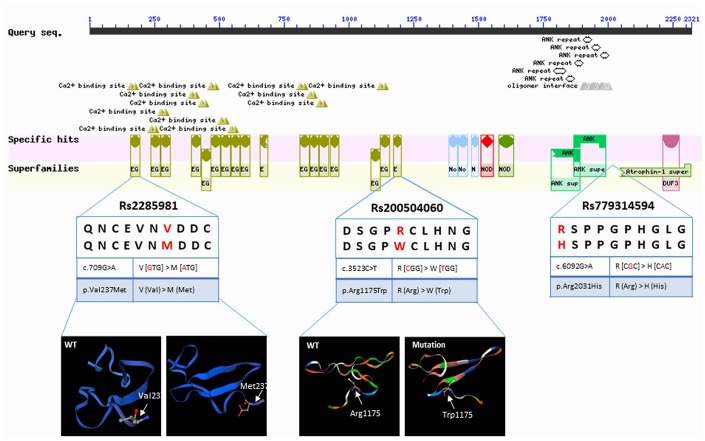
Sequence query of NOTCH3 conserved domains. Reprinted from https://www.ncbi.nlm.nih.gov/Structure/cdd/wrpsb.cgi?INPUT_TYPE=live&SEQUENCE=AAB91371.1. Data show allelic variants that result in amino acid changes and protein structure, as modeled by SWISS-MODEL (https://www.swissmodel.expasy.org/).

Our analysis identified three low-frequency *NOTCH3* SNPs related to IA in our discovery cohort: allele T of rs779314594, allele A of rs200504060, and allele T of rs2285981. Bioinformatic software tools predicted that allele T of rs779314594, allele A of rs200504060, and allele T of rs2285981 lead to loss-of-function. Allele T of rs779314594 and allele A of rs200504060, both in the conserved domain of NOTCH3, were predicted to alter an amino acid residue and therefore potentially the protein structure (Figure 4) [26]. These SNPs was predicted be deleterious by bioinformatics tools GERP++ [47], SIFT [46], CADD [48], PolyPhen-2 [49], and MutationTaster [29] (Table 2). All three risk alleles were enrichment in the IA cases of our discovery cohort compared to the different populations of large genetic variant databases (Genome Aggregation Database, gnomAD) (http://gnomad.broadinstitute.org/) (Table 3) [27]. When we used the gnomAD data as controls, statistical analyses confirmed that IA was associated with allele T of rs779314594 (P = 0.001), allele A of rs200504060 (P = 0.004), and allele T of rs2285981 (P = 0.004). However, no significant enrichment was detected in the sporadic IA cases (Supplementary Table 2).

**Table 2:** Functional annotation of variations at the NOTCH3 locus

**ID**	**REF**	**ALT**	**Func**	**ExonicFunc**	**SIFT^a^**	**MutationTaster^b^**	**gerp++gt2^c^**	**CADD^d^**
rs779314594	C	T	exonic	missense SNV	0.183,T	1.000,D	3.92	16.94
rs200504060	G	A	exonic	missense SNV	0.006,D	1.000,N	2.93	16.86
rs2285981	C	T	exonic	missense SNV	0.002,D	1.000,D	4.41	15.22

ALT, Sample genome base type; REF, Reference genome base type; SNV, single nucleotide variant

^a^ SIFT score indicates whether the variation is likely to cause changes in protein structure or function: “D”, deleterious (sift ≤ 0.05); “T”, tolerated (sift > 0.05);

^b^ MutationTaster represents the effect of the mutation on the protein sequence: “A”, “disease_causing_automatic”; “D”, “disease_causing”; “N”, “polymorphism”; “P”, “polymorphism_automatic”

^c^ Variations with a gerp++gt2 score > 2 are considered conservative.

^d^ CADD score >15 means that the variation affects protein function.

### NOTCH3 expression in IA and healthy cerebral artery

**Figure 5:**
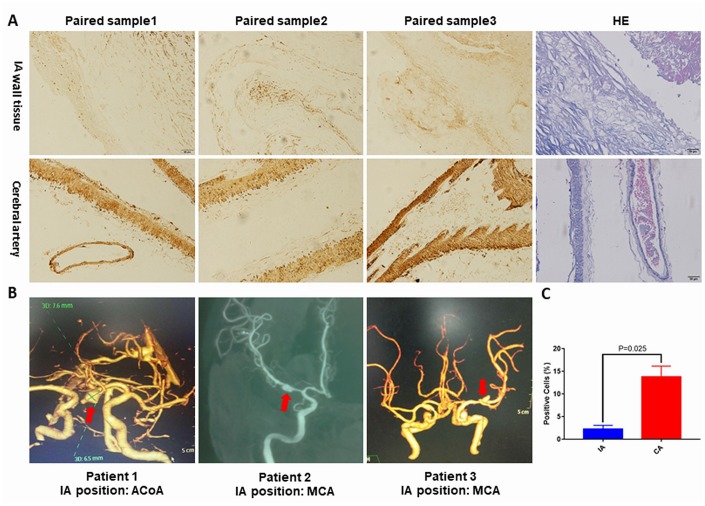
(**A**) Representative pictures of NOTCH3 immunohistochemistry staining or hematoxylin and eosin staining of IA and cerebral artery tissues. (**B**) Reconstructed images from diagnostic CTA and DSA scans. The red arrows indicate the location of the IA. (**C**) Percentage of positively stained cells in panel (A) as measured using Image J. ACoA: anterior communicating artery, MCA: middle cerebral artery. p<0.05.

We tested NOTCH3 expression in the IA and cerebral artery by immunohistochemistry. Staining showed significantly lower NOTCH3 expression in the IA (Figure 5A and 5C). The Imaging diagnosis of IA specimens was provided in Figure 5B. The decreased mRNA and protein expression of NOTCH3 in IA tissue suggests that NOTCH3 can protect against IA. We further confirmed that the genotypes of these samples did not contain risk variants allele T of rs779314594 allele A of rs200504060, and allele T of rs2285981 (Supplementary Table 3).

### NOTCH3 expression in neutrophils

**Figure 6:**
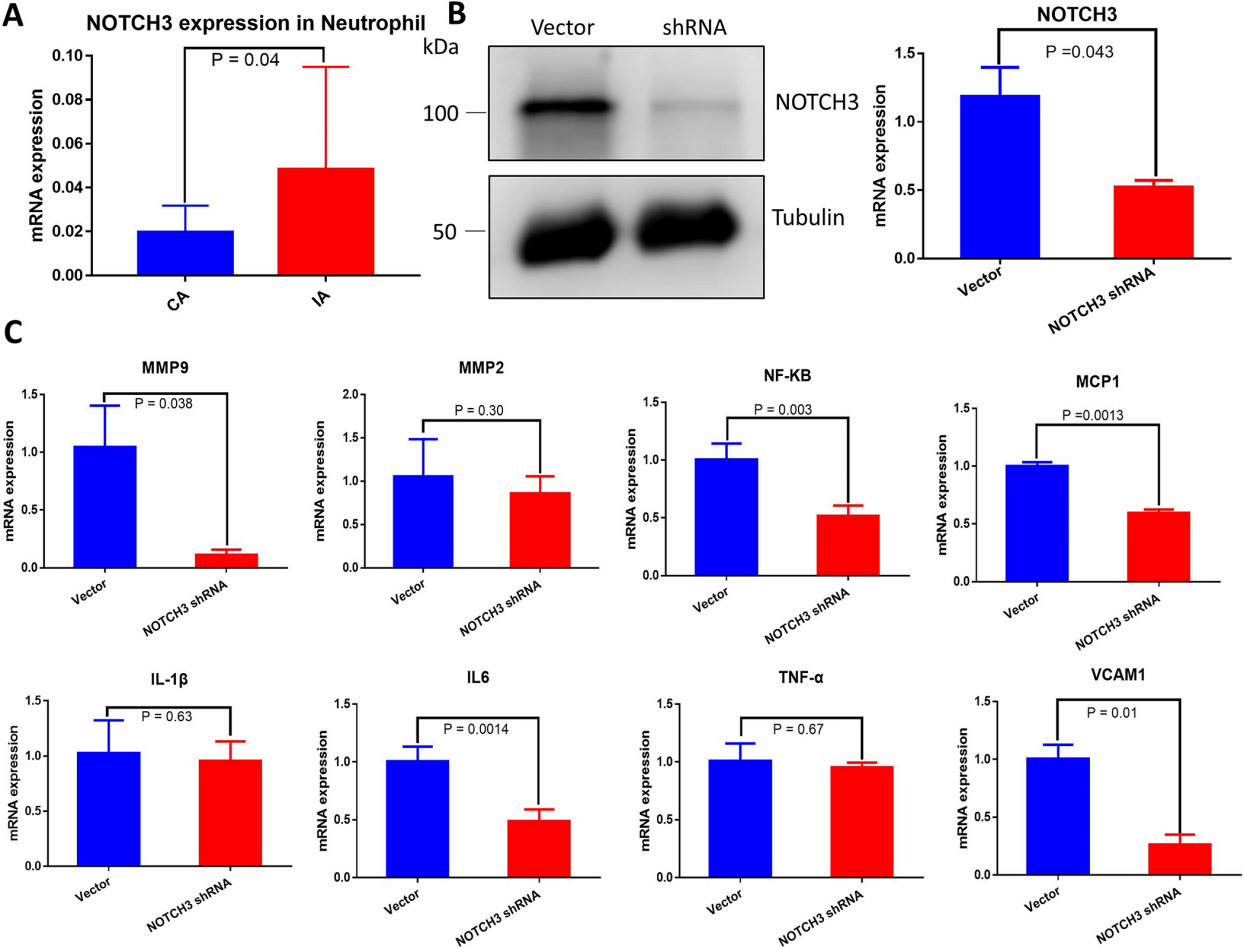
(**A**) Quantification of NOTCH3 transcription in peripheral blood neutrophils from samples with or without IA. (**B**) Western blot and RT-qPCR of whole cell lysate from HUVECs transduced with negative control shRNA or NOTCH3-shRNA. (**C**) Quantification of IA-related factor transcripts from HUVECs transduced with negative control shRNA or NOTCH3-shRNA.

Due to the role of the immune response in IA rupture, we retrieved data on NOTCH3 expression in peripheral blood neutrophils from individuals with IA and healthy controls from the GEO database (https://www.ncbi.nlm.nih.gov/geo, GSE106520) [14]. NOTCH3 mRNA expression was significantly higher in IA neutrophils (Figure 6A).

### NOTCH3 knockdown down-regulates angiogenesis factors in HUVEC

IA has been associated with IL-1β, IL-6, TNF-α, MMP9, MMP2, NF-κB, MCP-1, and VCAM1 [8, 11, 12, 18–20]. To determine whether these factors are regulated by NOTCH3, we transfected HUVECs with shRNA targeting NOTCH3 or negative control shRNA, and we verified NOTCH3 knockdown in HUVECs using Western blotting and RT-qPCR (Figure 6B). Levels of mRNAs encoding all these factors tended to decrease in the presence of NOTCH3 knockdown, but the decrease was significant only in the case of IA-related and angiogenesis factors MMP9, NF-κB, MCP1, IL-6 and VCAM-1 (Figure 6C). However, no noticeable differences in these significantly decreased angiogenesis factors were found between IA and healthy artery (Supplementary Figure 1).

## DISCUSSION

In this study, we identified *NOTCH3* as a key IA-related gene through a series of bioinformatic analyses followed by validation with IA patients’ samples and cell culture. First, we analyzed next-generation sequence data and discovered 6649 deleterious SNPs in 4537 genes in IA patients. Second, we collected RNA-sequencing data from GEO datasets and identified 1403 DEGs associated with IA. Third, we focused on 369 genes shared between the genes that were mutated in our next-generation sequencing data and the DEGs from the GEO data. Finally, we took the intersection of four highly relevant IA-related GO-term genes and decided to focus on *NOTCH3* because of its previously defined role in the cerebral artery. We then performed immunohistochemical studies on IA and cerebral artery tissue and showed NOTCH3 down-regulation in IA. Further, we found that NOTCH3 knockdown down-regulated angiogenesis molecules in HUVECs. However, the mRNA expression of *NOTCH3* increases in peripheral blood neutrophil.

In this study, we found that IA may be associated with allele T of rs779314594 (P = 0.001), allele A of rs200504060 (P = 0.004), and allele T of rs2285981 (P = 0.004) in discovery cohort (Table 3). However, no significant enrichment was detected in the sporadic IA cases (Supplementary Table 2). It indicates that the detected SNPs of *NOTCH3* remain familial accumulations. They locate in the exons regions of *NOTCH3* gene, and may affect the function of protein, because the variations alter the amino acid residue of original protein so that the structure of the NOTCH3 protein was changed [28] (Figure 4). Additionally, this alteration was predicted be deleterious by bioinformatics tools GERP++ [47], SIFT [46], CADD [48], PolyPhen-2 [49], and MutationTaster [29] (Table 2).

**Table 3:** Single-nucleotide polymorphisms in NOTCH3 that are associated with intracranial aneurysm

**ID**	**Polymorphism**	**our study (risk allele/ normal allele)**	**GnomAD^a^ (risk allele/ normal allele)**	**Fisher's Exact Test**	**OR**	**95% CI**	
						**Lower**	**Upper**
rs779314594	T	T/C = 1/19	T/C = 14/223582	P<0.001	841	105	6715
rs200504060	A	A/G = 1/19	A/G = 237/245087	P<0.004	269	35	2048
rs2285981	T	T/C = 1/19	T/C = 48/245638	P<0.004	256	37	1765

^a^ Genome Aggregation Database (http://gnomad.broadinstitute.org/)

Damage to the cerebral artery is the induction factor for IA [30], and NOTCH3 plays a vital role in cerebral blood vessels. NOTCH3 is essential for the structural integrity of small distal elastic arteries. In NOTCH3-null mice, myogenic tone significantly decreases, and isolated cerebral and tail caudal arteries show increased flow-mediated dilation. NOTCH3 governs the reactivity of vessels to pressure, flow, and other mechanical factors via the RhoA/ROCK signaling pathway [31]. Recently, our team identified the guanine exchange factor ARHOGEF17 in the RhoA/ROCK signaling pathway as a risk factor for IA [32]. Furthermore, the Notch pathway cross-talks with important signaling networks involving angiotensin-2 (AngII), [33] TGFβ, [34] ALK1, [35] and VEGF [36]. All these pathways play essential roles in IA onset and formation. Therefore, our findings support the importance of NOTCH3 in IA development. Further studies should be performed to determine how reduced expression of NOTCH3 affects the cerebral artery as well as the downstream IA-related signaling pathways mentioned above. Our findings here are consistent with previous studies linking NOTCH3 to IA [37], and cerebral autosomal dominant arteriopathy with subcortical infarcts and leukoencephalopathy [25].

NOTCH3 can promote inflammation in some biological processes [38], and inflammatory cells play a crucial role in IA formation and rupture. We examined *NOTCH3* mRNA expression in neutrophils from the GEO database and found mRNA expression to be higher in IA neutrophils than in control neutrophils (Figure 6A). It may be that higher levels of NOTCH3 in neutrophils promote inflammation in the cerebral artery, injuring it and thereby leading to IA. We did not find direct connection between the expression level of NOTCH3 and the patient prognosis in our study. Nevertheless, rupture of IA is the most severe prognostic indicator for IA patient, and the risk of aneurysm rupture is associated with the degree of inflammation in the arterial wall, which in turn could be aggravated by upregulated NOTCH3 in neutrophils [39, 40]. In addition to inflammation, altered NOTCH3 expression may contribute to IA by influencing angiogenesis damaging cerebrovascular endothelial repair. We found that NOTCH3 knockdown in HUVECs significantly down-regulated MMP9, NF-κB, MCP1, IL-6 and VCAM-1. These factors help regulate angiogenesis under homeostatic conditions [41], and an imbalance of angiogenesis factors is associated with IA [42]. We extracted the expression levels of impacted genes by NOTCH3 knockdown in HUVEC of from the GEO dataset but no noticeable difference was found between IA and healthy artery (Supplementary Figure 1). The authors deem that the possible cause is that these decreased changes of IA-related factors are mainly in cerebral vessel endothelium but not smooth muscle, and these changes cannot be shown in the sequencing of the complete IA and healthy artery. In short, distinct expression of NOTCH3 in IA tissue vs. neutrophils may have differential implication in IA: down-regulated NOTCH3 in IA tissue disrupts angiogenesis and cerebrovascular endothelial repair [43]; up-regulated NOTCH3 in neutrophils promotes inflammation and damage cerebral vessels directly.

**Figure 7:**
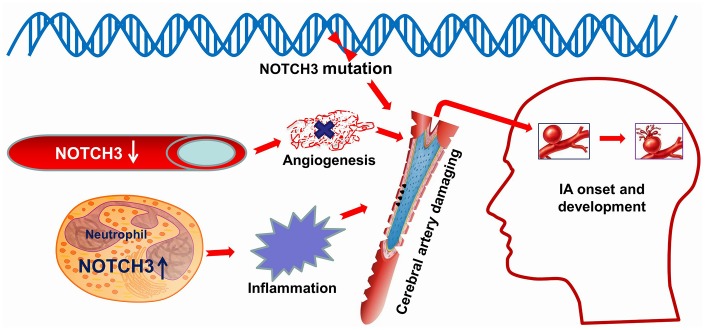
Schematic illustrating a possible role for NOTCH3 in IA. NOTCH3 is altered at the genetic polymorphisms which were predicted to alter an amino acid residue and therefore potentially the protein structure. Down-regulated expression of NOTCH3 in the cerebral artery influences angiogenesis damaging cerebrovascular endothelial repair. Up-regulated expression of NOTCH3 in neutrophil activate and promote inflammation in the cerebral artery. In a word, these abnormities in NOTCH3 cause damage to blood vessels in the brain, which can lead to the onset and development of intracranial aneurysm. Image modified from ScienceSlides (VisiScience Corp., North Carolina, USA).

In conclusion, we think that variations in *NOTCH3* and dysregulation of its expression (down-regulation in cerebral artery, up-regulation in neutrophils) contribute to IA, perhaps directly as well as indirectly by affecting the levels of downstream IA-related factors (Figure 7). For precision medicine [44], our work identifies NOTCH3 as a novel target for treating or even preventing IA. Further research is needed to clarify how NOTCH3 participates in IA formation and development.

## MATERIALS AND METHODS

### Patient information and next-generation sequence data

All study participants signed informed consent before enrollment. The discovery cohort for this study included 20 Chinese individuals diagnosed with IA at Tianjin Medical University General Hospital (Figure 1A and 1B). Ten individuals had unruptured IAs diagnosed by computed tomography angiography or digital subtraction angiography. The remaining ten subjects had subarachnoid hemorrhage caused by IA rupture, which was confirmed by computed tomography angiography, surgery and clinical symptoms or signs. Among the 20 patients, 19 were familial cases, while one was a sporadic case.

Patient blood samples were collected and immediately preserved in the −80 °C freezer until further use. Samples were used for whole-genome sequencing (n=10) (Novogene, Beijing, China) and whole exome sequencing (n=10) (BerryGenomics, Beijing, China) (Figure 1C). Variation sites were annotated and filtered based on functional changes by SAMtools, ANNOVAR, PolyPhen-2 and SIFT software [45–48]. We adapted broadly used bioinformatics tools including GERP++, [49] SIFT, [48] CADD, [50] PolyPhen-2, [51] and MutationTaster to predict the functional effects of missense variants [29, 52]. As long as one of the above tools predicts the harmfulness of the SNP, we assume that this variation site is deleterious SNP. All genes with de deleterious SNPs were screened for combining with the following transcriptome sequencing data to find new IA-related genes.

### Composition and analysis of transcriptome sequencing data

We obtained high-throughput RNA data from 129 samples across eight series of data for IA and cerebral artery tissue from the Gene Expression Omnibus (GEO) (Supplementary Table 3). We also obtained high-throughput RNA data from peripheral blood neutrophils of IA patients and controls from the GEO (https://www.ncbi.nlm.nih.gov/geo, GSE106520).

Differentially expressed genes (DEG) between IA and healthy cerebral artery tissue were identified using R and Bioconductor [53, 54]. First, we used the *limma* package (version 3.8) with batch normalization to integrate different data platforms into one matrix [55]. Then all the samples from the combined matrix were standardized (Supplementary Figure 2) and DEGs were determined using the Empirical Bayes method [56]. Statistically significant DEGs were defined as p<0.05 and |logFC| > 1 (logFC, Fold change between IA and healthy cerebral artery).

### Comprehensive data mining

To identify likely IA-related genes, we combined the next-generation sequencing data and high-throughput RNA data and analyzed them using BiNGO (Cytoscape Biological Networks, Version 3.0.3), a Gene Ontology (GO) program that assesses the overrepresentation of GO categories in a biological network [23]. DEGs were analyzed using the Benjamini & Hochberg False Discovery Rate (FDR) correction [57]. P-value < 0.05 was used to define significant enrichment. This analysis identified the following enriched IA-related GO-term genes: GO-6950, response to stress; GO-7399, nervous system development; GO-2376, immune system process; and GO-48856, anatomical structure development. These subcategories were checked for overlap with mutant genes discovered from our next-generation sequencing in order to identify shared IA-related gene(s).

### Identified SNPs of NOTCH3 was tested in sporadic IA patients

In order to ascertain the association between identified SNPs of NOTCH3 and sporadic IA patients, we performed KASP (Kompetitive Allele Specific PCR) for SNP genotype (BioMiao Biological Technology (Beijing) Co., Ltd). The primers and probes were designed by the Laboratory of the Government Chemist (LGC) (Supplementary Table 4). The DNA of blood samples of IA patients (n=594) and control subjects (n=600) sourced from our previous studies [32, 58]. The Sequencing data was analyzed by PLINK (Version 1.9) [59]. Multiple-testing was performed in PLINK to make the statistics more exact.

### Preparation and immunohistochemistry of IA and healthy cerebral artery specimens

IA specimens were donated by three patients who underwent clipping at Fuzhou Second Hospital Affiliated to Xiamen University. All three subjects signed informed consent forms. The sample acquisition was approved by IRB (SQ2018-003). After the aneurysm was securely clipped, a small piece of tissue was removed from the IA crest and considered as IA wall tissue. Cerebral artery samples from the control group were taken from autopsies performed in the Department of Pathology at Fuzhou Second Hospital Affiliated to Xiamen University. The two sets of specimens were immediately stored at −80°C (Thermo Scientific™, Shanghai, China) and processed for experimentation within one month. Tissues were fixed in 4% paraformaldehyde for 24 hours, paraffin-embedded, and sliced to a thickness of 4 μm. Specimens underwent antigen retrieval in 10 mM sodium citrate (pH 6.0) containing 0.05% Tween 20 for 3 min at maximum strength in a pressure cooker, then allowed to cool to room temperature. This was followed by blocking with 5% goat serum for 30 min at 37 °C to prevent nonspecific staining. The samples were subsequently incubated overnight at 4 °C with anti-NOTCH3 primary antibody (1:300; Abcam, Cambridge, UK, catalog no. ab23426). After 16 hours, the samples were rinsed with TBST and then incubated for 1 h at room temperature with secondary antibodies. DAB chromogen was added for 4 min to a final concentration of 0.05%, and slices were sealed with neutral resin. Finally, the sections were observed and images captured with an inverted microscope (OLYMPUS, Japan).

Before fixation, a small piece of tissue was taken for genotyping rs779314594, rs200504060, and rs2285981 of NOTCH3 by Sanger sequencing (BioMiao Biological Technology, Beijing, China) [60]. Clinical data for these samples are provided in the Supplementary Data (Supplementary Table 5).

### Culture of HUVECs

HUVECs (ScienCell, California, US, catalog no. 8000) were maintained in Endothelial Cell Medium (ScienCell, California, US, catalog no. 1001) supplemented with 1% endothelial growth supplement and 5% fetal bovine serum (FBS) at 37 °C in an incubator with 95% humidified air and 5% CO_2_. Subculture was performed when the cells reached 90–95% confluency. Cells within five passages were used for *in* *vitro* studies.

### Lentiviral transfection

Recombinant lentivirus was transfected into HUVECs using the Lentivirus transfection system (Hanbio, Shanghai, China) according to the manufacturer’s instructions. For lentivirus construction, short hairpin (sh)RNA clones were inserted into pHBLV-U6-MCS-CMV-ZsGreen-PGK-PURO puromycin lentiviral vectors (Hanbio, Shanghai, China). Cells were infected with the virus in the presence of Polybrene (Sigma-Aldrich, Missouri, USA). At 48h later, HUVECs were cultured in medium containing puromycin for the selection of stable clones. Clones in which NOTCH3 was stably knocked down were selected and verified by Western blotting and RT-qPCR. The following previously published shRNA sequences were used [61]:

NOTCH3 top strand,

GATCCGGGGGACCTGCCGTGGCTATATTCAAGAGATATAGCCACGGCAGGTCCCCCTTTTTTG;

NOTCH3 bottom strand,

AATTCAAAAAAGGGGGACCTGCCGTGGCTATATCTCTTGAATATAGCCACAGGTCCCCCG;

negative control sequence top strand,

GATCCGGGGGACCTGCCGTGGCTATATTCAAGAGATATAGCCACGGCAGGTCCCCCTTTTTTG;

negative control bottom strand,

AATTCAAAAAAGGGGGACCTGCCGTGGCTATATCTCTTGAATATAGCCACGCAGGTCCCCCG.

### RT-qPCR

Total RNA was extracted from cells using Trizol reagent (Invitrogen, Carlsbad, CA, USA) as described by the manufacturer’s instructions. Reverse transcription was performed using a Reverse Transcription Kit (Promega, Shanghai, China). All reactions for real-time PCR were carried out in triplicate in a Bio-Rad Cycler system (Thousand Oaks, California, USA) using the SYBR Premix (Madison, Wisconsin, USA) and analyzed using the 2ΔΔ cycle threshold method. Levels of mRNA were presented relative to those for GAPDH. All primers were sourced from PrimerBank (https://pga.mgh.harvard.edu/primerbank/) [62]. Primer sequences are provided in the Supplementary Data (Supplementary Table 6).

### Statistical analysis

Statistical comparisons of two groups were analyzed using Student’s *t*-test. Statistical analyses of allele T of rs779314594, allele A of rs200504060, and allele T of rs2285981 was performed by Fisher’s test. A value of P<0.05 was regarded as significant. All analyses were performed using SPSS 22.0 (64-bit edition, IBM, Chicago, IL, USA).

### Ethical approval

Subjects (or their parents or guardians) have given their written informed consent.

The research institute’s committee has approved the study protocol of population genetics on human research (20170035). Immunohistochemistry of IA and healthy cerebral artery specimens was also approved (SQ2018-003).

## Supplementary Material

Supplementary Figures

Supplementary Tables

Supplementary Table 1

Supplementary Table 3
