# Supplementary material for: Genetic polymorphisms and transcription profiles associated with intracranial aneurysm: a key role for NOTCH3
Source: Aging (Albany NY). 2019 Jul 23;11(14):5173–91. doi: 10.18632/aging.102111 (PMC6682524; doi:10.18632/aging.102111)
Supplement: Supplementary Figures [file aging-11-102111-s004.pdf]

SUPPLEMENTARY FIGURES

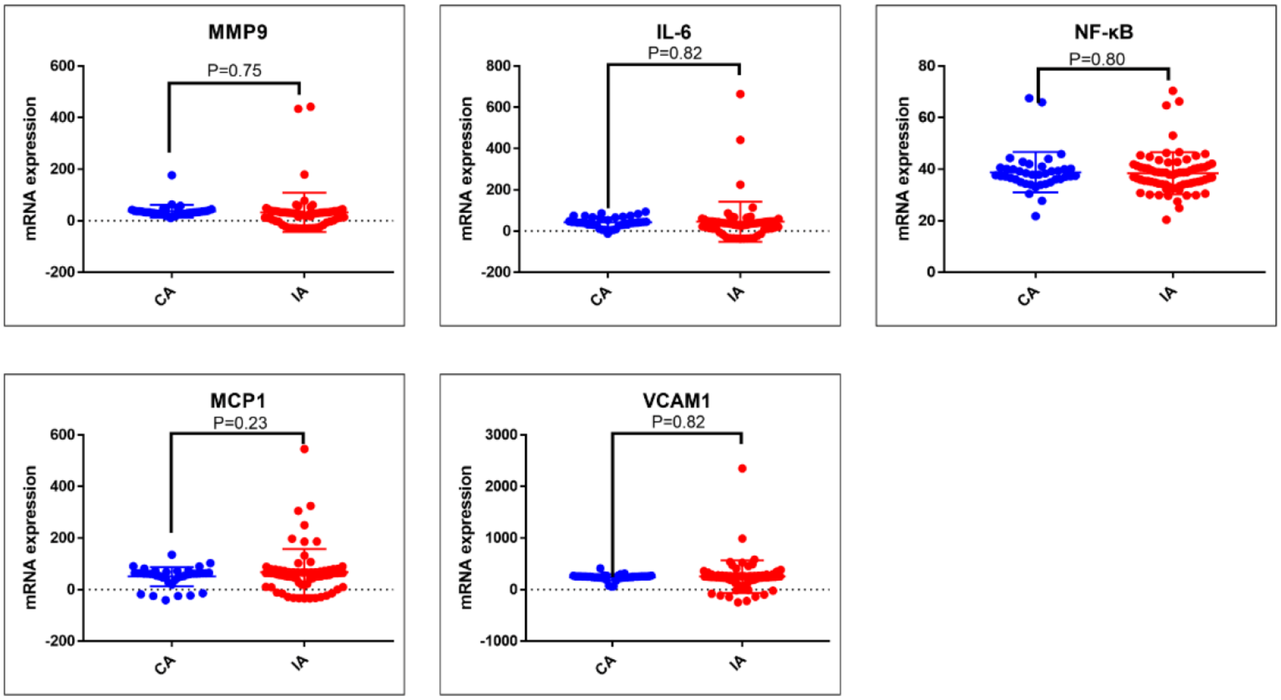

Supplementary Figure 1. The impacted IA-related angiogenesis factors by NOTCH3 Knockdown in HUVEC in IA and cerebral artery.

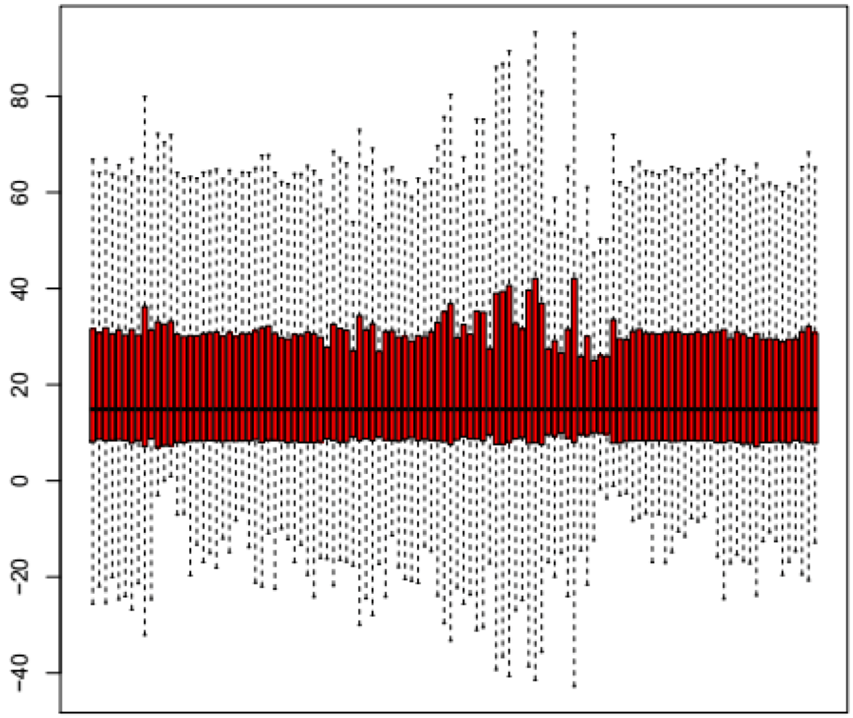

Supplementary Figure 2. Normalization of between arrays.

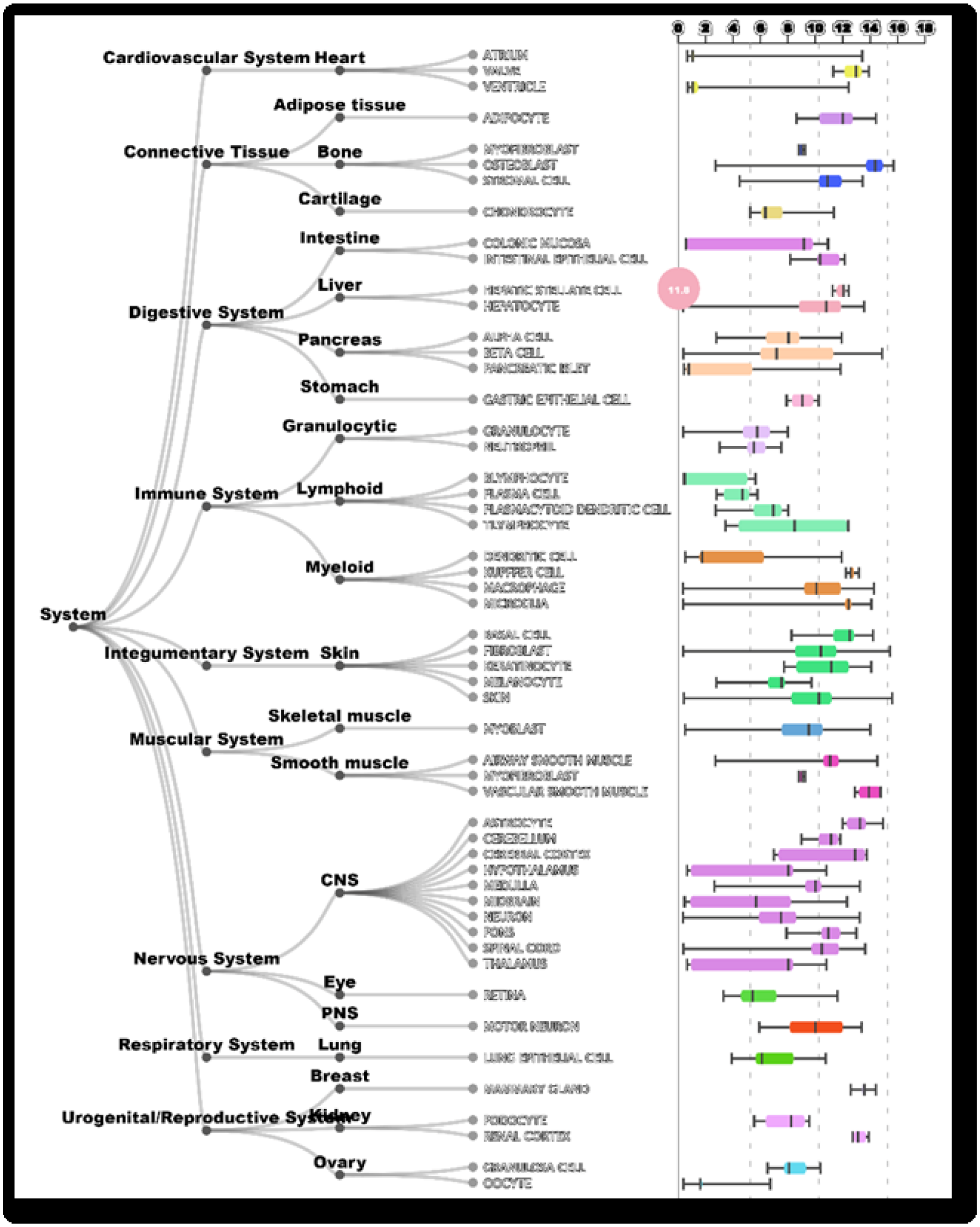

Supplementary Figure 3. NOTCH3 expression in different organs.

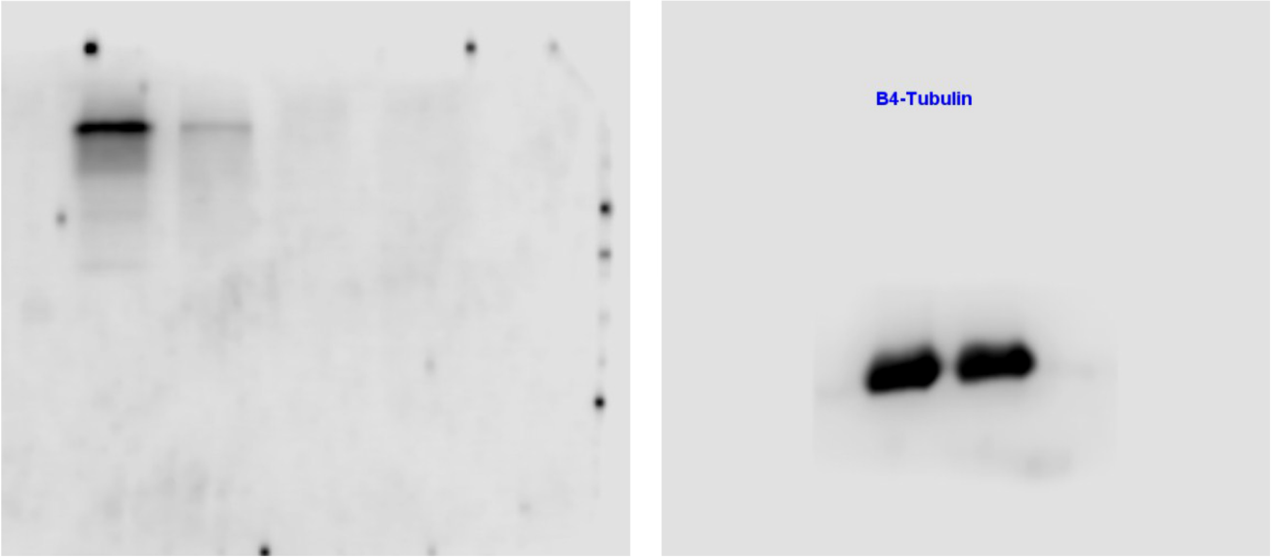

Supplementary Figure 4. The original whole full-length and uncropped image of western results of Figure 6.
