# Supplementary material for: Genetic polymorphisms and transcription profiles associated with intracranial aneurysm: a key role for NOTCH3
Source: Aging (Albany NY). 2019 Jul 23;11(14):5173–91. doi: 10.18632/aging.102111 (PMC6682524; doi:10.18632/aging.102111)
Supplement: Supplementary Tables [file aging-11-102111-s002.pdf]

SUPPLEMENTARY TABLES

Please browse Full Text version to see the data of:
Supplementary Table 1. DEGs between IA and healthy cerebral artery.

Supplementary Table 2. Rs779314594, rs200504060, rs2285981 in sporadic IA cases and control subjects

SNP	Mutant type	Wild type	F_A	IA group			Control group			OR	P	BONF
	A1	A2		A1A1	A1A2	A2A2	A1A1	A1A2	A2A2			
rs779314594	-	C	0	0	0	527	0	0	572	-	1	1
rs200504060	A	G	0.0158	0	17	520	0	13	572	1.44 (0.69–2.99)	0.33	0.99
rs2285981	T	C	0.005217	0	6	569	0	13	580	0.47 (0.18–1.25)	0.13	0.39

Note: A1 and A2 are allelic genes, A1 is the mutant type, A2 is wild type. F_A, Frequency of this allele in IA group. Abbreviations: SNP, single nucleotide polymorphism; IA, intracranial aneurysm; BONF, Bonferroni single-step adjusted P-values.

Please browse Full Text version to see the data of:
Supplementary Table 3. IA sample and cerebral artery sample of GEO.

Supplementary Table 4. KASP primers and probes.

	rs200504060	rs2285981	rs779314594
FAM-labelled primer	GGTGCCATTGTGTAGGCACCG	GTGTCCTGGACAGTCGTCCAC	ATCAACCCAGTGGGCCCCG
HEX-labelled primer	AGGTGCCATTGTGTAGGCACCA	GTGTCCTGGACAGTCGTCCAT	GATCAACCCAGTGGGCCCCA
universal primer	GGTGCTCTGCGAGATTAATGAGGAT	CTGCTAGGGTTTGAGGGTCAGAATT	AGCAGAGGCCCCAGGCCGT

Supplementary Table 5. Clinical data of IA samples and CA samples.

Sample	Age	Sex	location	Genotype		
				rs779314594	rs200504060	rs2285981
IA sample 1	53	Female	AcoA	CC	GG	CC
IA sample 2	60	Male	MCA	CC	GG	CC
IA sample 3	69	Male	MCA	CC	GG	CC
CA sample 1	68	Female	MCA	CC	GG	CC
CA sample 2	73	Male	MCA	CC	GG	CC
CA sample 2	65	Female	MCA	CC	GG	CC

Supplementary Table 6. Primers of IA-related factors tested in HUVEC.

H-IL-1beta_F-132	ATGATGGCTTATTACAGTGGCAA
H-IL-1beta_R-132	GTCGGAGATTCGTAGCTGGA
H-IL6_F-149	ACTCACCTCTTCAGAACGAATTG
H-IL6_F-149	CCATCTTTGGAAGGTTCAGGTTG
H-MCP-1_F-190	CAGCCAGATGCAATCAATGCC
H-MCP-1_F-190	TGGAATCCTGAACCCACTTCT
H-TNF-α_F-220	CCTCTCTCTAATCAGCCCTCTG
H-TNF-α_R-220	GAGGACCTGGGAGTAGATGAG
H-MMP-2_F-90	TACAGGATCATTGGCTACACACC
H-MMP-2_R-90	GGTCACATCGCTCCAGACT
H-MMP-9_F-97	TGTACCGCTATGGTTACACTCG
H-MMP-9_R-97	GGCAGGGACAGTTGCTTCT
H-NF-κB_F-104	AACAGAGAGGATTTCGTTTCCG
H-NF-κB_R-104	TTTGACCTGAGGGTAAGACTTCT
H-VCAM1_F-89	GGGAAGATGGTCGTGATCCTT
H-VCAM1_R-89	TCTGGGGTGGTCTCGATTTTA
NOTCH3_F-122	CGTGGCTTCTTTCTACTGTGC
NOTCH3_R-122	CGTTCACCGGATTTGTGTCAC
