# Supplementary material for: Genetic polymorphisms and transcription profiles associated with intracranial aneurysm: a key role for NOTCH3
Source: Aging (Albany NY). 2019 Jul 23;11(14):5173–91. doi: 10.18632/aging.102111 (PMC6682524; doi:10.18632/aging.102111)
Supplement: Supplementary Table 1 [file aging-11-102111-s003.docx]

**Supplementary Table 1. DEGs between IA and healthy cerebral artery.**

| **Gene Names** | **logFC** | **AveExpr** | **t** | **P.Value** | **adj.P.Val** | **B** |
| --- | --- | --- | --- | --- | --- | --- |
| RRBP1 | -6.19882 | 19.36782 | -7.50126 | 1.68E-11 | 1.65E-07 | 15.7006051 |
| CAD | -2.65728 | 12.22587 | -7.09428 | 1.29E-10 | 6.37E-07 | 13.7803249 |
| GALNT5 | -8.07037 | 12.64123 | -6.97746 | 2.31E-10 | 7.58E-07 | 13.235722 |
| TNF | -2.70249 | 8.109974 | -6.8883 | 3.58E-10 | 8.82E-07 | 12.8222845 |
| MOCOS | -1.34431 | 7.24892 | -6.80668 | 5.35E-10 | 8.94E-07 | 12.4455286 |
| CD80 | -2.6103 | 8.107435 | -6.80302 | 5.45E-10 | 8.94E-07 | 12.4286684 |
| IRF5 | -5.00192 | 12.38498 | -6.72707 | 7.89E-10 | 1.11E-06 | 12.0797347 |
| EGR4 | -4.8871 | 12.12149 | -6.67525 | 1.02E-09 | 1.13E-06 | 11.8425029 |
| XRCC4 | -2.49939 | 11.13246 | -6.66426 | 1.07E-09 | 1.13E-06 | 11.7923211 |
| P2RY6 | -2.95269 | 11.58852 | -6.6504 | 1.15E-09 | 1.13E-06 | 11.7290229 |
| TRPM2 | -3.05094 | 10.42414 | -6.58761 | 1.55E-09 | 1.39E-06 | 11.4430311 |
| KCNK6 | -3.11627 | 11.42061 | -6.56429 | 1.74E-09 | 1.39E-06 | 11.3370894 |
| GPR132 | -3.06355 | 7.855779 | -6.55283 | 1.84E-09 | 1.39E-06 | 11.2850858 |
| CECR6 | -2.5866 | 10.08073 | -6.48503 | 2.55E-09 | 1.79E-06 | 10.9781551 |
| FSCN1 | -4.51413 | 17.79146 | -6.4504 | 3.01E-09 | 1.95E-06 | 10.8219574 |
| SYNGR1 | -3.14106 | 13.91223 | -6.43579 | 3.23E-09 | 1.95E-06 | 10.7561332 |
| ARNTL2 | -4.3422 | 9.935308 | -6.4275 | 3.36E-09 | 1.95E-06 | 10.7188297 |
| LILRB4 | -6.01437 | 10.33217 | -6.40925 | 3.67E-09 | 2.01E-06 | 10.6367508 |
| LIMK1 | -3.93935 | 13.3898 | -6.34874 | 4.89E-09 | 2.54E-06 | 10.3654129 |
| EBI3 | -3.10544 | 10.43961 | -6.33658 | 5.19E-09 | 2.55E-06 | 10.3110097 |
| GPR84 | -2.76663 | 7.98497 | -6.25777 | 7.54E-09 | 3.54E-06 | 9.95959886 |
| NF2 | -2.75262 | 13.32531 | -6.18723 | 1.05E-08 | 4.71E-06 | 9.64675726 |
| CDH19 | 13.64266 | 18.13699 | 6.13346 | 1.36E-08 | 5.80E-06 | 9.40940319 |
| NAGS | -2.48537 | 9.337268 | -6.10195 | 1.57E-08 | 6.45E-06 | 9.27074038 |
| WNT4 | -5.42359 | 14.54082 | -6.06767 | 1.84E-08 | 7.26E-06 | 9.12030539 |
| NPY1R | 10.40828 | 12.11803 | 5.965593 | 2.97E-08 | 1.12E-05 | 8.67473556 |
| KIAA0391 | -1.96838 | 9.034479 | -5.944 | 3.28E-08 | 1.20E-05 | 8.58093588 |
| PILRA | -4.63712 | 12.10825 | -5.90296 | 3.96E-08 | 1.39E-05 | 8.40319836 |
| GDF15 | -6.9707 | 11.82068 | -5.87894 | 4.42E-08 | 1.50E-05 | 8.29939645 |
| MCOLN2 | -3.01556 | 8.932601 | -5.83805 | 5.34E-08 | 1.73E-05 | 8.12327616 |
| NGFR | 5.135728 | 10.76276 | 5.834118 | 5.44E-08 | 1.73E-05 | 8.10636132 |
| CENPE | -1.47503 | 6.672473 | -5.80231 | 6.29E-08 | 1.94E-05 | 7.9698265 |
| PC | -5.6083 | 13.79027 | -5.79279 | 6.57E-08 | 1.96E-05 | 7.92904244 |
| MATK | -4.19555 | 11.59051 | -5.77298 | 7.19E-08 | 2.03E-05 | 7.84424622 |
| IL21R | -4.76103 | 11.14301 | -5.7721 | 7.22E-08 | 2.03E-05 | 7.84049765 |
| CACNB4 | -1.92937 | 9.560262 | -5.76057 | 7.61E-08 | 2.08E-05 | 7.7911983 |
| FDXR | -4.79282 | 13.35105 | -5.75477 | 7.81E-08 | 2.08E-05 | 7.76647154 |
| CASP5 | -1.49132 | 7.410164 | -5.72653 | 8.89E-08 | 2.30E-05 | 7.64605142 |
| SIGLEC7 | -4.59226 | 12.46943 | -5.71357 | 9.42E-08 | 2.38E-05 | 7.59092415 |
| ADCYAP1 | -2.24023 | 7.884502 | -5.6771 | 1.11E-07 | 2.68E-05 | 7.43608456 |
| GPR143 | -1.52304 | 7.001236 | -5.66981 | 1.15E-07 | 2.68E-05 | 7.40520378 |
| DGKI | -2.07765 | 7.660446 | -5.66929 | 1.15E-07 | 2.68E-05 | 7.40297471 |
| LAMB3 | -2.17241 | 8.751535 | -5.66599 | 1.17E-07 | 2.68E-05 | 7.38900212 |
| NPM3 | -2.45796 | 11.44679 | -5.59486 | 1.61E-07 | 3.50E-05 | 7.08886441 |
| NME5 | -2.6625 | 9.491754 | -5.5885 | 1.66E-07 | 3.50E-05 | 7.06211651 |
| NUP210 | -4.70108 | 13.24333 | -5.58273 | 1.70E-07 | 3.50E-05 | 7.03787187 |
| PLEK2 | -5.70716 | 13.79482 | -5.58022 | 1.72E-07 | 3.50E-05 | 7.02734297 |
| LOXL3 | -10.1195 | 23.9063 | -5.57797 | 1.74E-07 | 3.50E-05 | 7.0178931 |
| CARD11 | -3.99466 | 11.73348 | -5.57103 | 1.79E-07 | 3.50E-05 | 6.98875841 |
| SMPD1 | -3.72543 | 15.99013 | -5.56298 | 1.86E-07 | 3.50E-05 | 6.95499527 |
| ST14 | -10.5456 | 14.53095 | -5.562 | 1.87E-07 | 3.50E-05 | 6.9508838 |
| ALX3 | -3.21387 | 13.13511 | -5.56136 | 1.87E-07 | 3.50E-05 | 6.94820324 |
| TOR2A | -2.02548 | 8.645433 | -5.55974 | 1.89E-07 | 3.50E-05 | 6.94140788 |
| CTLA4 | -1.90593 | 7.656821 | -5.54788 | 1.99E-07 | 3.63E-05 | 6.89173374 |
| CDCA3 | -3.58984 | 11.54541 | -5.53771 | 2.08E-07 | 3.73E-05 | 6.84921234 |
| CACNA2D4 | -2.83666 | 10.81415 | -5.52912 | 2.16E-07 | 3.80E-05 | 6.8132879 |
| APOC2 | -4.14351 | 9.194606 | -5.5176 | 2.28E-07 | 3.93E-05 | 6.76519332 |
| RAB39B | -2.14155 | 6.373971 | -5.49705 | 2.50E-07 | 4.24E-05 | 6.67951708 |
| TGFBRAP1 | -3.61491 | 18.53791 | -5.43892 | 3.23E-07 | 5.39E-05 | 6.43809775 |
| CDCA8 | -2.53749 | 9.458472 | -5.40021 | 3.83E-07 | 6.14E-05 | 6.27815639 |
| ZIC3 | -1.90698 | 7.488746 | -5.39833 | 3.86E-07 | 6.14E-05 | 6.27038968 |
| PARVB | -3.44598 | 15.80449 | -5.39815 | 3.87E-07 | 6.14E-05 | 6.26966719 |
| VIT | 4.968297 | 11.46909 | 5.372382 | 4.33E-07 | 6.77E-05 | 6.16355149 |
| VGF | -2.69518 | 11.97912 | -5.36458 | 4.48E-07 | 6.90E-05 | 6.13149039 |
| CPT1C | -2.01213 | 9.395074 | -5.30672 | 5.78E-07 | 8.72E-05 | 5.89448897 |
| KCTD5 | -2.5069 | 14.19908 | -5.2964 | 6.05E-07 | 8.72E-05 | 5.85237435 |
| CDKN2A | -4.18359 | 10.74302 | -5.29581 | 6.06E-07 | 8.72E-05 | 5.84999118 |
| CUZD1 | -1.90248 | 8.092191 | -5.29474 | 6.09E-07 | 8.72E-05 | 5.84563273 |
| SALL1 | -5.09193 | 7.658519 | -5.29387 | 6.11E-07 | 8.72E-05 | 5.84209487 |
| ZMYND15 | -3.28515 | 13.26334 | -5.28912 | 6.24E-07 | 8.78E-05 | 5.82272121 |
| TOX | 3.025658 | 9.945181 | 5.285721 | 6.33E-07 | 8.78E-05 | 5.80886642 |
| OSM | -7.25851 | 12.30303 | -5.27862 | 6.53E-07 | 8.88E-05 | 5.77995945 |
| ADORA3 | -10.3213 | 14.86642 | -5.2729 | 6.70E-07 | 8.88E-05 | 5.75665881 |
| MDM2 | -4.98738 | 17.14263 | -5.27072 | 6.76E-07 | 8.88E-05 | 5.74780107 |
| TSNAXIP1 | -1.95891 | 10.14222 | -5.26488 | 6.94E-07 | 8.88E-05 | 5.72405819 |
| KCNE3 | -5.159 | 10.97283 | -5.26452 | 6.95E-07 | 8.88E-05 | 5.72261223 |
| SLC13A4 | -9.35379 | 15.56856 | -5.26326 | 6.98E-07 | 8.88E-05 | 5.71748055 |
| RAB26 | -2.08718 | 8.393039 | -5.26179 | 7.03E-07 | 8.88E-05 | 5.71152963 |
| CGREF1 | -1.59214 | 7.576935 | -5.23786 | 7.80E-07 | 9.72E-05 | 5.61440566 |
| LDB2 | 4.9559 | 12.55403 | 5.232847 | 7.97E-07 | 9.81E-05 | 5.59410789 |
| CCIN | -1.76404 | 7.483141 | -5.22545 | 8.23E-07 | 0.00010008 | 5.56416989 |
| GLB1L | -4.54251 | 14.45338 | -5.20097 | 9.15E-07 | 0.00010991 | 5.46522871 |
| IL27RA | -2.4004 | 10.55769 | -5.19594 | 9.35E-07 | 0.00011097 | 5.444972 |
| KIF2C | -2.77955 | 10.95099 | -5.19173 | 9.52E-07 | 0.00011166 | 5.42799746 |
| ZIC4 | -1.14509 | 7.011645 | -5.1772 | 1.01E-06 | 0.00011749 | 5.36946481 |
| GGA2 | -5.1594 | 16.87829 | -5.17041 | 1.04E-06 | 0.00011958 | 5.34212887 |
| GFI1 | -2.20823 | 8.480064 | -5.16323 | 1.08E-06 | 0.00012192 | 5.31328537 |
| ADORA1 | -2.30952 | 9.727113 | -5.1588 | 1.10E-06 | 0.00012285 | 5.29550598 |
| FKBP10 | -6.55316 | 19.44846 | -5.15181 | 1.13E-06 | 0.00012518 | 5.26743698 |
| SDC1 | -5.62099 | 21.60606 | -5.13179 | 1.23E-06 | 0.00013491 | 5.18718529 |
| CAMK1G | -1.45945 | 6.571326 | -5.12008 | 1.30E-06 | 0.0001403 | 5.14032958 |
| FMO5 | -1.09126 | 7.556356 | -5.11294 | 1.34E-06 | 0.00014309 | 5.11180688 |
| SIGLEC9 | -3.24571 | 9.307709 | -5.09047 | 1.47E-06 | 0.00015546 | 5.02216618 |
| TREM2 | -16.3074 | 19.57435 | -5.08853 | 1.48E-06 | 0.00015546 | 5.01443704 |
| GCAT | -2.68359 | 12.74991 | -5.07383 | 1.58E-06 | 0.00016378 | 4.95594279 |
| LIPH | -2.76677 | 7.617036 | -5.0623 | 1.66E-06 | 0.00017024 | 4.91015549 |
| MRPS12 | -1.81853 | 9.89009 | -5.0488 | 1.76E-06 | 0.00017845 | 4.85660672 |
| EYA2 | -4.39264 | 12.53469 | -5.04054 | 1.82E-06 | 0.00018165 | 4.82387087 |
| IL12A | -1.04373 | 6.032479 | -5.03982 | 1.83E-06 | 0.00018165 | 4.82101965 |
| PIP | -1.99207 | 8.441923 | -5.03118 | 1.89E-06 | 0.00018479 | 4.78681638 |
| RERG | 9.2562 | 13.85389 | 5.027031 | 1.93E-06 | 0.00018479 | 4.77041587 |
| NFE2L3 | -1.55723 | 7.592776 | -5.02647 | 1.93E-06 | 0.00018479 | 4.76818355 |
| SLAMF6 | -4.01363 | 12.28361 | -5.02645 | 1.93E-06 | 0.00018479 | 4.76811487 |
| NEDD4L | -2.07017 | 9.27508 | -5.01627 | 2.02E-06 | 0.00019109 | 4.72788342 |
| AP1B1 | -7.91806 | 24.49506 | -4.99183 | 2.24E-06 | 0.00020989 | 4.63154597 |
| SAMD3 | -1.41945 | 6.169279 | -4.98804 | 2.27E-06 | 0.00021084 | 4.61665189 |
| ATP6V0D2 | -2.39837 | 8.014631 | -4.9843 | 2.31E-06 | 0.00021084 | 4.6019326 |
| GPAA1 | -5.03816 | 34.12506 | -4.9841 | 2.31E-06 | 0.00021084 | 4.6011384 |
| C14orf79 | -3.80225 | 15.83829 | -4.96887 | 2.47E-06 | 0.00022276 | 4.54131901 |
| ASB2 | 5.271594 | 17.15251 | 4.963346 | 2.52E-06 | 0.00022594 | 4.51962718 |
| RYR2 | 11.55409 | 16.06303 | 4.949893 | 2.67E-06 | 0.00023494 | 4.4668999 |
| KIF14 | -1.55875 | 6.403491 | -4.94978 | 2.67E-06 | 0.00023494 | 4.46645409 |
| SGPL1 | -8.41724 | 23.39165 | -4.94305 | 2.75E-06 | 0.00023955 | 4.44012102 |
| CAPNS2 | -1.45101 | 6.532077 | -4.94013 | 2.78E-06 | 0.00024037 | 4.42870964 |
| ARHGAP11A | -1.3559 | 5.092577 | -4.92912 | 2.91E-06 | 0.00024956 | 4.38567714 |
| ERCC6 | -3.29603 | 12.60224 | -4.91773 | 3.06E-06 | 0.00025867 | 4.34119394 |
| PLK4 | -1.45047 | 6.810128 | -4.91646 | 3.07E-06 | 0.00025867 | 4.33626479 |
| LAMA1 | -1.30859 | 5.994082 | -4.90025 | 3.29E-06 | 0.00027448 | 4.27311868 |
| UTF1 | -2.47601 | 12.62129 | -4.88265 | 3.54E-06 | 0.00029038 | 4.20472552 |
| PPL | 2.529133 | 9.005323 | 4.880759 | 3.57E-06 | 0.00029038 | 4.19736581 |
| KYNU | -7.33087 | 12.92573 | -4.87698 | 3.62E-06 | 0.00029258 | 4.1826903 |
| LILRB1 | -5.68407 | 12.86111 | -4.87475 | 3.66E-06 | 0.00029291 | 4.17406371 |
| IL1A | -1.1227 | 5.839943 | -4.84836 | 4.08E-06 | 0.00032428 | 4.07188158 |
| ABCA5 | 2.846787 | 10.10552 | 4.839358 | 4.24E-06 | 0.00033207 | 4.03710161 |
| TNFSF9 | -1.46847 | 8.541927 | -4.8388 | 4.25E-06 | 0.00033207 | 4.03493011 |
| GCNT1 | -2.49096 | 10.38868 | -4.83247 | 4.36E-06 | 0.00033821 | 4.01053804 |
| PTPRH | -1.66751 | 9.137935 | -4.82981 | 4.41E-06 | 0.0003393 | 4.00025953 |
| CCR2 | -3.26008 | 7.870445 | -4.82789 | 4.45E-06 | 0.00033936 | 3.99286631 |
| CDH3 | -4.47116 | 11.13294 | -4.82037 | 4.59E-06 | 0.00034362 | 3.96389119 |
| ZBED4 | -1.38377 | 8.140059 | -4.81993 | 4.59E-06 | 0.00034362 | 3.96218354 |
| B3GAT3 | -2.99969 | 14.92982 | -4.81933 | 4.61E-06 | 0.00034362 | 3.95989696 |
| SOX11 | -6.00633 | 10.38184 | -4.81449 | 4.70E-06 | 0.00034795 | 3.94124356 |
| DIRAS2 | -3.17502 | 7.405369 | -4.81125 | 4.76E-06 | 0.00035001 | 3.92877853 |
| DEF6 | -6.30238 | 14.44011 | -4.79769 | 5.04E-06 | 0.00036746 | 3.87666502 |
| MMRN2 | 4.968299 | 13.43791 | 4.795329 | 5.09E-06 | 0.00036832 | 3.86762197 |
| GFRA4 | -1.86481 | 9.207088 | -4.78485 | 5.31E-06 | 0.00038179 | 3.82742824 |
| SYT3 | -1.26451 | 7.537695 | -4.78175 | 5.38E-06 | 0.0003839 | 3.81554719 |
| TEC | -1.69515 | 7.945686 | -4.77532 | 5.52E-06 | 0.00039137 | 3.79093719 |
| HMGB3 | -3.91479 | 12.50623 | -4.77198 | 5.60E-06 | 0.00039173 | 3.77815269 |
| IL2RA | -2.50243 | 8.131761 | -4.77163 | 5.61E-06 | 0.00039173 | 3.77681632 |
| ITLN1 | 22.3507 | 17.69478 | 4.767995 | 5.69E-06 | 0.00039367 | 3.76290474 |
| PDCD1LG2 | -1.66509 | 7.76946 | -4.76701 | 5.72E-06 | 0.00039367 | 3.75913971 |
| TBXAS1 | -5.54858 | 12.9501 | -4.76373 | 5.79E-06 | 0.00039625 | 3.74658533 |
| GEMIN6 | -2.25135 | 13.74058 | -4.76057 | 5.87E-06 | 0.00039666 | 3.7345245 |
| KLRD1 | -2.00426 | 8.857946 | -4.76012 | 5.88E-06 | 0.00039666 | 3.73280606 |
| SLC25A15 | -1.36258 | 7.944399 | -4.75659 | 5.97E-06 | 0.00039972 | 3.71932857 |
| ASPSCR1 | -1.75411 | 10.61048 | -4.75027 | 6.12E-06 | 0.00040747 | 3.6951948 |
| MUC2 | -2.035 | 8.844388 | -4.74663 | 6.22E-06 | 0.00041083 | 3.68131045 |
| ADCY5 | 4.863531 | 12.56778 | 4.733529 | 6.56E-06 | 0.00043062 | 3.63141394 |
| HIST1H2AM | -1.02417 | 6.124758 | -4.72726 | 6.73E-06 | 0.00043867 | 3.60756199 |
| GPA33 | -1.90925 | 8.284258 | -4.72577 | 6.77E-06 | 0.00043867 | 3.60190837 |
| CAMK1D | -3.78195 | 18.10421 | -4.72243 | 6.86E-06 | 0.00044181 | 3.58918931 |
| SIGLEC8 | -1.36035 | 8.300555 | -4.71929 | 6.95E-06 | 0.00044461 | 3.57728515 |
| TRDN | 1.717308 | 5.753293 | 4.71607 | 7.04E-06 | 0.00044629 | 3.56504376 |
| TFPT | -3.37007 | 14.78472 | -4.71521 | 7.07E-06 | 0.00044629 | 3.56179807 |
| SLC16A8 | -2.28038 | 9.58752 | -4.71265 | 7.14E-06 | 0.00044812 | 3.55207213 |
| ELOVL6 | -2.49797 | 9.634612 | -4.70313 | 7.43E-06 | 0.00046295 | 3.51595016 |
| PYCR1 | -4.76985 | 18.31433 | -4.70155 | 7.48E-06 | 0.00046302 | 3.50995363 |
| ADAM28 | -4.54212 | 11.37959 | -4.69848 | 7.57E-06 | 0.00046592 | 3.4983384 |
| APOL6 | -2.7136 | 12.20934 | -4.69684 | 7.62E-06 | 0.00046614 | 3.49211053 |
| SPRR1B | -1.70191 | 7.169707 | -4.6906 | 7.82E-06 | 0.00047519 | 3.46852225 |
| TNN | -2.50281 | 10.50139 | -4.68234 | 8.08E-06 | 0.00048844 | 3.43727356 |
| CHL1 | 6.936255 | 7.729779 | 4.680617 | 8.14E-06 | 0.00048888 | 3.43076479 |
| NXF5 | -1.16564 | 6.209563 | -4.67906 | 8.19E-06 | 0.000489 | 3.42489114 |
| CYP2S1 | -1.67111 | 8.702989 | -4.67281 | 8.40E-06 | 0.00049755 | 3.40126744 |
| HTR3A | -1.50287 | 6.895602 | -4.67046 | 8.48E-06 | 0.00049755 | 3.39240969 |
| PTK7 | -2.29887 | 11.53447 | -4.67038 | 8.49E-06 | 0.00049755 | 3.39209975 |
| CHN2 | -6.14349 | 12.57892 | -4.66803 | 8.57E-06 | 0.00049933 | 3.38326336 |
| ATP12A | -2.50803 | 10.99753 | -4.66629 | 8.63E-06 | 0.00049994 | 3.37667161 |
| RBKS | -2.3262 | 11.62336 | -4.65244 | 9.13E-06 | 0.00052575 | 3.32451209 |
| MPP7 | 9.424934 | 17.01986 | 4.650658 | 9.20E-06 | 0.00052649 | 3.31781055 |
| CLDN9 | -1.76608 | 7.960075 | -4.64809 | 9.29E-06 | 0.00052712 | 3.30813622 |
| CD6 | -3.36556 | 10.28214 | -4.64751 | 9.31E-06 | 0.00052712 | 3.30597763 |
| TGFA | -1.54747 | 7.861872 | -4.63721 | 9.71E-06 | 0.00054644 | 3.26727039 |
| CHEK2 | -2.22098 | 8.643717 | -4.63564 | 9.77E-06 | 0.00054679 | 3.26138754 |
| BCL2L11 | -4.80858 | 16.2789 | -4.6197 | 1.04E-05 | 0.0005799 | 3.20160216 |
| SDS | -9.09848 | 12.87574 | -4.61362 | 1.07E-05 | 0.00059099 | 3.17881681 |
| ISL2 | -1.72219 | 7.96252 | -4.61166 | 1.08E-05 | 0.00059186 | 3.17148384 |
| PPIL2 | -2.34379 | 14.66349 | -4.61041 | 1.08E-05 | 0.00059186 | 3.16682722 |
| RTN4RL1 | -2.18845 | 9.413626 | -4.60911 | 1.09E-05 | 0.00059186 | 3.16196392 |
| KCND1 | -2.34751 | 11.20997 | -4.60238 | 1.12E-05 | 0.00060074 | 3.13679238 |
| HIVEP3 | -2.1022 | 10.80307 | -4.60221 | 1.12E-05 | 0.00060074 | 3.13615018 |
| SLC12A5 | -4.21632 | 12.01974 | -4.60063 | 1.13E-05 | 0.00060074 | 3.13026454 |
| ADAM32 | -1.27481 | 7.362203 | -4.59999 | 1.13E-05 | 0.00060074 | 3.12787824 |
| TGM7 | -1.56159 | 6.360536 | -4.59334 | 1.16E-05 | 0.00061375 | 3.10303044 |
| POLQ | -2.01505 | 7.663494 | -4.59069 | 1.17E-05 | 0.00061405 | 3.0931567 |
| OXT | -2.271 | 10.9033 | -4.59037 | 1.17E-05 | 0.00061405 | 3.09197045 |
| ATRN | -2.67756 | 14.47646 | -4.58795 | 1.18E-05 | 0.00061405 | 3.08294287 |
| CHRNA4 | -1.48495 | 7.099477 | -4.58793 | 1.18E-05 | 0.00061405 | 3.0828493 |
| PTPN7 | -3.11765 | 10.70991 | -4.58376 | 1.20E-05 | 0.00062115 | 3.06733478 |
| AGMAT | -1.51539 | 8.278876 | -4.57828 | 1.23E-05 | 0.00063168 | 3.04691394 |
| ESPL1 | -2.39485 | 10.90614 | -4.57599 | 1.24E-05 | 0.00063417 | 3.03839932 |
| DHRS2 | -1.42689 | 7.690634 | -4.57269 | 1.26E-05 | 0.00063417 | 3.02610647 |
| NOL6 | -2.08352 | 11.85719 | -4.57034 | 1.27E-05 | 0.00063417 | 3.01737435 |
| PKD2L1 | -2.40547 | 11.26256 | -4.56979 | 1.27E-05 | 0.00063417 | 3.01532656 |
| ZNF215 | -1.23421 | 6.224666 | -4.56957 | 1.28E-05 | 0.00063417 | 3.01448682 |
| PPEF1 | -1.44722 | 7.109462 | -4.56648 | 1.29E-05 | 0.00063417 | 3.0029991 |
| FILIP1 | 7.346954 | 14.45338 | 4.566323 | 1.29E-05 | 0.00063417 | 3.00243043 |
| ABCA6 | 2.264342 | 9.266424 | 4.565625 | 1.30E-05 | 0.00063417 | 2.99983731 |
| PGM2L1 | -7.55726 | 20.1273 | -4.56448 | 1.30E-05 | 0.00063417 | 2.99558115 |
| SRPRB | -11.6761 | 34.43952 | -4.56423 | 1.30E-05 | 0.00063417 | 2.9946626 |
| PSEN1 | -3.52395 | 16.37345 | -4.56342 | 1.31E-05 | 0.00063417 | 2.99162913 |
| NEFH | -2.2852 | 8.308314 | -4.56076 | 1.32E-05 | 0.0006372 | 2.98177047 |
| GNGT2 | -1.75476 | 8.52047 | -4.55978 | 1.33E-05 | 0.0006372 | 2.97812956 |
| SLC6A7 | -2.97079 | 11.64472 | -4.55655 | 1.34E-05 | 0.00064238 | 2.96613023 |
| SLC22A9 | -1.31414 | 7.191334 | -4.55382 | 1.36E-05 | 0.00064629 | 2.95601171 |
| NMB | -5.02149 | 21.28535 | -4.54203 | 1.42E-05 | 0.00067103 | 2.91230634 |
| CCM2 | -10.0911 | 32.42796 | -4.53968 | 1.44E-05 | 0.00067104 | 2.90361182 |
| RASGRP4 | -2.07148 | 8.308516 | -4.53965 | 1.44E-05 | 0.00067104 | 2.9034774 |
| EGLN2 | -1.5016 | 10.47736 | -4.53072 | 1.49E-05 | 0.00069212 | 2.87044918 |
| SLC2A6 | -3.16617 | 11.35853 | -4.5224 | 1.54E-05 | 0.00071212 | 2.83970636 |
| SLC16A5 | -3.06334 | 11.51748 | -4.50991 | 1.62E-05 | 0.00074495 | 2.79363832 |
| ADAMTS10 | -7.48301 | 23.93217 | -4.50405 | 1.66E-05 | 0.00075564 | 2.77205534 |
| ADAMTS13 | -1.47069 | 9.440042 | -4.50399 | 1.66E-05 | 0.00075564 | 2.77182861 |
| BARHL1 | -2.3406 | 11.14875 | -4.50091 | 1.68E-05 | 0.00076143 | 2.76049148 |
| TMPRSS6 | -1.33529 | 8.758424 | -4.49966 | 1.69E-05 | 0.00076171 | 2.75589579 |
| DLG4 | -2.08376 | 8.831964 | -4.49416 | 1.72E-05 | 0.000775 | 2.73564668 |
| BTRC | -1.69803 | 10.53026 | -4.49235 | 1.74E-05 | 0.00077702 | 2.7290114 |
| SULT1B1 | -4.3468 | 10.3676 | -4.48796 | 1.77E-05 | 0.00078468 | 2.7128477 |
| KCNA7 | -1.28824 | 5.813638 | -4.48761 | 1.77E-05 | 0.00078468 | 2.71155813 |
| DISP1 | -1.40304 | 9.322909 | -4.4843 | 1.79E-05 | 0.00079147 | 2.69941925 |
| PRSS3 | -2.92758 | 11.19414 | -4.47587 | 1.85E-05 | 0.00081472 | 2.66848466 |
| SLC13A5 | -1.30089 | 6.921192 | -4.47468 | 1.86E-05 | 0.00081492 | 2.66413924 |
| CD1B | -1.40717 | 6.902717 | -4.46789 | 1.91E-05 | 0.00082972 | 2.63925158 |
| LMO4 | 4.842023 | 20.82621 | 4.467589 | 1.91E-05 | 0.00082972 | 2.63814212 |
| TNFSF14 | -3.22224 | 10.41656 | -4.46679 | 1.92E-05 | 0.00082972 | 2.63522994 |
| B4GALT1 | -5.28529 | 25.19163 | -4.46246 | 1.95E-05 | 0.00084038 | 2.61936692 |
| COL10A1 | -8.91383 | 13.10218 | -4.45808 | 1.99E-05 | 0.00085134 | 2.60334888 |
| HAMP | -6.53139 | 18.36048 | -4.45672 | 2.00E-05 | 0.00085221 | 2.59838645 |
| ABCB1 | -2.9566 | 11.7198 | -4.45521 | 2.01E-05 | 0.0008528 | 2.5928379 |
| FOXF2 | -7.25216 | 22.46457 | -4.45437 | 2.02E-05 | 0.0008528 | 2.58977958 |
| BATF | -5.15083 | 13.57628 | -4.45233 | 2.03E-05 | 0.00085368 | 2.58233245 |
| HOXA11 | -1.60835 | 6.561231 | -4.45194 | 2.04E-05 | 0.00085368 | 2.58091235 |
| LCT | -1.11938 | 5.744747 | -4.45086 | 2.05E-05 | 0.00085371 | 2.57695332 |
| KRTAP1-5 | -1.50395 | 8.102904 | -4.44968 | 2.06E-05 | 0.00085407 | 2.57264986 |
| GAL | -2.0365 | 8.577619 | -4.44332 | 2.11E-05 | 0.00086527 | 2.54944513 |
| VHL | -1.63221 | 12.28492 | -4.44319 | 2.11E-05 | 0.00086527 | 2.54896841 |
| FHOD3 | 3.659764 | 11.86041 | 4.441226 | 2.13E-05 | 0.00086839 | 2.54178921 |
| ERCC2 | -2.82122 | 14.86811 | -4.43473 | 2.18E-05 | 0.00088722 | 2.51811962 |
| KLHL13 | 2.273652 | 9.013367 | 4.433305 | 2.19E-05 | 0.00088856 | 2.51291512 |
| KCNK13 | -2.21878 | 8.973253 | -4.42795 | 2.24E-05 | 0.00089891 | 2.49341191 |
| SNX11 | -6.61715 | 33.88957 | -4.42725 | 2.25E-05 | 0.00089891 | 2.49085445 |
| IL12RB1 | -2.60867 | 10.00312 | -4.42458 | 2.27E-05 | 0.0009042 | 2.48116976 |
| GPC3 | 2.456731 | 8.413639 | 4.423697 | 2.28E-05 | 0.0009042 | 2.4779409 |
| IL9R | -1.22757 | 8.246404 | -4.41792 | 2.33E-05 | 0.00091604 | 2.45694132 |
| RAB38 | -2.57069 | 9.87437 | -4.4176 | 2.33E-05 | 0.00091604 | 2.45575737 |
| RGS6 | 4.375912 | 13.45253 | 4.417332 | 2.33E-05 | 0.00091604 | 2.45479921 |
| BRCA1 | -1.18347 | 8.109636 | -4.41552 | 2.35E-05 | 0.00091892 | 2.44822188 |
| OPLAH | -2.16013 | 9.962402 | -4.41382 | 2.37E-05 | 0.00092142 | 2.44203923 |
| OBP2A | -2.15571 | 10.80691 | -4.41011 | 2.40E-05 | 0.00092837 | 2.4285609 |
| MAGEL2 | -1.19948 | 7.209413 | -4.4099 | 2.40E-05 | 0.00092837 | 2.4278214 |
| POU6F2 | -1.20022 | 6.550872 | -4.40602 | 2.44E-05 | 0.00093893 | 2.41374488 |
| WT1 | -1.29854 | 7.053632 | -4.40392 | 2.46E-05 | 0.00094094 | 2.40611291 |
| SSTR5 | -1.80131 | 8.349387 | -4.40349 | 2.47E-05 | 0.00094094 | 2.40456841 |
| KCNK10 | -3.26206 | 7.900173 | -4.40185 | 2.48E-05 | 0.00094149 | 2.39861926 |
| APBA3 | -2.57214 | 13.8121 | -4.39632 | 2.54E-05 | 0.00095666 | 2.37857447 |
| FLT3 | -1.80523 | 8.134714 | -4.39337 | 2.56E-05 | 0.00096408 | 2.36790005 |
| SSTR2 | -1.72848 | 8.838627 | -4.38529 | 2.65E-05 | 0.0009856 | 2.33864856 |
| NEK6 | -4.15772 | 18.29311 | -4.38521 | 2.65E-05 | 0.0009856 | 2.33837753 |
| SIX4 | -4.42562 | 14.99203 | -4.38483 | 2.65E-05 | 0.0009856 | 2.3369757 |
| AIM1L | -1.71769 | 9.762013 | -4.37493 | 2.76E-05 | 0.00101724 | 2.30122844 |
| RASGEF1B | -2.61387 | 9.53211 | -4.37468 | 2.76E-05 | 0.00101724 | 2.30031563 |
| RANGAP1 | -3.34208 | 16.88745 | -4.37386 | 2.77E-05 | 0.00101724 | 2.29737992 |
| MMP14 | -9.56622 | 34.28186 | -4.36957 | 2.82E-05 | 0.00103058 | 2.28190002 |
| RPH3AL | -1.45339 | 9.357752 | -4.36742 | 2.84E-05 | 0.00103542 | 2.27414487 |
| CRABP2 | -7.13209 | 17.02747 | -4.36512 | 2.86E-05 | 0.00104089 | 2.26585814 |
| MPDU1 | -3.02658 | 15.52616 | -4.36158 | 2.90E-05 | 0.00105149 | 2.2530967 |
| STXBP2 | -5.22376 | 14.33997 | -4.35922 | 2.93E-05 | 0.00105732 | 2.24460154 |
| SH3TC1 | -3.18507 | 12.38176 | -4.35082 | 3.03E-05 | 0.00108163 | 2.21435998 |
| TCEB3B | -1.21908 | 6.600187 | -4.35045 | 3.03E-05 | 0.00108163 | 2.21303979 |
| CST2 | -2.00882 | 9.224698 | -4.35013 | 3.04E-05 | 0.00108163 | 2.21189433 |
| SEMA4G | -3.51968 | 13.5207 | -4.34965 | 3.04E-05 | 0.00108163 | 2.21016876 |
| CD207 | -1.74425 | 7.702794 | -4.34843 | 3.06E-05 | 0.00108287 | 2.20578423 |
| BAX | -5.87326 | 22.05249 | -4.3443 | 3.11E-05 | 0.00109651 | 2.19091016 |
| CXCL14 | 12.62748 | 18.15626 | 4.341068 | 3.15E-05 | 0.00109965 | 2.17932146 |
| STK38 | -10.8929 | 48.8607 | -4.34059 | 3.15E-05 | 0.00109965 | 2.1776071 |
| PGBD4 | -3.37788 | 11.98414 | -4.33968 | 3.16E-05 | 0.00109965 | 2.17434595 |
| CELSR3 | -1.66335 | 9.154787 | -4.33945 | 3.17E-05 | 0.00109965 | 2.17352119 |
| MAOA | 9.671419 | 33.92193 | 4.33899 | 3.17E-05 | 0.00109965 | 2.171862 |
| ADAM7 | -1.07724 | 5.574133 | -4.33554 | 3.21E-05 | 0.00111011 | 2.15946669 |
| NRG1 | -1.51238 | 8.556893 | -4.33469 | 3.22E-05 | 0.00111011 | 2.15644511 |
| EDN3 | -2.14102 | 8.533832 | -4.33292 | 3.25E-05 | 0.00111011 | 2.15008657 |
| TBX19 | -2.49515 | 13.55575 | -4.33235 | 3.25E-05 | 0.00111011 | 2.14805693 |
| GCM2 | -1.98099 | 6.080938 | -4.33206 | 3.26E-05 | 0.00111011 | 2.14700486 |
| YKT6 | -6.55734 | 29.84682 | -4.33032 | 3.28E-05 | 0.00111381 | 2.14074735 |
| KCNS1 | -7.23007 | 30.01874 | -4.32451 | 3.35E-05 | 0.00113526 | 2.11996004 |
| C1QTNF6 | -1.93692 | 9.800902 | -4.3214 | 3.40E-05 | 0.00114512 | 2.10880595 |
| PDPK1 | -2.64058 | 13.52315 | -4.31828 | 3.44E-05 | 0.00115292 | 2.09765572 |
| PAX8 | -1.83158 | 10.87325 | -4.31788 | 3.44E-05 | 0.00115292 | 2.0962403 |
| DEAF1 | -1.26238 | 10.75875 | -4.3157 | 3.47E-05 | 0.00115877 | 2.08843422 |
| TLR6 | -3.0229 | 9.040355 | -4.31013 | 3.55E-05 | 0.00117932 | 2.06851189 |
| PLCB3 | -2.52912 | 20.24983 | -4.30942 | 3.56E-05 | 0.00117932 | 2.06597625 |
| TLR4 | -7.10551 | 17.37932 | -4.30546 | 3.61E-05 | 0.00118849 | 2.05182763 |
| FIBP | -9.92032 | 37.50402 | -4.30482 | 3.62E-05 | 0.00118849 | 2.04955164 |
| SYT14 | -1.10282 | 5.846861 | -4.30094 | 3.68E-05 | 0.00120074 | 2.0356967 |
| AP4M1 | -1.44252 | 11.53895 | -4.29577 | 3.75E-05 | 0.00121862 | 2.01726667 |
| BIN2 | -7.21504 | 14.90386 | -4.29259 | 3.80E-05 | 0.00122958 | 2.00596463 |
| DPP3 | -5.12739 | 20.77481 | -4.28763 | 3.87E-05 | 0.00124927 | 1.9882784 |
| DOK1 | -3.15568 | 18.58826 | -4.28412 | 3.92E-05 | 0.00125897 | 1.97578337 |
| PDGFD | 2.64323 | 11.80101 | 4.283927 | 3.92E-05 | 0.00125897 | 1.97511242 |
| RDH8 | -1.09436 | 7.290202 | -4.28308 | 3.94E-05 | 0.001259 | 1.9720899 |
| SH3GL1 | -3.86175 | 21.74711 | -4.278 | 4.02E-05 | 0.00127974 | 1.95402851 |
| TLR9 | -1.25642 | 7.850849 | -4.27553 | 4.05E-05 | 0.00128744 | 1.94526113 |
| GPC2 | -1.686 | 9.260978 | -4.27409 | 4.08E-05 | 0.00128744 | 1.94013156 |
| TCP10L | -1.36153 | 8.461971 | -4.27366 | 4.08E-05 | 0.00128744 | 1.93860455 |
| MEFV | -4.3396 | 9.149325 | -4.26382 | 4.24E-05 | 0.00132576 | 1.90370242 |
| TEX11 | -1.07126 | 6.118995 | -4.26233 | 4.26E-05 | 0.00132916 | 1.89841848 |
| NMU | -1.21614 | 6.114218 | -4.26128 | 4.28E-05 | 0.0013303 | 1.8947145 |
| F2RL1 | -4.7618 | 11.71493 | -4.25986 | 4.31E-05 | 0.00133337 | 1.88968385 |
| P2RX1 | 10.42723 | 26.08603 | 4.257437 | 4.35E-05 | 0.00134164 | 1.88108851 |
| HIC1 | -1.92868 | 10.25691 | -4.25523 | 4.38E-05 | 0.00134882 | 1.87327791 |
| SLC27A2 | -1.14115 | 6.045533 | -4.25032 | 4.47E-05 | 0.00137021 | 1.85589417 |
| KCNJ6 | -2.2317 | 7.519855 | -4.24863 | 4.50E-05 | 0.00137484 | 1.84992212 |
| CHRDL1 | 13.9305 | 18.52347 | 4.247776 | 4.51E-05 | 0.00137508 | 1.84690184 |
| DOK4 | -4.1548 | 15.357 | -4.24302 | 4.59E-05 | 0.00139606 | 1.83009252 |
| SIGLEC10 | -7.41875 | 14.00581 | -4.23906 | 4.66E-05 | 0.00141305 | 1.81609891 |
| DAO | -1.03377 | 8.225794 | -4.23507 | 4.73E-05 | 0.00143037 | 1.80203835 |
| APBA1 | -1.56394 | 10.17595 | -4.23379 | 4.76E-05 | 0.00143304 | 1.79749338 |
| SLC34A1 | -1.30333 | 7.486868 | -4.23251 | 4.78E-05 | 0.00143568 | 1.79298295 |
| IKBKE | -3.50215 | 14.28191 | -4.22521 | 4.92E-05 | 0.00147182 | 1.76726788 |
| ABCG8 | -1.21174 | 6.331389 | -4.21949 | 5.03E-05 | 0.00149979 | 1.74712405 |
| TUSC3 | -3.99015 | 14.46078 | -4.21126 | 5.19E-05 | 0.00154299 | 1.71817116 |
| CDH4 | -1.27675 | 8.385083 | -4.21015 | 5.21E-05 | 0.00154439 | 1.71427138 |
| LIPF | -1.04843 | 7.325739 | -4.20944 | 5.22E-05 | 0.00154439 | 1.71178545 |
| DMRTC2 | -1.48465 | 7.691396 | -4.20544 | 5.30E-05 | 0.00156344 | 1.69772728 |
| SLC35C2 | -2.23043 | 16.67059 | -4.20401 | 5.33E-05 | 0.0015673 | 1.69270152 |
| CYP27B1 | -1.15902 | 7.143568 | -4.1938 | 5.54E-05 | 0.00161975 | 1.65689652 |
| PTDSS2 | -3.22501 | 18.62435 | -4.18988 | 5.63E-05 | 0.00163917 | 1.6431933 |
| HRC | 4.935079 | 13.01565 | 4.185013 | 5.73E-05 | 0.0016586 | 1.62614497 |
| HRK | -1.78866 | 10.38495 | -4.18446 | 5.74E-05 | 0.0016586 | 1.62420432 |
| OSBPL3 | -3.67542 | 10.85842 | -4.18029 | 5.83E-05 | 0.00167763 | 1.60961569 |
| FCHO1 | -1.76327 | 9.154028 | -4.1789 | 5.87E-05 | 0.00167763 | 1.60478052 |
| GSTZ1 | -1.98947 | 10.25515 | -4.17869 | 5.87E-05 | 0.00167763 | 1.60402937 |
| CNTN1 | 3.734893 | 7.519826 | 4.178383 | 5.88E-05 | 0.00167763 | 1.60296272 |
| GLCE | -3.29378 | 12.50208 | -4.17461 | 5.96E-05 | 0.00169691 | 1.58977386 |
| CLDN14 | -1.43377 | 8.738883 | -4.16865 | 6.10E-05 | 0.00172567 | 1.56899497 |
| SULT1E1 | -7.37802 | 8.643728 | -4.16552 | 6.17E-05 | 0.00174129 | 1.55805712 |
| XRCC3 | -3.05992 | 11.53534 | -4.16106 | 6.28E-05 | 0.00176591 | 1.54250262 |
| NOVA2 | -2.01169 | 11.68332 | -4.15987 | 6.30E-05 | 0.00176881 | 1.53836661 |
| GALNT10 | -4.70551 | 19.37271 | -4.15895 | 6.33E-05 | 0.00176994 | 1.53515787 |
| TNFRSF4 | -1.14798 | 7.998042 | -4.15757 | 6.36E-05 | 0.00177414 | 1.53036566 |
| DIRAS1 | -1.21789 | 7.86548 | -4.15577 | 6.40E-05 | 0.00178125 | 1.52408307 |
| SLC4A2 | -2.87191 | 17.99356 | -4.15064 | 6.53E-05 | 0.00181103 | 1.50622539 |
| CATSPER1 | -1.31933 | 9.001381 | -4.1483 | 6.59E-05 | 0.00182198 | 1.49809221 |
| PRKAR1B | -2.12558 | 11.62045 | -4.14645 | 6.63E-05 | 0.00182957 | 1.49168221 |
| CA9 | -4.78469 | 18.67718 | -4.14092 | 6.77E-05 | 0.00186293 | 1.47248235 |
| CTRL | -1.88585 | 9.327946 | -4.13004 | 7.06E-05 | 0.00193308 | 1.43472866 |
| GDF2 | -3.53682 | 14.71918 | -4.12964 | 7.07E-05 | 0.00193308 | 1.43334899 |
| ANGPTL1 | 6.663847 | 14.0895 | 4.123158 | 7.24E-05 | 0.00197537 | 1.4108915 |
| BTBD11 | -2.18853 | 7.139063 | -4.11115 | 7.58E-05 | 0.00205524 | 1.36936048 |
| NAP1L2 | 5.425043 | 11.60927 | 4.109001 | 7.64E-05 | 0.00206428 | 1.36195093 |
| SMPD2 | -1.37076 | 9.90783 | -4.10849 | 7.65E-05 | 0.00206428 | 1.36016887 |
| REG1B | -1.4054 | 6.163394 | -4.10725 | 7.69E-05 | 0.00206428 | 1.35591744 |
| FSD1 | -2.13139 | 9.79811 | -4.10287 | 7.81E-05 | 0.00209126 | 1.34080628 |
| SDSL | -5.64965 | 17.38409 | -4.10001 | 7.90E-05 | 0.00209721 | 1.33094489 |
| KCNA3 | -3.07897 | 7.54539 | -4.09985 | 7.90E-05 | 0.00209721 | 1.33036813 |
| CACNG2 | -1.16904 | 7.140007 | -4.09962 | 7.91E-05 | 0.00209721 | 1.32958491 |
| TARBP2 | -3.8076 | 18.93614 | -4.09923 | 7.92E-05 | 0.00209721 | 1.32825383 |
| SNAP29 | -3.06945 | 18.71436 | -4.09259 | 8.12E-05 | 0.00214433 | 1.30536061 |
| MYL5 | -2.67231 | 13.41698 | -4.09005 | 8.20E-05 | 0.00215905 | 1.29661244 |
| KLK12 | -1.33084 | 8.249445 | -4.08817 | 8.26E-05 | 0.00215937 | 1.290156 |
| NCR1 | -1.34562 | 7.388306 | -4.08766 | 8.27E-05 | 0.00215937 | 1.2883975 |
| PGM5 | 6.21942 | 14.12388 | 4.087576 | 8.28E-05 | 0.00215937 | 1.2881145 |
| POLD1 | -1.97102 | 11.74534 | -4.08716 | 8.29E-05 | 0.00215937 | 1.28669822 |
| CHAF1B | -1.06534 | 8.059949 | -4.08453 | 8.37E-05 | 0.00217499 | 1.27764495 |
| ADORA2B | -2.98402 | 11.66002 | -4.08332 | 8.41E-05 | 0.00217911 | 1.27348479 |
| DNAH17 | -1.04248 | 5.915882 | -4.0785 | 8.56E-05 | 0.00221294 | 1.25691132 |
| MUC13 | -1.48876 | 7.615121 | -4.07383 | 8.71E-05 | 0.00224172 | 1.2408919 |
| TRAF1 | -2.97298 | 11.95828 | -4.07363 | 8.72E-05 | 0.00224172 | 1.24022348 |
| ADAMTS12 | -1.94675 | 8.921415 | -4.07048 | 8.82E-05 | 0.00226241 | 1.22938635 |
| INA | -2.65003 | 8.561374 | -4.0666 | 8.95E-05 | 0.00228937 | 1.21611427 |
| LIFR | 8.938946 | 14.94488 | 4.065802 | 8.98E-05 | 0.00229027 | 1.21336897 |
| SERPIND1 | -3.10339 | 7.794853 | -4.06341 | 9.06E-05 | 0.00230483 | 1.20517137 |
| TREH | -1.02877 | 6.892058 | -4.05809 | 9.24E-05 | 0.00234495 | 1.18694785 |
| NUDT6 | -1.41914 | 8.779923 | -4.05735 | 9.26E-05 | 0.0023453 | 1.18444489 |
| SCLY | -1.47274 | 10.7108 | -4.05437 | 9.37E-05 | 0.00236043 | 1.17423136 |
| MASP2 | -1.66704 | 8.52543 | -4.05425 | 9.37E-05 | 0.00236043 | 1.17383025 |
| ARSB | -3.66041 | 13.08461 | -4.05308 | 9.41E-05 | 0.00236472 | 1.1698156 |
| ATP8A2 | -1.21265 | 6.316518 | -4.05224 | 9.44E-05 | 0.00236607 | 1.16695144 |
| BNIP1 | -1.41826 | 10.36443 | -4.04259 | 9.79E-05 | 0.0024401 | 1.13399517 |
| PRND | -1.34724 | 6.227442 | -4.04003 | 9.88E-05 | 0.00245714 | 1.12528556 |
| PLA2G2A | 7.867034 | 13.01625 | 4.03356 | 0.000101 | 0.00251057 | 1.10321781 |
| AP4E1 | -1.3362 | 9.025018 | -4.03211 | 0.000102 | 0.0025178 | 1.09826741 |
| SULT4A1 | -2.82661 | 9.017675 | -4.02769 | 0.000103 | 0.00255292 | 1.08324478 |
| EEF1A2 | -1.48475 | 9.06417 | -4.02676 | 0.000104 | 0.0025554 | 1.08005879 |
| PRG3 | 8.754178 | 10.32944 | 4.025893 | 0.000104 | 0.002556 | 1.07712055 |
| POLE2 | -1.50407 | 8.333386 | -4.02535 | 0.000104 | 0.002556 | 1.07526183 |
| DPT | 4.356151 | 10.04735 | 4.0236 | 0.000105 | 0.00256531 | 1.06932171 |
| GCKR | -2.66821 | 10.88775 | -4.02303 | 0.000105 | 0.00256531 | 1.06737196 |
| SIX3 | -1.03065 | 6.54535 | -4.01839 | 0.000107 | 0.0026033 | 1.05160728 |
| ETV5 | -8.86006 | 31.55337 | -4.01446 | 0.000109 | 0.00262847 | 1.0382558 |
| GNG4 | -1.97062 | 8.072902 | -4.01207 | 0.00011 | 0.00264527 | 1.03015888 |
| FGL1 | -1.67958 | 7.766921 | -3.99621 | 0.000116 | 0.00279792 | 0.97643466 |
| IRF4 | -2.17928 | 9.205127 | -3.98669 | 0.00012 | 0.00288468 | 0.94426027 |
| SLC34A3 | -2.24141 | 13.48747 | -3.9866 | 0.00012 | 0.00288468 | 0.94395122 |
| KPTN | -4.45531 | 16.59689 | -3.9859 | 0.000121 | 0.00288514 | 0.94157368 |
| LAIR1 | -11.2797 | 20.34325 | -3.98291 | 0.000122 | 0.00291003 | 0.93147231 |
| SIGLEC6 | -1.16631 | 7.01531 | -3.97542 | 0.000125 | 0.00296766 | 0.90621418 |
| CD209 | -3.10445 | 11.58715 | -3.97495 | 0.000126 | 0.00296766 | 0.90465153 |
| AGTR1 | 1.99349 | 8.778877 | 3.973695 | 0.000126 | 0.00297427 | 0.90041586 |
| RABIF | -3.56065 | 21.28292 | -3.97246 | 0.000127 | 0.00297561 | 0.89624046 |
| CCNE1 | -1.67923 | 9.282337 | -3.97112 | 0.000127 | 0.00297561 | 0.89173927 |
| KCNQ3 | -2.13777 | 9.622248 | -3.97111 | 0.000127 | 0.00297561 | 0.89170541 |
| ZNF154 | -1.46509 | 7.62411 | -3.97049 | 0.000128 | 0.00297561 | 0.88962164 |
| ABCB8 | -1.65822 | 10.94766 | -3.97034 | 0.000128 | 0.00297561 | 0.88910024 |
| PDE6G | -1.71344 | 8.009969 | -3.96848 | 0.000129 | 0.00298891 | 0.88284683 |
| SLC9A7 | -1.73824 | 8.161648 | -3.96464 | 0.000131 | 0.00302419 | 0.86992899 |
| TNFSF15 | -2.28612 | 9.861938 | -3.96151 | 0.000132 | 0.0030499 | 0.85940471 |
| ZNF8 | -1.88271 | 12.81497 | -3.96105 | 0.000132 | 0.0030499 | 0.85786299 |
| AGRP | -2.64802 | 11.58056 | -3.95676 | 0.000134 | 0.0030873 | 0.84344245 |
| ELAVL4 | -1.43208 | 6.749683 | -3.95588 | 0.000135 | 0.0030873 | 0.8405134 |
| HPS6 | -2.64518 | 14.7234 | -3.95581 | 0.000135 | 0.0030873 | 0.84027515 |
| LZTS1 | -1.96668 | 11.10867 | -3.95315 | 0.000136 | 0.00311033 | 0.83133506 |
| PIGN | -1.94516 | 12.31025 | -3.95239 | 0.000137 | 0.0031118 | 0.82877735 |
| ABCC9 | 2.52696 | 8.362792 | 3.950216 | 0.000138 | 0.0031294 | 0.82148811 |
| HM13 | -4.41477 | 22.00942 | -3.94615 | 0.00014 | 0.00316606 | 0.8078563 |
| CDCA2 | -1.21514 | 6.810976 | -3.94577 | 0.00014 | 0.00316606 | 0.8065922 |
| LRRC25 | -7.60131 | 26.80421 | -3.94287 | 0.000141 | 0.00319057 | 0.7968741 |
| B4GALT7 | -3.63163 | 22.01979 | -3.94238 | 0.000142 | 0.00319057 | 0.79522649 |
| TXNRD2 | -1.47355 | 11.39558 | -3.94178 | 0.000142 | 0.00319057 | 0.79322943 |
| ADAM30 | -1.14717 | 6.408091 | -3.9386 | 0.000144 | 0.00322028 | 0.78258081 |
| HDAC10 | -2.01142 | 11.65811 | -3.9377 | 0.000144 | 0.00322028 | 0.7795443 |
| MESDC2 | -8.34068 | 28.32424 | -3.93738 | 0.000144 | 0.00322028 | 0.77848353 |
| CLN3 | -2.13856 | 15.06107 | -3.93596 | 0.000145 | 0.00322973 | 0.77372274 |
| FOXD2 | -1.16265 | 8.22552 | -3.93385 | 0.000146 | 0.00324 | 0.76667837 |
| JAK3 | -3.13387 | 11.7368 | -3.93139 | 0.000147 | 0.00326191 | 0.75844402 |
| GHRH | -1.52566 | 7.386351 | -3.92816 | 0.000149 | 0.00329318 | 0.74764834 |
| HOXB9 | -1.2655 | 7.977431 | -3.92639 | 0.00015 | 0.00330698 | 0.74176685 |
| PNKP | -2.23068 | 13.01497 | -3.92501 | 0.000151 | 0.00331629 | 0.7371463 |
| PQLC2 | -2.02179 | 13.84025 | -3.92276 | 0.000152 | 0.00332879 | 0.72963138 |
| AURKC | -1.19022 | 8.40713 | -3.92276 | 0.000152 | 0.00332879 | 0.72961995 |
| TLX3 | -1.55351 | 7.286581 | -3.91825 | 0.000155 | 0.00337263 | 0.71458821 |
| BUB1B | -3.41089 | 9.263775 | -3.91795 | 0.000155 | 0.00337263 | 0.71357563 |
| IL10 | -2.3748 | 8.565498 | -3.9167 | 0.000155 | 0.00338046 | 0.70942957 |
| IL2RG | -3.70427 | 13.95008 | -3.91502 | 0.000156 | 0.00339144 | 0.70382322 |
| SPATA9 | -1.23534 | 5.93432 | -3.9146 | 0.000157 | 0.00339144 | 0.70242467 |
| DOK2 | -10.0048 | 19.36098 | -3.91207 | 0.000158 | 0.00341191 | 0.69401622 |
| ERMAP | -1.55005 | 11.98866 | -3.91174 | 0.000158 | 0.00341191 | 0.69289974 |
| PPFIBP1 | -6.63645 | 22.50825 | -3.90745 | 0.000161 | 0.00345037 | 0.67863718 |
| GALR3 | -2.48067 | 12.76153 | -3.90591 | 0.000162 | 0.00346223 | 0.67350383 |
| IGFBP1 | -5.25425 | 14.26747 | -3.90076 | 0.000165 | 0.0035139 | 0.65636868 |
| MAD2L2 | -3.30555 | 15.66633 | -3.89936 | 0.000166 | 0.00352262 | 0.65172139 |
| CENPA | -1.51938 | 8.704447 | -3.89597 | 0.000168 | 0.00354705 | 0.64048011 |
| TRIM26 | -3.66644 | 26.86908 | -3.89593 | 0.000168 | 0.00354705 | 0.64032267 |
| FANCA | -1.4139 | 9.480164 | -3.8943 | 0.000169 | 0.00354705 | 0.6349377 |
| PCDH8 | 2.586088 | 7.304749 | 3.894145 | 0.000169 | 0.00354705 | 0.63441245 |
| ALG1 | -1.29904 | 11.02594 | -3.89352 | 0.000169 | 0.00354705 | 0.63232393 |
| PIP5K1B | 3.598067 | 8.717993 | 3.89252 | 0.00017 | 0.00354705 | 0.62902115 |
| PEX14 | -1.68532 | 13.35639 | -3.89214 | 0.00017 | 0.00354705 | 0.62777476 |
| SP7 | -1.58918 | 8.846014 | -3.88851 | 0.000172 | 0.00358641 | 0.61573815 |
| GHRHR | -1.08547 | 6.534466 | -3.88609 | 0.000174 | 0.00360282 | 0.60769967 |
| OAZ3 | -1.11921 | 7.046498 | -3.8816 | 0.000177 | 0.00365412 | 0.59283904 |
| GABRR1 | -1.08259 | 7.144003 | -3.87963 | 0.000178 | 0.00367256 | 0.58631242 |
| REG1A | -1.26289 | 7.173065 | -3.87469 | 0.000181 | 0.00373092 | 0.56997116 |
| KLF8 | 2.642734 | 10.04592 | 3.872934 | 0.000182 | 0.00374684 | 0.56416594 |
| PRKCE | -1.57867 | 9.288676 | -3.86917 | 0.000185 | 0.00379017 | 0.55173842 |
| TRPV4 | -2.35813 | 11.28422 | -3.86713 | 0.000186 | 0.00380377 | 0.54499335 |
| MPZL1 | -4.7711 | 25.61606 | -3.86703 | 0.000186 | 0.00380377 | 0.54465904 |
| PALMD | 2.505459 | 9.426531 | 3.864522 | 0.000188 | 0.00383038 | 0.5363877 |
| ASPA | 9.109655 | 12.18071 | 3.860025 | 0.000191 | 0.00387218 | 0.52155666 |
| SHANK3 | 4.405298 | 11.99414 | 3.859903 | 0.000191 | 0.00387218 | 0.52115392 |
| KIF25 | -1.09371 | 7.224519 | -3.8598 | 0.000191 | 0.00387218 | 0.52079753 |
| CD28 | -4.83192 | 9.052534 | -3.85624 | 0.000194 | 0.0039141 | 0.50907079 |
| MSR1 | -12.5439 | 17.17158 | -3.85255 | 0.000196 | 0.00395346 | 0.49693293 |
| MGAT4B | -6.45792 | 24.36544 | -3.85232 | 0.000196 | 0.00395346 | 0.49617543 |
| GMIP | -4.05555 | 13.37865 | -3.84692 | 0.0002 | 0.00402285 | 0.47839929 |
| BTNL2 | -1.1389 | 6.054296 | -3.84412 | 0.000202 | 0.00405525 | 0.46920231 |
| CHST1 | -1.80328 | 11.43754 | -3.84142 | 0.000204 | 0.00408548 | 0.46033943 |
| TRIP13 | -1.96145 | 8.771123 | -3.8399 | 0.000205 | 0.00409214 | 0.45535519 |
| DPAGT1 | -4.1612 | 21.629 | -3.83277 | 0.000211 | 0.00417927 | 0.43192678 |
| TRAF4 | -1.62249 | 11.29843 | -3.8324 | 0.000211 | 0.00417927 | 0.43074621 |
| SLC9A1 | -4.23116 | 23.01606 | -3.83235 | 0.000211 | 0.00417927 | 0.43056588 |
| GGH | -5.59135 | 16.59925 | -3.83069 | 0.000212 | 0.00419585 | 0.42510861 |
| HS2ST1 | -4.42179 | 14.59611 | -3.82806 | 0.000214 | 0.00422132 | 0.41650629 |
| TNFRSF18 | -1.52958 | 9.901851 | -3.8276 | 0.000215 | 0.00422132 | 0.41498417 |
| EFNA3 | -3.42063 | 14.31915 | -3.827 | 0.000215 | 0.00422132 | 0.41303228 |
| KHDRBS3 | 5.046824 | 20.08293 | 3.825516 | 0.000216 | 0.00422132 | 0.40817233 |
| FBXO16 | 1.899205 | 8.569913 | 3.825041 | 0.000217 | 0.00422132 | 0.40661706 |
| GRINA | -5.81902 | 24.54783 | -3.82468 | 0.000217 | 0.00422132 | 0.4054484 |
| GRK6 | -4.02258 | 19.1619 | -3.82425 | 0.000217 | 0.00422132 | 0.40402095 |
| CLDN7 | -2.34518 | 10.97058 | -3.82423 | 0.000217 | 0.00422132 | 0.4039477 |
| TRAPPC1 | -3.93981 | 23.47756 | -3.824 | 0.000217 | 0.00422132 | 0.40321153 |
| KIF9 | -2.24146 | 12.67303 | -3.82246 | 0.000219 | 0.00423633 | 0.3981676 |
| DSCR4 | -1.19344 | 6.746233 | -3.82008 | 0.00022 | 0.00426421 | 0.3903784 |
| MTNR1B | -1.12913 | 6.873231 | -3.81908 | 0.000221 | 0.00427114 | 0.38710127 |
| AGPAT4 | -4.27091 | 11.97248 | -3.81657 | 0.000223 | 0.00430124 | 0.37889563 |
| POLH | -2.43311 | 14.63562 | -3.81429 | 0.000225 | 0.00432616 | 0.37144163 |
| STK36 | -2.53275 | 16.76637 | -3.81336 | 0.000226 | 0.00432616 | 0.36840325 |
| ARRB2 | -8.36723 | 19.55516 | -3.81278 | 0.000226 | 0.00432616 | 0.36651272 |
| PSG1 | -1.54148 | 8.752924 | -3.81277 | 0.000226 | 0.00432616 | 0.36649537 |
| FBN2 | -1.12758 | 6.58609 | -3.81055 | 0.000228 | 0.00435013 | 0.35923498 |
| CD86 | -8.71987 | 17.53342 | -3.81014 | 0.000228 | 0.00435013 | 0.35790892 |
| P2RX4 | -3.30632 | 12.98426 | -3.80925 | 0.000229 | 0.00435561 | 0.35499283 |
| CCL24 | -1.06496 | 6.773469 | -3.80573 | 0.000232 | 0.00440224 | 0.34350555 |
| ENTPD7 | -1.67609 | 10.57336 | -3.80333 | 0.000234 | 0.00442354 | 0.33568273 |
| CABLES1 | -2.4361 | 12.06866 | -3.8033 | 0.000234 | 0.00442354 | 0.33558461 |
| PANX1 | -7.04828 | 23.81595 | -3.80222 | 0.000235 | 0.00443217 | 0.33205403 |
| SLC22A5 | -3.39612 | 17.92674 | -3.80114 | 0.000236 | 0.00443844 | 0.32855708 |
| LGALS8 | -4.71708 | 20.64828 | -3.80075 | 0.000236 | 0.00443844 | 0.32727092 |
| KLRC3 | -1.00269 | 4.985364 | -3.79777 | 0.000239 | 0.00447732 | 0.31756554 |
| KIFC1 | -4.0854 | 15.84927 | -3.79676 | 0.00024 | 0.00448484 | 0.31429646 |
| INSL6 | -1.0139 | 6.25334 | -3.78911 | 0.000246 | 0.00459987 | 0.28940706 |
| LACRT | -1.2381 | 6.357083 | -3.78859 | 0.000247 | 0.00459987 | 0.28770839 |
| HN1 | -6.29614 | 19.67599 | -3.78805 | 0.000247 | 0.00460003 | 0.2859501 |
| SLC27A4 | -1.61885 | 11.7996 | -3.78717 | 0.000248 | 0.00460574 | 0.28309393 |
| USP28 | -3.22986 | 15.76314 | -3.78611 | 0.000249 | 0.00461441 | 0.27965658 |
| OLR1 | -14.7491 | 20.31726 | -3.78479 | 0.00025 | 0.00462739 | 0.27537426 |
| KCNJ5 | -4.54444 | 12.4946 | -3.78416 | 0.000251 | 0.00462912 | 0.27331925 |
| BMX | 1.919829 | 9.204245 | 3.78307 | 0.000252 | 0.00463843 | 0.26977371 |
| GPR157 | -2.44431 | 12.46692 | -3.78251 | 0.000252 | 0.00463897 | 0.26795892 |
| CPB2 | -1.01466 | 5.836516 | -3.78169 | 0.000253 | 0.00464391 | 0.26528383 |
| ACTA1 | 4.558795 | 15.71704 | 3.780219 | 0.000254 | 0.00465951 | 0.26052046 |
| SMARCD1 | -3.64436 | 21.56169 | -3.77856 | 0.000256 | 0.00466896 | 0.25512801 |
| CHPF | -4.81334 | 29.83805 | -3.77848 | 0.000256 | 0.00466896 | 0.25486338 |
| RAD51 | -1.53985 | 7.99643 | -3.77808 | 0.000256 | 0.00466896 | 0.25358836 |
| STARD5 | -1.44101 | 8.606813 | -3.77725 | 0.000257 | 0.00467416 | 0.25088408 |
| MAS1 | -1.27994 | 7.891962 | -3.77462 | 0.000259 | 0.00470938 | 0.24234766 |
| SLITRK6 | 1.460677 | 6.620095 | 3.771583 | 0.000262 | 0.00475158 | 0.23252352 |
| E2F1 | -2.06743 | 11.55857 | -3.77002 | 0.000263 | 0.00476927 | 0.22745483 |
| PSME3 | -3.71606 | 21.77173 | -3.76864 | 0.000265 | 0.00478136 | 0.22300557 |
| SOAT2 | -1.4567 | 9.318978 | -3.76827 | 0.000265 | 0.00478136 | 0.22179497 |
| TNFRSF19 | -1.57775 | 9.689316 | -3.76764 | 0.000266 | 0.00478335 | 0.21974642 |
| CISH | -2.93094 | 15.07947 | -3.76518 | 0.000268 | 0.00481639 | 0.21179991 |
| NDUFV3 | -3.85038 | 24.09539 | -3.76386 | 0.000269 | 0.00483025 | 0.20751473 |
| TROAP | -2.32156 | 10.89624 | -3.75774 | 0.000275 | 0.00492716 | 0.18773252 |
| SLC23A2 | -2.30955 | 12.45422 | -3.75516 | 0.000278 | 0.00496326 | 0.17941687 |
| LAIR2 | -4.4547 | 18.6731 | -3.75426 | 0.000279 | 0.00497007 | 0.1765128 |
| HOXA6 | -1.69448 | 10.03567 | -3.75225 | 0.000281 | 0.00499657 | 0.17001048 |
| GPR146 | 1.161177 | 8.572561 | 3.742782 | 0.00029 | 0.00513869 | 0.13950034 |
| SLC37A2 | -3.36776 | 13.12301 | -3.73684 | 0.000296 | 0.0052382 | 0.12037365 |
| SLC4A8 | -1.87798 | 9.452113 | -3.73471 | 0.000299 | 0.00526825 | 0.11352496 |
| GPR20 | 3.875639 | 9.212725 | 3.733684 | 0.0003 | 0.00527786 | 0.1102308 |
| OPA3 | -1.5991 | 12.70113 | -3.73176 | 0.000302 | 0.00530427 | 0.10405154 |
| PES1 | -2.29533 | 14.3587 | -3.73076 | 0.000303 | 0.00531348 | 0.10084345 |
| BCL11A | -2.85726 | 9.488943 | -3.73009 | 0.000303 | 0.00531663 | 0.09868007 |
| RHBDL1 | -1.16977 | 8.039827 | -3.72922 | 0.000304 | 0.00532352 | 0.09587906 |
| HMMR | -1.12052 | 6.234191 | -3.72826 | 0.000305 | 0.00533206 | 0.09279928 |
| HS6ST2 | -1.03343 | 5.461944 | -3.72681 | 0.000307 | 0.00534993 | 0.08813623 |
| C1GALT1 | -2.40983 | 10.83639 | -3.72492 | 0.000309 | 0.0053726 | 0.08209299 |
| KDELC1 | -3.06565 | 12.23112 | -3.72397 | 0.00031 | 0.00537514 | 0.07902526 |
| ALG3 | -4.05169 | 20.76067 | -3.72162 | 0.000313 | 0.00540122 | 0.07151661 |
| ASF1B | -1.42222 | 7.824616 | -3.7208 | 0.000313 | 0.00540594 | 0.06888349 |
| ABLIM2 | -1.64354 | 10.7205 | -3.71987 | 0.000315 | 0.00540594 | 0.06590164 |
| MGRN1 | -3.1219 | 23.99877 | -3.71985 | 0.000315 | 0.00540594 | 0.0658298 |
| CD34 | 4.114586 | 13.10654 | 3.718988 | 0.000315 | 0.00541291 | 0.06306661 |
| EFNB3 | -1.28628 | 8.531458 | -3.71729 | 0.000317 | 0.00543579 | 0.05763821 |
| CNTN4 | 2.752396 | 9.08305 | 3.716159 | 0.000319 | 0.00544804 | 0.05400266 |
| ZNF581 | -3.93861 | 23.01914 | -3.71506 | 0.00032 | 0.00545969 | 0.05047525 |
| DRD2 | -1.41938 | 9.337958 | -3.70972 | 0.000326 | 0.00555344 | 0.03338716 |
| HAVCR2 | -10.0664 | 19.98864 | -3.70865 | 0.000327 | 0.00556472 | 0.02996144 |
| CD19 | -1.22858 | 7.98278 | -3.70816 | 0.000328 | 0.00556472 | 0.02839078 |
| C7orf34 | -2.88296 | 10.62293 | -3.7056 | 0.000331 | 0.00560283 | 0.0202368 |
| ZNF205 | -3.00148 | 16.44133 | -3.70523 | 0.000331 | 0.00560283 | 0.01903721 |
| CHST11 | -7.86917 | 23.28063 | -3.70433 | 0.000332 | 0.00561079 | 0.01618006 |
| FGG | -1.01448 | 5.73885 | -3.701 | 0.000336 | 0.00564999 | 0.00552017 |
| SLC38A5 | -2.76807 | 10.17437 | -3.70005 | 0.000337 | 0.00564999 | 0.00250947 |
| CDC20 | -2.85687 | 10.21119 | -3.69985 | 0.000337 | 0.00564999 | 0.00187417 |
| FTSJ1 | -2.72296 | 15.99445 | -3.69981 | 0.000337 | 0.00564999 | 0.00173143 |
| SLC22A7 | -1.00856 | 7.409163 | -3.69943 | 0.000338 | 0.00564999 | 0.00051395 |
| LRP1 | -10.9815 | 63.85526 | -3.69666 | 0.000341 | 0.00569538 | -0.0083189 |
| AMN | -2.00805 | 13.04313 | -3.6955 | 0.000343 | 0.0056978 | -0.012021 |
| MRPL23 | -4.538 | 26.85529 | -3.69517 | 0.000343 | 0.0056978 | -0.0130807 |
| AGER | -3.47292 | 14.2586 | -3.69473 | 0.000344 | 0.0056978 | -0.014476 |
| DCPS | -1.71916 | 10.91372 | -3.69461 | 0.000344 | 0.0056978 | -0.0148585 |
| APCS | -1.61736 | 8.536542 | -3.69296 | 0.000346 | 0.00572121 | -0.0201246 |
| NKX2-2 | -4.11654 | 6.659876 | -3.68712 | 0.000353 | 0.0058294 | -0.0387118 |
| XPO5 | -2.51251 | 15.85237 | -3.68464 | 0.000356 | 0.00587035 | -0.0466108 |
| EIF4EBP1 | -4.12556 | 20.49903 | -3.68091 | 0.000361 | 0.00593741 | -0.0584755 |
| HTR2C | -1.67646 | 6.593792 | -3.68028 | 0.000361 | 0.00594052 | -0.0604736 |
| TFF2 | -1.86404 | 9.592923 | -3.67798 | 0.000364 | 0.00596852 | -0.0677876 |
| ARSE | -1.72299 | 9.236476 | -3.67724 | 0.000365 | 0.00597407 | -0.0701467 |
| NAALAD2 | 1.01577 | 6.327517 | 3.676314 | 0.000366 | 0.0059834 | -0.0730784 |
| EXO1 | -1.22019 | 7.138686 | -3.67033 | 0.000374 | 0.00609946 | -0.0920711 |
| SERPINF2 | -2.11304 | 9.333171 | -3.66958 | 0.000375 | 0.00610527 | -0.0944437 |
| C9orf16 | -1.67336 | 13.29648 | -3.66722 | 0.000378 | 0.00614552 | -0.1019253 |
| PSPN | -2.18015 | 10.44396 | -3.66587 | 0.00038 | 0.00615431 | -0.1062243 |
| TRIM14 | -3.87065 | 11.99083 | -3.66327 | 0.000383 | 0.00619153 | -0.1144486 |
| DGAT1 | -2.60578 | 16.96727 | -3.66319 | 0.000384 | 0.00619153 | -0.1146989 |
| TCP10 | -1.21478 | 9.055877 | -3.66156 | 0.000386 | 0.00621568 | -0.1198775 |
| DOK5 | -13.1992 | 26.10407 | -3.66066 | 0.000387 | 0.00621568 | -0.1227047 |
| PSTPIP1 | -3.43659 | 12.47038 | -3.65862 | 0.00039 | 0.00624982 | -0.1291713 |
| EFHC1 | -3.28442 | 14.70983 | -3.6564 | 0.000393 | 0.0062854 | -0.1361911 |
| YARS | -6.95751 | 36.45672 | -3.65605 | 0.000393 | 0.0062854 | -0.137294 |
| MYO7A | -2.18194 | 11.05349 | -3.65145 | 0.000399 | 0.00637644 | -0.15185 |
| GPR31 | -1.76048 | 7.425814 | -3.65095 | 0.0004 | 0.00637714 | -0.153423 |
| PLXNB2 | -8.86877 | 36.80239 | -3.64542 | 0.000408 | 0.00649028 | -0.1708868 |
| ABCB5 | -2.67789 | 7.828558 | -3.64271 | 0.000412 | 0.00651721 | -0.1794471 |
| TTL | -3.47846 | 21.30344 | -3.64259 | 0.000412 | 0.00651721 | -0.1798223 |
| MARK1 | 5.083813 | 13.79218 | 3.642368 | 0.000412 | 0.00651721 | -0.1805107 |
| SLC2A9 | -1.62872 | 10.29153 | -3.63984 | 0.000416 | 0.00655361 | -0.1884885 |
| ARMC3 | -1.09865 | 5.176123 | -3.63811 | 0.000418 | 0.00657841 | -0.1939252 |
| KCNA1 | -1.29971 | 7.816392 | -3.63783 | 0.000419 | 0.00657841 | -0.194828 |
| PBX4 | -2.97007 | 10.23385 | -3.63622 | 0.000421 | 0.00660449 | -0.199874 |
| SLC39A8 | -3.01818 | 11.33581 | -3.63359 | 0.000425 | 0.00661744 | -0.2081525 |
| GTF2IRD1 | -1.86919 | 12.66425 | -3.63349 | 0.000425 | 0.00661744 | -0.2084751 |
| ZNF142 | -1.5195 | 12.32211 | -3.63294 | 0.000426 | 0.00661744 | -0.210218 |
| MOV10L1 | -1.24659 | 7.728356 | -3.63257 | 0.000427 | 0.00661744 | -0.211367 |
| PPFIA2 | 2.801815 | 8.728033 | 3.632456 | 0.000427 | 0.00661744 | -0.2117333 |
| CA10 | -1.4034 | 6.192263 | -3.63171 | 0.000428 | 0.00662418 | -0.2140892 |
| MAL2 | -1.18236 | 6.522003 | -3.62582 | 0.000437 | 0.00672865 | -0.2325904 |
| FXR2 | -2.86144 | 24.83432 | -3.62248 | 0.000442 | 0.00678993 | -0.2430952 |
| SMTN | 12.86755 | 44.38091 | 3.622295 | 0.000442 | 0.00678993 | -0.2436705 |
| SF3B3 | -3.37214 | 27.23433 | -3.61926 | 0.000447 | 0.00685071 | -0.2531862 |
| HOXA7 | -1.13332 | 8.399701 | -3.61806 | 0.000448 | 0.00686845 | -0.25695 |
| CREB3L4 | -1.62638 | 10.07848 | -3.61759 | 0.000449 | 0.00686905 | -0.2584409 |
| RPS6KA4 | -3.49754 | 16.85679 | -3.61688 | 0.00045 | 0.00687524 | -0.2606697 |
| DGKZ | -1.68158 | 10.63919 | -3.61538 | 0.000453 | 0.00689685 | -0.2653519 |
| SIGLEC11 | -1.13057 | 6.78361 | -3.61507 | 0.000453 | 0.00689685 | -0.2663344 |
| FABP4 | 7.993148 | 13.92992 | 3.613964 | 0.000455 | 0.00691257 | -0.2698069 |
| SPI1 | -4.92033 | 16.05498 | -3.61121 | 0.000459 | 0.0069677 | -0.2784243 |
| MED25 | -3.01376 | 16.43964 | -3.60719 | 0.000466 | 0.00705416 | -0.2910272 |
| COLEC11 | -3.54928 | 13.74389 | -3.60482 | 0.000469 | 0.00710094 | -0.2984279 |
| COG2 | -3.10965 | 17.39016 | -3.60428 | 0.00047 | 0.00710325 | -0.3001174 |
| RET | -1.28799 | 9.23147 | -3.6032 | 0.000472 | 0.00711672 | -0.303493 |
| CYYR1 | 3.674366 | 12.47376 | 3.602842 | 0.000473 | 0.00711672 | -0.3046207 |
| XCL1 | -1.08762 | 5.521449 | -3.59823 | 0.00048 | 0.00720842 | -0.3190208 |
| UNC5B | -9.12313 | 31.29343 | -3.59689 | 0.000482 | 0.00722734 | -0.3232039 |
| SEMA6A | 5.557785 | 9.634387 | 3.594259 | 0.000487 | 0.00726849 | -0.331434 |
| MUC4 | -1.07433 | 7.525069 | -3.59405 | 0.000487 | 0.00726849 | -0.332078 |
| WFDC12 | -2.4778 | 10.05288 | -3.58853 | 0.000496 | 0.00739655 | -0.3493108 |
| DECR2 | -3.64327 | 23.59514 | -3.58706 | 0.000499 | 0.00742256 | -0.3538705 |
| MAP2K4 | -2.56206 | 15.32853 | -3.57956 | 0.000512 | 0.00758238 | -0.377244 |
| ANGPTL6 | -1.59529 | 8.447296 | -3.57953 | 0.000512 | 0.00758238 | -0.3773138 |
| FOXA2 | -1.19311 | 7.512812 | -3.57884 | 0.000513 | 0.0075891 | -0.3794818 |
| PFAS | -4.50422 | 20.33619 | -3.57838 | 0.000514 | 0.00758948 | -0.380889 |
| ART5 | -1.79077 | 9.033084 | -3.5757 | 0.000519 | 0.0076319 | -0.389226 |
| SOCS1 | -1.48027 | 10.02749 | -3.57545 | 0.000519 | 0.0076319 | -0.3900204 |
| TDP1 | -1.43189 | 10.16811 | -3.57209 | 0.000525 | 0.00769281 | -0.4004506 |
| EPHA1 | -1.49276 | 9.020813 | -3.57171 | 0.000526 | 0.00769281 | -0.4016363 |
| FGF8 | -2.13986 | 8.040014 | -3.57167 | 0.000526 | 0.00769281 | -0.4017433 |
| FBXO6 | -1.80932 | 10.27444 | -3.57139 | 0.000526 | 0.00769281 | -0.4026368 |
| KCNS2 | -1.23936 | 8.215657 | -3.56961 | 0.00053 | 0.00772835 | -0.4081644 |
| TBX21 | -1.64623 | 9.355901 | -3.56516 | 0.000538 | 0.00783503 | -0.421947 |
| COL22A1 | -1.01682 | 8.509892 | -3.56384 | 0.00054 | 0.00785381 | -0.4260491 |
| MPI | -1.40192 | 12.41634 | -3.5636 | 0.000541 | 0.00785381 | -0.426799 |
| APOBEC3B | -1.64673 | 7.352377 | -3.55679 | 0.000553 | 0.00801478 | -0.4478755 |
| FKBP14 | -8.4126 | 27.97647 | -3.55571 | 0.000555 | 0.00803258 | -0.4512202 |
| VTI1A | -1.86798 | 12.63525 | -3.55256 | 0.000561 | 0.00809854 | -0.4609653 |
| PARVG | -5.32051 | 15.89506 | -3.55244 | 0.000562 | 0.00809854 | -0.4613329 |
| MBD6 | -2.79781 | 14.24447 | -3.55203 | 0.000562 | 0.00809854 | -0.4626242 |
| DMRTB1 | -1.10576 | 6.934396 | -3.5494 | 0.000568 | 0.00815946 | -0.4707453 |
| HELLS | -1.05421 | 7.162013 | -3.54726 | 0.000572 | 0.00820724 | -0.4773638 |
| STX10 | -4.2198 | 27.10932 | -3.54427 | 0.000578 | 0.00827162 | -0.4865816 |
| PSORS1C2 | -1.57342 | 9.687143 | -3.54407 | 0.000578 | 0.00827162 | -0.4871887 |
| SPATA5L1 | -1.86381 | 12.49209 | -3.54368 | 0.000579 | 0.00827162 | -0.4884049 |
| ADCY7 | -4.33135 | 14.75118 | -3.54217 | 0.000582 | 0.00829956 | -0.4930574 |
| LHFPL3 | -5.79184 | 8.005416 | -3.54086 | 0.000584 | 0.00829956 | -0.4971215 |
| CCL26 | -1.85396 | 10.27481 | -3.54063 | 0.000585 | 0.00829956 | -0.4978259 |
| ARG2 | -1.45308 | 10.18946 | -3.54056 | 0.000585 | 0.00829956 | -0.4980185 |
| ZFP64 | -2.87392 | 14.65932 | -3.53837 | 0.000589 | 0.00834963 | -0.5047765 |
| PPP2R1B | -2.28427 | 15.88651 | -3.53532 | 0.000595 | 0.00841258 | -0.5141891 |
| ITIH2 | -8.81115 | 14.71208 | -3.53355 | 0.000599 | 0.008451 | -0.5196191 |
| KRTAP17-1 | -1.35621 | 8.568673 | -3.53175 | 0.000603 | 0.00847859 | -0.5251675 |
| SLC41A2 | -2.46203 | 11.16391 | -3.53045 | 0.000605 | 0.00850396 | -0.5291687 |
| KLF16 | -3.27313 | 18.79341 | -3.52888 | 0.000609 | 0.00853729 | -0.5340064 |
| NTSR1 | -1.43216 | 7.642238 | -3.52834 | 0.00061 | 0.00854068 | -0.5356556 |
| ITGA7 | 13.12599 | 30.28066 | 3.527515 | 0.000611 | 0.00855257 | -0.5382044 |
| SLCO1A2 | -1.266 | 6.116113 | -3.52579 | 0.000615 | 0.00859071 | -0.5435232 |
| ABCG4 | -1.42429 | 9.174483 | -3.52515 | 0.000616 | 0.00859702 | -0.5454733 |
| ATP6V0C | -14.2995 | 103.799 | -3.52456 | 0.000618 | 0.00860196 | -0.5472758 |
| KLRG1 | -1.34743 | 7.999167 | -3.52253 | 0.000622 | 0.00863708 | -0.5535277 |
| TP53I11 | -2.10392 | 13.531 | -3.52157 | 0.000624 | 0.00864163 | -0.5564844 |
| PIGW | -1.48389 | 8.367132 | -3.52154 | 0.000624 | 0.00864163 | -0.5565571 |
| EPHX2 | 1.641479 | 9.462349 | 3.518491 | 0.00063 | 0.00870395 | -0.5659255 |
| SPINT2 | -5.74151 | 26.24158 | -3.51828 | 0.000631 | 0.00870395 | -0.5665892 |
| CCL27 | -1.3467 | 8.889439 | -3.51801 | 0.000631 | 0.00870395 | -0.5673989 |
| SMOC1 | -4.01744 | 13.65411 | -3.51494 | 0.000638 | 0.00876836 | -0.5768053 |
| BCHE | 5.863112 | 7.677302 | 3.514764 | 0.000638 | 0.00876836 | -0.5773606 |
| KCNQ1 | -3.84255 | 17.8321 | -3.50789 | 0.000653 | 0.00894971 | -0.5984201 |
| SQLE | -3.4317 | 13.30306 | -3.50789 | 0.000653 | 0.00894971 | -0.5984256 |
| INHBB | 3.393201 | 10.73728 | 3.504664 | 0.000661 | 0.00902265 | -0.6082923 |
| TACR3 | -1.11466 | 5.758797 | -3.50387 | 0.000662 | 0.00903449 | -0.6107354 |
| BCKDK | -2.73908 | 15.37183 | -3.50336 | 0.000663 | 0.00903733 | -0.6122737 |
| ZDHHC12 | -3.80368 | 20.30845 | -3.50231 | 0.000666 | 0.00905703 | -0.6154965 |
| TNFRSF25 | -3.4226 | 17.41681 | -3.49989 | 0.000671 | 0.00911418 | -0.6229042 |
| FXYD5 | -8.41115 | 33.77207 | -3.49845 | 0.000675 | 0.00913783 | -0.6272824 |
| MEN1 | -2.13772 | 13.52713 | -3.49749 | 0.000677 | 0.00915496 | -0.6302231 |
| NMNAT1 | -2.78698 | 19.9103 | -3.49707 | 0.000678 | 0.00915548 | -0.631517 |
| NFKBIE | -4.35165 | 15.10248 | -3.49548 | 0.000681 | 0.00916571 | -0.6363698 |
| CHAF1A | -1.57647 | 10.71267 | -3.49538 | 0.000682 | 0.00916571 | -0.6366777 |
| EPHA3 | 3.673826 | 9.74872 | 3.495096 | 0.000682 | 0.00916571 | -0.6375349 |
| TNFRSF21 | -11.6443 | 23.8179 | -3.49466 | 0.000683 | 0.00916571 | -0.638857 |
| GORASP1 | -2.40603 | 16.45587 | -3.49443 | 0.000684 | 0.00916571 | -0.6395695 |
| CPNE8 | -2.43522 | 16.99828 | -3.4943 | 0.000684 | 0.00916571 | -0.6399496 |
| BANK1 | -1.17202 | 8.681726 | -3.49355 | 0.000686 | 0.00917645 | -0.6422405 |
| KCNN4 | -5.39186 | 14.297 | -3.49241 | 0.000688 | 0.00919528 | -0.645738 |
| SIL1 | -4.54337 | 31.22439 | -3.49199 | 0.000689 | 0.00919528 | -0.6470081 |
| RNASEL | -4.541 | 19.40708 | -3.49174 | 0.00069 | 0.00919528 | -0.6477781 |
| TRPC4AP | -2.45399 | 20.72356 | -3.49073 | 0.000692 | 0.00921416 | -0.6508591 |
| C1QTNF2 | 5.520429 | 12.14537 | 3.490051 | 0.000694 | 0.00922276 | -0.6529258 |
| CD5 | -2.7506 | 12.66896 | -3.48773 | 0.000699 | 0.00928271 | -0.6600105 |
| CDCA4 | -2.43605 | 18.68489 | -3.4851 | 0.000706 | 0.00932747 | -0.6680159 |
| SYNE2 | 2.354646 | 10.24341 | 3.481181 | 0.000715 | 0.00943857 | -0.6799456 |
| MAGEA12 | -1.23987 | 6.083131 | -3.47996 | 0.000718 | 0.00945196 | -0.6836509 |
| TAL2 | -1.63448 | 8.947398 | -3.4789 | 0.00072 | 0.00947318 | -0.6868893 |
| CD8A | -5.98589 | 15.1123 | -3.47578 | 0.000728 | 0.00956017 | -0.6963672 |
| PIP5K1A | -3.88909 | 22.14192 | -3.47405 | 0.000732 | 0.00960314 | -0.7016296 |
| RAVER1 | -1.6479 | 12.11203 | -3.47307 | 0.000735 | 0.00961927 | -0.7045911 |
| COMMD9 | -6.11235 | 25.37468 | -3.47276 | 0.000736 | 0.00961927 | -0.7055539 |
| SLC22A18 | -3.91471 | 19.35973 | -3.47169 | 0.000738 | 0.00962947 | -0.7088091 |
| PTCRA | -3.36189 | 16.67237 | -3.47165 | 0.000738 | 0.00962947 | -0.7089131 |
| FCN3 | -1.50245 | 9.772551 | -3.47094 | 0.00074 | 0.00963978 | -0.7110776 |
| GTSE1 | -2.87648 | 14.98979 | -3.46664 | 0.000751 | 0.00974114 | -0.7241143 |
| ICOS | -1.9361 | 6.918611 | -3.46664 | 0.000751 | 0.00974114 | -0.7241218 |
| SPACA1 | -1.05598 | 6.460994 | -3.46215 | 0.000762 | 0.00987585 | -0.7377343 |
| ALDH3B1 | -2.7975 | 16.42318 | -3.45739 | 0.000774 | 0.01002118 | -0.7521337 |
| GRIN2D | -1.30602 | 9.21406 | -3.45161 | 0.00079 | 0.01019026 | -0.7696333 |
| TLR1 | -4.50803 | 12.40651 | -3.45058 | 0.000792 | 0.01020653 | -0.7727345 |
| GSG1 | -2.03737 | 6.864335 | -3.45035 | 0.000793 | 0.01020653 | -0.7734423 |
| F8 | 3.081098 | 12.68292 | 3.448146 | 0.000799 | 0.01025506 | -0.780091 |
| DCDC2 | -1.12681 | 5.694609 | -3.44583 | 0.000805 | 0.01032123 | -0.7870819 |
| ZNF100 | -1.5749 | 11.74506 | -3.44327 | 0.000812 | 0.01039641 | -0.7948177 |
| BIRC5 | -2.11047 | 10.11245 | -3.44213 | 0.000815 | 0.01041755 | -0.7982571 |
| SLCO3A1 | -7.98697 | 26.22398 | -3.44188 | 0.000816 | 0.01041755 | -0.799001 |
| STARD3 | -2.72067 | 16.6659 | -3.4393 | 0.000823 | 0.01048037 | -0.8067755 |
| FAH | -3.41494 | 13.40177 | -3.43823 | 0.000826 | 0.01050424 | -0.809999 |
| EPB41L5 | 3.384253 | 10.59112 | 3.436411 | 0.000831 | 0.01055454 | -0.8154831 |
| SRD5A1 | -3.34992 | 15.03877 | -3.43399 | 0.000837 | 0.01062084 | -0.822757 |
| CDK5 | -2.68532 | 14.01988 | -3.43354 | 0.000839 | 0.01062084 | -0.8241383 |
| TFF1 | -2.01002 | 8.950422 | -3.4333 | 0.000839 | 0.01062084 | -0.8248387 |
| CST9L | -1.11527 | 5.658243 | -3.43291 | 0.00084 | 0.01062084 | -0.8260103 |
| ASXL2 | -2.15075 | 16.07416 | -3.43251 | 0.000841 | 0.01062084 | -0.8272175 |
| TAS1R1 | -1.10087 | 7.301352 | -3.43222 | 0.000842 | 0.01062084 | -0.8281083 |
| BTK | -6.06643 | 15.52365 | -3.43105 | 0.000846 | 0.0106485 | -0.8316144 |
| HMX1 | -2.99746 | 13.42701 | -3.4281 | 0.000854 | 0.01071494 | -0.8404962 |
| PPIL1 | -3.50643 | 19.4602 | -3.42798 | 0.000854 | 0.01071494 | -0.8408629 |
| HFE | -1.36248 | 10.63843 | -3.42641 | 0.000859 | 0.01073194 | -0.8455702 |
| TNFRSF9 | -1.19959 | 7.14414 | -3.4234 | 0.000867 | 0.01082629 | -0.8546215 |
| EYA3 | -3.6551 | 16.7216 | -3.42294 | 0.000869 | 0.01082916 | -0.8560047 |
| PLCD3 | -2.27929 | 14.9786 | -3.4196 | 0.000878 | 0.01092928 | -0.8660286 |
| MRPS34 | -4.18711 | 23.46156 | -3.4194 | 0.000879 | 0.01092928 | -0.8665995 |
| SLAMF7 | -5.04125 | 9.907048 | -3.4183 | 0.000882 | 0.01095572 | -0.8699212 |
| MAP3K14 | -1.53314 | 11.12268 | -3.41773 | 0.000884 | 0.01096263 | -0.8716283 |
| PLA1A | -3.31229 | 10.14644 | -3.41664 | 0.000887 | 0.01098857 | -0.8748991 |
| ATF7IP2 | -1.82785 | 13.29333 | -3.41573 | 0.00089 | 0.01100773 | -0.8776072 |
| MAP3K12 | -2.74196 | 26.12242 | -3.41505 | 0.000892 | 0.01101905 | -0.8796688 |
| TRPS1 | -6.55054 | 42.46946 | -3.41039 | 0.000906 | 0.0111765 | -0.8936111 |
| PIK3R2 | -2.26377 | 15.66241 | -3.40975 | 0.000908 | 0.01118612 | -0.8955177 |
| RGS14 | -1.27941 | 9.076373 | -3.40855 | 0.000911 | 0.01121451 | -0.8991163 |
| ABCC3 | -5.35922 | 14.42821 | -3.40824 | 0.000912 | 0.01121451 | -0.9000628 |
| STARD9 | 3.534663 | 13.62755 | 3.407225 | 0.000915 | 0.01123808 | -0.9030845 |
| P2RX5 | -3.229 | 11.63745 | -3.40578 | 0.00092 | 0.01127794 | -0.9074053 |
| TAF6L | -1.50178 | 10.08756 | -3.40263 | 0.000929 | 0.01136774 | -0.9168091 |
| SH3BGR | 8.888873 | 24.00207 | 3.402095 | 0.000931 | 0.01137392 | -0.9184205 |
| ACHE | -1.69502 | 10.00041 | -3.40113 | 0.000934 | 0.01139628 | -0.9213118 |
| BSND | -1.09295 | 7.538281 | -3.39738 | 0.000946 | 0.01152406 | -0.9324918 |
| PRB1 | -4.66368 | 20.49178 | -3.39391 | 0.000956 | 0.01164034 | -0.9428568 |
| ITIH3 | 12.67697 | 32.92865 | 3.393598 | 0.000957 | 0.01164034 | -0.9437815 |
| NCKIPSD | -2.836 | 18.47981 | -3.39048 | 0.000967 | 0.01173729 | -0.9530804 |
| RDH12 | -1.10146 | 6.580019 | -3.39009 | 0.000969 | 0.01173729 | -0.9542342 |
| ANK2 | 6.202821 | 11.18276 | 3.389968 | 0.000969 | 0.01173729 | -0.9545995 |
| SLC39A4 | -1.85416 | 13.13925 | -3.38919 | 0.000971 | 0.01175301 | -0.9569159 |
| OCLM | -1.17581 | 7.49982 | -3.38877 | 0.000973 | 0.01175498 | -0.9581746 |
| LY86 | -16.3897 | 31.46429 | -3.38622 | 0.000981 | 0.01182535 | -0.9657702 |
| SLC25A14 | -5.06376 | 23.58304 | -3.38531 | 0.000984 | 0.01184615 | -0.9684584 |
| DPF1 | -2.52076 | 11.94689 | -3.38467 | 0.000986 | 0.01185669 | -0.9703625 |
| PPP2R3A | 2.689609 | 14.5091 | 3.383548 | 0.00099 | 0.01188626 | -0.9737098 |
| NTNG2 | -3.14915 | 10.61684 | -3.38288 | 0.000992 | 0.0118981 | -0.9757072 |
| GPR55 | -1.26134 | 7.36843 | -3.38115 | 0.000998 | 0.01195154 | -0.9808469 |
| NKX6-1 | -1.18338 | 7.668396 | -3.37787 | 0.001008 | 0.01206648 | -0.9905748 |
| SEMA4F | -1.283 | 12.01157 | -3.37655 | 0.001013 | 0.01209582 | -0.9945214 |
| TECTB | -1.46618 | 8.080831 | -3.37633 | 0.001014 | 0.01209582 | -0.9951507 |
| DGCR6L | -9.16912 | 39.2369 | -3.37561 | 0.001016 | 0.01209582 | -0.9973084 |
| CCKBR | -1.13216 | 7.140762 | -3.3753 | 0.001017 | 0.01209582 | -0.9982159 |
| NEU1 | -3.08572 | 24.65231 | -3.37493 | 0.001018 | 0.01209582 | -0.9993153 |
| MR1 | -3.84523 | 17.88039 | -3.37454 | 0.00102 | 0.01209683 | -1.0004773 |
| ZNF319 | -2.82975 | 16.11825 | -3.37134 | 0.00103 | 0.01219555 | -1.0099752 |
| RASL10B | -2.91894 | 14.87345 | -3.3703 | 0.001034 | 0.01222274 | -1.0130661 |
| SCGB3A2 | -1.55722 | 6.732364 | -3.36803 | 0.001042 | 0.01228837 | -1.0197883 |
| TIGD6 | -1.27246 | 7.080974 | -3.36794 | 0.001042 | 0.01228837 | -1.0200556 |
| PDE6B | -1.01135 | 7.962929 | -3.36476 | 0.001053 | 0.01239593 | -1.0294575 |
| ART1 | -1.17432 | 7.937294 | -3.36456 | 0.001054 | 0.01239593 | -1.0300676 |
| RELB | -1.83264 | 12.27465 | -3.36388 | 0.001056 | 0.01240865 | -1.0320683 |
| PLAG1 | -1.05909 | 8.083682 | -3.36103 | 0.001066 | 0.0124957 | -1.0405176 |
| SLC5A5 | -1.29294 | 9.107805 | -3.35607 | 0.001083 | 0.01267032 | -1.0551665 |
| ARSA | -1.57655 | 11.14434 | -3.35507 | 0.001087 | 0.01269716 | -1.0581424 |
| AKR7A3 | -1.68224 | 13.25918 | -3.35433 | 0.00109 | 0.0126979 | -1.0603303 |
| ASL | -2.66103 | 17.90954 | -3.3537 | 0.001092 | 0.01270888 | -1.0621747 |
| ADAM17 | -8.77593 | 48.23491 | -3.35271 | 0.001095 | 0.01273512 | -1.0650974 |
| QPCT | -3.87754 | 14.09831 | -3.35222 | 0.001097 | 0.0127404 | -1.0665338 |
| AMMECR1 | -2.07001 | 11.21835 | -3.34877 | 0.00111 | 0.01287016 | -1.0767248 |
| TACC3 | -2.9277 | 11.22113 | -3.34364 | 0.001128 | 0.01305749 | -1.0918605 |
| LENEP | -1.61936 | 11.39862 | -3.34153 | 0.001136 | 0.01313253 | -1.0980793 |
| RHO | -1.64719 | 11.53286 | -3.34094 | 0.001138 | 0.01314237 | -1.0998107 |
| CD3G | -3.31024 | 10.01732 | -3.34031 | 0.001141 | 0.01315387 | -1.1016532 |
| SCN2B | -1.15165 | 8.018953 | -3.33945 | 0.001144 | 0.01317539 | -1.1041797 |
| NEUROD2 | -1.54852 | 9.014958 | -3.33695 | 0.001153 | 0.01326795 | -1.1115363 |
| RAB5B | 3.875345 | 26.73177 | 3.335924 | 0.001157 | 0.01329711 | -1.1145645 |
| SEC61A2 | -1.17112 | 9.283073 | -3.32955 | 0.001181 | 0.0135482 | -1.1332833 |
| SPINT1 | -1.35724 | 9.266725 | -3.32948 | 0.001182 | 0.0135482 | -1.133505 |
| ORMDL3 | -3.85626 | 23.89982 | -3.32847 | 0.001186 | 0.01357696 | -1.1364612 |
| SLC6A9 | -1.04278 | 8.342825 | -3.32743 | 0.00119 | 0.0136074 | -1.1395235 |
| ADAMTS2 | -10.8996 | 22.73226 | -3.32565 | 0.001197 | 0.01367074 | -1.1447482 |
| OAS2 | -3.1061 | 12.93017 | -3.32261 | 0.001209 | 0.01377483 | -1.1536614 |
| ADCK5 | -1.30618 | 9.548287 | -3.32202 | 0.001211 | 0.01377535 | -1.1553696 |
| NRL | -1.02466 | 7.663319 | -3.32151 | 0.001213 | 0.01377535 | -1.1568654 |
| SLC25A13 | -2.74726 | 18.4195 | -3.32146 | 0.001213 | 0.01377535 | -1.1570099 |
| TNFRSF11A | -2.10005 | 9.467404 | -3.32118 | 0.001214 | 0.01377535 | -1.1578518 |
| HAGHL | -2.83254 | 15.04929 | -3.32037 | 0.001217 | 0.01379569 | -1.1602163 |
| FSCN2 | -1.0722 | 7.367381 | -3.3187 | 0.001224 | 0.01385486 | -1.1651027 |
| AQP10 | -1.58191 | 7.359574 | -3.31651 | 0.001233 | 0.01393793 | -1.1715148 |
| OR2S2 | -1.23779 | 7.504792 | -3.31067 | 0.001256 | 0.01412655 | -1.188598 |
| DPF2 | 9.983146 | 40.57715 | 3.310615 | 0.001257 | 0.01412655 | -1.1887586 |
| MKI67 | -1.56925 | 8.325095 | -3.309 | 0.001263 | 0.01418469 | -1.1934799 |
| BFSP2 | -1.02866 | 6.769647 | -3.30717 | 0.001271 | 0.01421735 | -1.198832 |
| TBL1X | 9.741599 | 33.30506 | 3.306536 | 0.001273 | 0.01421735 | -1.2006724 |
| RORB | 1.700188 | 9.353742 | 3.306311 | 0.001274 | 0.01421735 | -1.2013311 |
| TNC | 23.53096 | 44.76066 | 3.306302 | 0.001274 | 0.01421735 | -1.2013577 |
| KCTD13 | -1.38685 | 11.79304 | -3.30619 | 0.001275 | 0.01421735 | -1.2016817 |
| CCDC9 | -4.37859 | 24.78831 | -3.30558 | 0.001277 | 0.01422935 | -1.2034583 |
| ART3 | 2.455066 | 7.962561 | 3.302515 | 0.00129 | 0.01434462 | -1.2124087 |
| ADAMTS7 | -2.22737 | 15.79373 | -3.3024 | 0.001291 | 0.01434462 | -1.2127473 |
| NCDN | -1.61239 | 11.71403 | -3.29878 | 0.001306 | 0.01448109 | -1.2232895 |
| SLMAP | 20.27872 | 39.64465 | 3.298033 | 0.001309 | 0.01449997 | -1.225473 |
| ITGA2 | -5.90766 | 12.51382 | -3.29597 | 0.001318 | 0.01456428 | -1.2314724 |
| KRTAP9-4 | -1.08814 | 6.789989 | -3.29207 | 0.001334 | 0.01473277 | -1.2428219 |
| XRCC1 | -1.72298 | 15.91108 | -3.29165 | 0.001336 | 0.01473632 | -1.2440457 |
| DSC2 | -5.94929 | 13.55824 | -3.28999 | 0.001343 | 0.01479933 | -1.2488874 |
| CALB1 | -1.13908 | 6.029489 | -3.28935 | 0.001346 | 0.01481318 | -1.2507333 |
| SCG2 | -8.27147 | 12.10209 | -3.28826 | 0.001351 | 0.01484914 | -1.2539164 |
| PPFIA3 | -1.83159 | 12.28695 | -3.28772 | 0.001353 | 0.0148583 | -1.2554735 |
| AVPR1A | 9.594399 | 13.43479 | 3.28722 | 0.001356 | 0.01486589 | -1.256934 |
| REPS2 | -2.54396 | 8.730403 | -3.28436 | 0.001368 | 0.01497175 | -1.2652325 |
| LMLN | -1.2435 | 11.00862 | -3.28285 | 0.001375 | 0.01502701 | -1.2696198 |
| STK16 | -1.1459 | 10.22449 | -3.2823 | 0.001377 | 0.01503697 | -1.2712118 |
| LRRN4 | -1.33189 | 8.601258 | -3.27987 | 0.001388 | 0.01513871 | -1.2782666 |
| BCL2L10 | -1.10479 | 7.384371 | -3.27933 | 0.001391 | 0.01514843 | -1.279838 |
| CDH24 | -1.78155 | 11.37894 | -3.27643 | 0.001404 | 0.01527385 | -1.2882396 |
| SEMA5A | -10.6795 | 24.93466 | -3.27446 | 0.001413 | 0.01535454 | -1.2939654 |
| RIMS4 | -1.56651 | 10.8143 | -3.27321 | 0.001418 | 0.01538254 | -1.2975835 |
| GRWD1 | -2.48836 | 18.83298 | -3.27227 | 0.001423 | 0.01541204 | -1.3002931 |
| EPHA2 | -3.59602 | 16.11868 | -3.27185 | 0.001424 | 0.01541581 | -1.3015007 |
| F12 | -1.24874 | 8.12252 | -3.27126 | 0.001427 | 0.01542841 | -1.3032211 |
| SYT5 | -2.21634 | 9.099325 | -3.26865 | 0.001439 | 0.01552999 | -1.3107734 |
| MMP19 | -9.58938 | 23.15101 | -3.26854 | 0.00144 | 0.01552999 | -1.3110851 |
| C19orf25 | -3.18966 | 21.17928 | -3.26443 | 0.001459 | 0.01568518 | -1.3229612 |
| EPS8L3 | -1.28347 | 8.476932 | -3.26325 | 0.001464 | 0.01572761 | -1.3263675 |
| CDCA7 | -1.08686 | 7.847811 | -3.2627 | 0.001467 | 0.01573864 | -1.3279755 |
| SLC12A8 | -1.63836 | 9.173595 | -3.26066 | 0.001477 | 0.01580765 | -1.3338583 |
| FMNL1 | -4.37783 | 11.94238 | -3.26011 | 0.001479 | 0.01581831 | -1.3354393 |
| ASGR2 | -3.12491 | 13.73332 | -3.25914 | 0.001484 | 0.01583343 | -1.3382513 |
| FGD6 | -2.56607 | 11.25255 | -3.25861 | 0.001487 | 0.01583343 | -1.3397778 |
| CYGB | 2.696376 | 11.92874 | 3.258309 | 0.001488 | 0.01583343 | -1.3406371 |
| STX18 | -4.51519 | 28.79008 | -3.25783 | 0.00149 | 0.01583343 | -1.3420192 |
| SLC10A1 | -1.11314 | 7.586744 | -3.25777 | 0.001491 | 0.01583343 | -1.3421983 |
| MDFI | -1.28849 | 8.108414 | -3.25736 | 0.001493 | 0.01583343 | -1.3433866 |
| HOXD4 | 1.535273 | 10.00043 | 3.257122 | 0.001494 | 0.01583343 | -1.344062 |
| MTAP | -1.01437 | 8.897554 | -3.25616 | 0.001498 | 0.01586545 | -1.3468413 |
| PTGES2 | -2.52068 | 18.78943 | -3.25355 | 0.001511 | 0.01598082 | -1.3543626 |
| HBZ | -1.11695 | 7.969119 | -3.25323 | 0.001512 | 0.01598082 | -1.355274 |
| EHD4 | -3.97087 | 16.35921 | -3.25233 | 0.001517 | 0.01601025 | -1.357888 |
| SLAMF8 | -12.1951 | 17.68177 | -3.25136 | 0.001522 | 0.01604271 | -1.3606675 |
| KCNAB2 | -7.67998 | 21.48882 | -3.25081 | 0.001524 | 0.01604597 | -1.3622633 |
| PMAIP1 | -3.52765 | 11.47296 | -3.25001 | 0.001528 | 0.01606078 | -1.3645561 |
| PRDM10 | -2.37195 | 15.0287 | -3.24956 | 0.00153 | 0.0160669 | -1.3658555 |
| TARBP1 | -3.64703 | 18.1685 | -3.24584 | 0.001549 | 0.01622488 | -1.3765481 |
| ESPN | -1.42951 | 10.71558 | -3.24491 | 0.001553 | 0.01625624 | -1.3792352 |
| MSLN | -1.04897 | 6.942545 | -3.24437 | 0.001556 | 0.01626418 | -1.3807775 |
| KIAA1211 | -2.12763 | 9.868733 | -3.24409 | 0.001557 | 0.01626418 | -1.3815785 |
| AMELY | -1.12972 | 7.176338 | -3.24366 | 0.00156 | 0.01626954 | -1.3828252 |
| S100A3 | -1.55133 | 9.343072 | -3.24154 | 0.00157 | 0.01636318 | -1.3889245 |
| MADD | -4.22668 | 24.22554 | -3.24119 | 0.001572 | 0.01636389 | -1.3899125 |
| GAL3ST2 | -1.95321 | 10.0403 | -3.2402 | 0.001577 | 0.01639853 | -1.392758 |
| PHTF1 | -1.26973 | 10.50269 | -3.23896 | 0.001583 | 0.01644637 | -1.396318 |
| KCNK16 | -1.78269 | 9.741903 | -3.23844 | 0.001586 | 0.01645649 | -1.3978161 |
| TRPV3 | -1.09445 | 8.313031 | -3.23682 | 0.001594 | 0.01652451 | -1.4024612 |
| ITPA | -4.53343 | 33.12808 | -3.23528 | 0.001602 | 0.01658842 | -1.4068674 |
| POLD4 | -2.96588 | 22.25427 | -3.23475 | 0.001605 | 0.01659693 | -1.4083951 |
| TMOD2 | 7.426423 | 11.98736 | 3.234467 | 0.001606 | 0.01659693 | -1.4092117 |
| TXNDC2 | -1.12238 | 7.486414 | -3.23187 | 0.001619 | 0.01670157 | -1.4166473 |
| ITGA11 | -6.32263 | 18.02958 | -3.23184 | 0.00162 | 0.01670157 | -1.4167297 |
| PYGM | 2.3486 | 11.85565 | 3.229305 | 0.001633 | 0.01681999 | -1.4240047 |
| DSCR9 | -1.19216 | 7.741575 | -3.22763 | 0.001642 | 0.01689222 | -1.428785 |
| POP5 | -3.91795 | 20.1786 | -3.22689 | 0.001645 | 0.01691474 | -1.4309161 |
| ASB6 | -1.55782 | 13.88175 | -3.2261 | 0.00165 | 0.01693988 | -1.4331832 |
| PTPN6 | -4.51996 | 16.70932 | -3.22489 | 0.001656 | 0.01697921 | -1.4366269 |
| SULF2 | -10.3674 | 58.90162 | -3.22412 | 0.00166 | 0.01697921 | -1.4388429 |
| CRHR1 | -1.13977 | 7.792945 | -3.22349 | 0.001663 | 0.01698148 | -1.4406565 |
| EPN3 | -1.21017 | 7.739232 | -3.22305 | 0.001666 | 0.01698148 | -1.4419035 |
| SYNPR | -5.26347 | 8.424255 | -3.22043 | 0.00168 | 0.01710642 | -1.4494032 |
| FHL5 | 23.70061 | 58.05025 | 3.219348 | 0.001685 | 0.01714752 | -1.4524809 |
| RNPEP | -6.10113 | 27.12418 | -3.21845 | 0.00169 | 0.01717889 | -1.4550453 |
| SLC26A10 | 4.3714 | 17.54178 | 3.21771 | 0.001694 | 0.01720172 | -1.4571604 |
| ZNF510 | 1.378936 | 9.705556 | 3.216505 | 0.001701 | 0.01724513 | -1.4605992 |
| ROM1 | -3.05676 | 13.52379 | -3.21581 | 0.001705 | 0.01724513 | -1.4625788 |
| TRH | -1.78989 | 10.01309 | -3.21578 | 0.001705 | 0.01724513 | -1.4626546 |
| C14orf142 | -1.2055 | 11.13189 | -3.21544 | 0.001707 | 0.01724513 | -1.4636493 |
| PGM3 | -5.44319 | 17.58481 | -3.2153 | 0.001707 | 0.01724513 | -1.4640277 |
| PPM1F | -3.05274 | 20.31105 | -3.21151 | 0.001728 | 0.01741909 | -1.4748601 |
| NOX5 | -1.45556 | 8.286045 | -3.21063 | 0.001733 | 0.01744979 | -1.4773548 |
| PROZ | -1.25944 | 7.556658 | -3.20989 | 0.001737 | 0.01747292 | -1.4794576 |
| PIP5KL1 | -1.42182 | 9.627588 | -3.20551 | 0.001761 | 0.01770015 | -1.4919499 |
| HLA-DQB2 | -4.72216 | 14.67886 | -3.20464 | 0.001766 | 0.01771287 | -1.4944201 |
| BAAT | -1.28845 | 7.348845 | -3.20399 | 0.00177 | 0.01773157 | -1.4962772 |
| PCSK9 | -1.69504 | 11.53898 | -3.20264 | 0.001777 | 0.01778049 | -1.500094 |
| TBC1D14 | -6.01243 | 36.70612 | -3.20248 | 0.001778 | 0.01778049 | -1.5005663 |
| KCND3 | 2.080111 | 9.159053 | 3.201949 | 0.001781 | 0.01779232 | -1.5020711 |
| KRT3 | -1.3909 | 7.787936 | -3.20104 | 0.001787 | 0.01782581 | -1.5046636 |
| SCN3A | 4.675888 | 9.291145 | 3.199998 | 0.001792 | 0.01784852 | -1.5076172 |
| SYT7 | -1.84406 | 11.12529 | -3.19906 | 0.001798 | 0.01788349 | -1.5102762 |
| LRRC4 | -1.21425 | 9.282711 | -3.19804 | 0.001804 | 0.01790557 | -1.5131888 |
| ARSF | -1.15043 | 7.286962 | -3.19744 | 0.001807 | 0.01791446 | -1.514883 |
| SCML4 | -1.27213 | 6.592547 | -3.19725 | 0.001808 | 0.01791446 | -1.5154374 |
| VILL | -1.30978 | 10.61306 | -3.19596 | 0.001816 | 0.01796934 | -1.5190772 |
| DAB2 | -7.96026 | 29.89384 | -3.19398 | 0.001827 | 0.01805778 | -1.5246994 |
| DCLRE1C | -4.36797 | 19.98878 | -3.19378 | 0.001828 | 0.01805778 | -1.5252724 |
| AP3B1 | -2.65733 | 22.39351 | -3.19305 | 0.001832 | 0.01808134 | -1.5273379 |
| CRADD | -2.50685 | 17.08865 | -3.19247 | 0.001836 | 0.01809459 | -1.5289997 |
| HPSE2 | 1.810401 | 7.345802 | 3.191654 | 0.001841 | 0.01809459 | -1.5313085 |
| CALML3 | -1.54867 | 9.900734 | -3.19162 | 0.001841 | 0.01809459 | -1.531417 |
| MFAP2 | -6.62106 | 20.00817 | -3.19156 | 0.001841 | 0.01809459 | -1.5315758 |
| TBC1D8 | -4.39018 | 15.51102 | -3.19091 | 0.001845 | 0.0181068 | -1.5334311 |
| NIPSNAP3B | 3.213618 | 11.61133 | 3.188036 | 0.001862 | 0.01823724 | -1.5415635 |
| TRO | -2.8594 | 20.26838 | -3.18722 | 0.001867 | 0.01823724 | -1.5438896 |
| MRPS11 | -1.92801 | 16.08393 | -3.18719 | 0.001867 | 0.01823724 | -1.5439522 |
| NRBF2 | -1.92907 | 12.05891 | -3.1856 | 0.001876 | 0.0183111 | -1.5484583 |
| KCNJ11 | -1.44735 | 7.239918 | -3.18411 | 0.001885 | 0.01835437 | -1.5526772 |
| ADAM12 | -10.3714 | 15.681 | -3.18392 | 0.001886 | 0.01835437 | -1.5532291 |
| GOT1 | -3.20636 | 18.44767 | -3.18159 | 0.0019 | 0.01847154 | -1.5598093 |
| PLEKHB2 | -10.9686 | 36.48377 | -3.18058 | 0.001906 | 0.01849536 | -1.56268 |
| GRIA1 | -2.84683 | 7.187053 | -3.18056 | 0.001906 | 0.01849536 | -1.5627282 |
| SRPX | 10.92004 | 27.1384 | 3.178958 | 0.001916 | 0.0185709 | -1.5672571 |
| TIMM17B | -2.65503 | 19.3994 | -3.17723 | 0.001926 | 0.01863178 | -1.5721451 |
| FCER1G | -19.6764 | 38.97732 | -3.17716 | 0.001927 | 0.01863178 | -1.5723283 |
| GPR65 | -4.08816 | 12.24942 | -3.17699 | 0.001928 | 0.01863178 | -1.5728251 |
| MCM10 | -1.19888 | 7.445883 | -3.17495 | 0.00194 | 0.01870094 | -1.5785666 |
| DEFA6 | -1.01366 | 7.407284 | -3.17488 | 0.001941 | 0.01870094 | -1.5787714 |
| TSSC1 | -2.15973 | 13.01828 | -3.1741 | 0.001946 | 0.01872251 | -1.580971 |
| MAN2B1 | -10.8599 | 30.00781 | -3.1739 | 0.001947 | 0.01872251 | -1.5815522 |
| SNCG | 5.1922 | 15.6148 | 3.173386 | 0.00195 | 0.01873448 | -1.5829975 |
| VSNL1 | -4.35923 | 11.86312 | -3.17265 | 0.001954 | 0.01874126 | -1.5850652 |
| CD33 | -3.24781 | 16.26315 | -3.17198 | 0.001959 | 0.0187582 | -1.5869772 |
| SLC36A4 | -5.90964 | 15.47003 | -3.17175 | 0.00196 | 0.0187582 | -1.5876145 |
| STX1A | -2.49314 | 15.15606 | -3.17039 | 0.001968 | 0.01882077 | -1.591463 |
| ANP32C | -1.44334 | 10.07821 | -3.16984 | 0.001972 | 0.01883484 | -1.5929996 |
| DNAJC4 | -1.5397 | 12.4514 | -3.16913 | 0.001976 | 0.01885894 | -1.5950107 |
| OBSCN | -1.06642 | 8.219904 | -3.16758 | 0.001986 | 0.01893262 | -1.5993657 |
| IBSP | -6.96807 | 12.73934 | -3.16634 | 0.001994 | 0.01897267 | -1.6028655 |
| TOM1 | -4.13858 | 19.58772 | -3.1663 | 0.001994 | 0.01897267 | -1.6029861 |
| MPHOSPH9 | -1.54741 | 9.600842 | -3.16451 | 0.002005 | 0.01906151 | -1.6080285 |
| HPS1 | -1.63085 | 12.80316 | -3.16277 | 0.002016 | 0.01914762 | -1.6129229 |
| SLITRK1 | -1.28686 | 7.00008 | -3.15879 | 0.002042 | 0.01937008 | -1.6241152 |
| OSBPL7 | -1.69596 | 13.55455 | -3.15779 | 0.002048 | 0.01941237 | -1.6269263 |
| CPA4 | -1.49061 | 8.582355 | -3.15748 | 0.00205 | 0.01941272 | -1.6278029 |
| CHIA | -1.2183 | 7.401187 | -3.15674 | 0.002055 | 0.01943904 | -1.6298736 |
| E2F4 | -3.91928 | 25.22718 | -3.155 | 0.002066 | 0.01952335 | -1.6347794 |
| CORO2A | -1.04769 | 7.635491 | -3.15326 | 0.002077 | 0.01958189 | -1.6396667 |
| LGALS2 | -3.16423 | 10.24175 | -3.15319 | 0.002078 | 0.01958189 | -1.6398485 |
| SCN7A | 12.78178 | 19.85442 | 3.151842 | 0.002087 | 0.01964287 | -1.6436405 |
| PSMC4 | -3.18238 | 23.51438 | -3.15157 | 0.002089 | 0.01964287 | -1.6444077 |
| PRIMA1 | 6.56472 | 15.11083 | 3.151 | 0.002092 | 0.01964287 | -1.6460015 |
| SOST | 7.911865 | 15.69083 | 3.150875 | 0.002093 | 0.01964287 | -1.6463533 |
| TRADD | -6.17598 | 47.49478 | -3.15068 | 0.002094 | 0.01964287 | -1.6468953 |
| B3GAT2 | -6.9096 | 9.128364 | -3.14722 | 0.002117 | 0.01983832 | -1.6565965 |
| LMAN2 | -3.33816 | 23.04253 | -3.14569 | 0.002127 | 0.01987671 | -1.660894 |
| BAK1 | -1.80512 | 13.35784 | -3.1454 | 0.002129 | 0.01987671 | -1.6616975 |
| SLC12A6 | -6.48152 | 27.86001 | -3.14526 | 0.00213 | 0.01987671 | -1.6620931 |
| DR1 | -5.15951 | 24.15722 | -3.14509 | 0.002131 | 0.01987671 | -1.6625661 |
| GNG13 | -1.23924 | 6.836731 | -3.14394 | 0.002139 | 0.01990971 | -1.6657896 |
| TEKT3 | -1.00287 | 6.320423 | -3.14385 | 0.00214 | 0.01990971 | -1.6660536 |
| SLC6A12 | -1.54554 | 8.370463 | -3.14366 | 0.002141 | 0.01990971 | -1.6665841 |
| RTN4RL2 | -1.38041 | 9.757343 | -3.14245 | 0.002149 | 0.0199667 | -1.6699815 |
| GDA | -5.02585 | 8.218141 | -3.14169 | 0.002154 | 0.01999508 | -1.6720936 |
| ALOX15B | -2.67926 | 10.19685 | -3.14043 | 0.002163 | 0.02005558 | -1.675635 |
| FADD | -2.16165 | 13.62938 | -3.13926 | 0.002171 | 0.0200797 | -1.6788941 |
| GPR19 | -1.30131 | 6.735148 | -3.1379 | 0.00218 | 0.0201392 | -1.6827117 |
| VAV1 | -7.39663 | 15.88228 | -3.13703 | 0.002186 | 0.02016098 | -1.6851344 |
| MYOD1 | -1.79799 | 10.43002 | -3.13695 | 0.002186 | 0.02016098 | -1.6853521 |
| ZNF557 | -1.11091 | 8.160323 | -3.13658 | 0.002189 | 0.02016544 | -1.6863858 |
| GALNT9 | -1.21708 | 8.086907 | -3.13613 | 0.002192 | 0.02017497 | -1.687643 |
| ACY1 | -1.592 | 10.41942 | -3.13391 | 0.002207 | 0.02028352 | -1.6938562 |
| ALG12 | -1.48363 | 12.41293 | -3.13382 | 0.002208 | 0.02028352 | -1.694105 |
| GPR68 | -1.48926 | 9.190377 | -3.13103 | 0.002227 | 0.02044186 | -1.7018836 |
| SEMA7A | -1.62282 | 12.02569 | -3.13004 | 0.002234 | 0.02048612 | -1.7046474 |
| LTA | -1.72713 | 10.31734 | -3.12951 | 0.002238 | 0.0205013 | -1.7061401 |
| GDF5 | -2.0528 | 12.59447 | -3.12778 | 0.00225 | 0.02059333 | -1.7109705 |
| DOT1L | -2.59736 | 10.98165 | -3.12662 | 0.002258 | 0.02062132 | -1.7142127 |
| PURG | -1.8655 | 8.093276 | -3.12635 | 0.00226 | 0.02062132 | -1.7149532 |
| TIMM13 | -3.00123 | 16.80479 | -3.12622 | 0.002261 | 0.02062132 | -1.7153228 |
| MIPEP | -1.74724 | 11.74224 | -3.12565 | 0.002265 | 0.02062132 | -1.7169056 |
| NAGA | -4.30663 | 20.9896 | -3.12556 | 0.002266 | 0.02062132 | -1.7171491 |
| MYBBP1A | -1.70939 | 14.32578 | -3.12434 | 0.002274 | 0.02068112 | -1.7205596 |
| AP1S1 | -2.62544 | 17.36665 | -3.12198 | 0.002291 | 0.02077606 | -1.7271179 |
| CACNG5 | -1.46966 | 9.638076 | -3.12198 | 0.002291 | 0.02077606 | -1.7271193 |
| KIAA0125 | -1.0364 | 7.999322 | -3.12117 | 0.002297 | 0.02080982 | -1.7293905 |
| USP31 | -3.87326 | 15.92783 | -3.11893 | 0.002313 | 0.0208954 | -1.7356202 |
| CRYGN | -1.225 | 7.88441 | -3.11887 | 0.002313 | 0.0208954 | -1.7357859 |
| AVIL | -3.30701 | 20.63068 | -3.11791 | 0.00232 | 0.02091334 | -1.7384695 |
| BMP2K | -5.24521 | 17.74629 | -3.11781 | 0.002321 | 0.02091334 | -1.7387345 |
| SIRT3 | -2.64519 | 21.62478 | -3.11704 | 0.002327 | 0.02094467 | -1.7408873 |
| SULT1C2 | -1.97888 | 8.113279 | -3.1155 | 0.002338 | 0.02102597 | -1.745161 |
| CSF2RA | -2.28632 | 9.839679 | -3.11231 | 0.002361 | 0.02121666 | -1.7540337 |
| EMILIN2 | -10.5942 | 22.82412 | -3.11113 | 0.00237 | 0.0212749 | -1.7572938 |
| SLIT1 | -1.09762 | 7.773319 | -3.11042 | 0.002375 | 0.02130296 | -1.7592833 |
| SNAI3 | -1.72108 | 11.43783 | -3.11001 | 0.002378 | 0.02131033 | -1.7604043 |
| VLDLR | 3.060806 | 13.63485 | 3.107813 | 0.002394 | 0.02143703 | -1.7665054 |
| MAPK11 | -1.33442 | 10.62209 | -3.1075 | 0.002397 | 0.02143844 | -1.7673751 |
| ASCC1 | -3.17615 | 22.40771 | -3.10602 | 0.002408 | 0.02151756 | -1.7714717 |
| CXCL16 | -18.2083 | 38.29153 | -3.10443 | 0.00242 | 0.02160465 | -1.7758847 |
| SELPLG | -5.76835 | 18.22075 | -3.10389 | 0.002424 | 0.02160677 | -1.7773881 |
| ACOXL | -1.01053 | 6.812072 | -3.10353 | 0.002427 | 0.02160677 | -1.7783776 |
| MPP3 | -1.41979 | 10.17238 | -3.10328 | 0.002428 | 0.02160677 | -1.7790802 |
| PLK3 | -6.24205 | 21.91419 | -3.10323 | 0.002429 | 0.02160677 | -1.7792015 |
| LZTR1 | -5.6364 | 50.56356 | -3.10263 | 0.002433 | 0.02162801 | -1.7808835 |
| SYNGR2 | -7.59674 | 32.19741 | -3.10195 | 0.002438 | 0.02165388 | -1.7827548 |
| NRP2 | -2.89504 | 13.67405 | -3.0998 | 0.002455 | 0.02174042 | -1.7887223 |
| ENG | -11.5412 | 45.71332 | -3.09935 | 0.002458 | 0.02175098 | -1.7899574 |
| EBF2 | 1.917464 | 9.25634 | 3.098088 | 0.002468 | 0.02181674 | -1.7934507 |
| CDKL3 | -1.22144 | 10.7869 | -3.0977 | 0.002471 | 0.02182114 | -1.794514 |
| TAP1 | -9.49922 | 43.52601 | -3.09354 | 0.002503 | 0.02202759 | -1.8060224 |
| TM6SF1 | -7.32117 | 20.74427 | -3.08724 | 0.002552 | 0.0224103 | -1.8234315 |
| SLC25A22 | -1.77588 | 13.47408 | -3.08712 | 0.002553 | 0.0224103 | -1.8237671 |
| PCBP3 | -1.69638 | 10.57965 | -3.08429 | 0.002576 | 0.02255018 | -1.8315684 |
| ZNF436 | -4.52393 | 21.23436 | -3.0841 | 0.002577 | 0.02255018 | -1.8320789 |
| SPA17 | -1.87017 | 10.44833 | -3.08395 | 0.002578 | 0.02255018 | -1.8324859 |
| LRFN4 | -1.98952 | 16.02039 | -3.08273 | 0.002588 | 0.02260752 | -1.8358611 |
| SYN1 | -1.95365 | 10.93136 | -3.08229 | 0.002592 | 0.02260752 | -1.8370582 |
| CHGB | -1.71354 | 6.877347 | -3.08227 | 0.002592 | 0.02260752 | -1.837121 |
| KCNE2 | 1.160564 | 7.420701 | 3.080663 | 0.002605 | 0.02270011 | -1.8415526 |
| USP49 | -1.91063 | 14.27516 | -3.08007 | 0.002609 | 0.02272158 | -1.8431834 |
| SEC23B | -4.08255 | 24.67538 | -3.07898 | 0.002618 | 0.02276057 | -1.8461789 |
| MPV17 | -3.66022 | 26.51645 | -3.07847 | 0.002622 | 0.02277367 | -1.847585 |
| DLL3 | -1.07518 | 8.287065 | -3.07593 | 0.002643 | 0.02289907 | -1.8545731 |
| SOX21 | -1.37662 | 8.610055 | -3.07584 | 0.002644 | 0.02289907 | -1.8548321 |
| SLC39A3 | -2.42177 | 18.21439 | -3.07518 | 0.002649 | 0.02292547 | -1.8566421 |
| LCAT | -1.8292 | 13.57293 | -3.07378 | 0.002661 | 0.02298354 | -1.8604856 |
| PLCB2 | -4.85615 | 20.83234 | -3.07369 | 0.002661 | 0.02298354 | -1.8607325 |
| LILRB3 | -8.95054 | 30.82507 | -3.07345 | 0.002663 | 0.02298354 | -1.8613913 |
| ADCK2 | -2.0092 | 15.27109 | -3.07322 | 0.002665 | 0.02298354 | -1.8620223 |
| PAX2 | -1.28185 | 7.906339 | -3.0725 | 0.002671 | 0.02301493 | -1.8640179 |
| MRPL36 | -12.3811 | 91.85928 | -3.07125 | 0.002682 | 0.02308362 | -1.8674519 |
| PHLDA1 | -11.3343 | 36.30931 | -3.07055 | 0.002687 | 0.02311299 | -1.8693629 |
| DEFB118 | -2.58779 | 15.80058 | -3.06816 | 0.002707 | 0.02326351 | -1.8759224 |
| SNAI1 | -2.21145 | 13.10623 | -3.06786 | 0.00271 | 0.02326463 | -1.8767425 |
| ZDHHC1 | -1.21043 | 8.957211 | -3.06555 | 0.002729 | 0.02341029 | -1.8830775 |
| FOXA3 | -1.10054 | 6.72483 | -3.06212 | 0.002758 | 0.02361818 | -1.892501 |
| FOLR2 | -6.91551 | 24.39776 | -3.06157 | 0.002763 | 0.02363716 | -1.8939902 |
| ATCAY | -2.27146 | 12.74763 | -3.05936 | 0.002781 | 0.02377785 | -1.9000474 |
| SYT15 | -3.9922 | 23.96839 | -3.05691 | 0.002802 | 0.02393659 | -1.9067439 |
| HHLA1 | -1.15474 | 7.489315 | -3.05648 | 0.002806 | 0.0239476 | -1.907925 |
| TBX10 | -1.17336 | 10.14039 | -3.0559 | 0.002811 | 0.02396988 | -1.9095239 |
| CPNE6 | -1.92818 | 9.854128 | -3.05288 | 0.002837 | 0.02414655 | -1.9177816 |
| GBX2 | -1.17041 | 9.611615 | -3.05248 | 0.002841 | 0.02414655 | -1.9188607 |
| PNMA3 | -1.55538 | 10.14453 | -3.05203 | 0.002845 | 0.02414799 | -1.9201048 |
| RPL3L | -1.68076 | 11.18871 | -3.0518 | 0.002847 | 0.02414799 | -1.9207259 |
| FZD5 | -1.16135 | 9.095499 | -3.0481 | 0.002879 | 0.02440271 | -1.9308304 |
| PLA2G2D | -2.05413 | 11.94159 | -3.0474 | 0.002886 | 0.02443404 | -1.9327379 |
| PRKCG | -1.02265 | 7.190388 | -3.04712 | 0.002888 | 0.02443422 | -1.933509 |
| ARHGDIA | -4.92006 | 40.7242 | -3.04538 | 0.002903 | 0.02452272 | -1.9382539 |
| CYB561 | -2.97834 | 18.55669 | -3.04388 | 0.002917 | 0.02461372 | -1.9423469 |
| PGLS | -3.59232 | 19.7075 | -3.04357 | 0.00292 | 0.02461372 | -1.9431901 |
| CLPTM1 | -2.5047 | 21.24178 | -3.0403 | 0.002949 | 0.02482216 | -1.9520985 |
| FRS3 | -1.58278 | 11.839 | -3.03988 | 0.002953 | 0.02483266 | -1.9532347 |
| POLR2G | -8.98181 | 90.56838 | -3.03917 | 0.002959 | 0.02486573 | -1.9551778 |
| ACO2 | 7.898238 | 30.2606 | 3.037683 | 0.002973 | 0.02493664 | -1.9592274 |
| SLC25A20 | -3.00671 | 15.67198 | -3.03724 | 0.002977 | 0.02494885 | -1.9604201 |
| PCP4 | 7.211145 | 10.28834 | 3.035216 | 0.002995 | 0.02508279 | -1.9659389 |
| CHRNA2 | -2.49028 | 14.63781 | -3.03478 | 0.002999 | 0.02509502 | -1.9671284 |
| PLVAP | 4.174615 | 21.20901 | 3.034067 | 0.003006 | 0.02512838 | -1.9690649 |
| ENC1 | -6.58329 | 21.97796 | -3.03241 | 0.003021 | 0.02521483 | -1.9735567 |
| SEMA3E | 3.241095 | 12.73805 | 3.032174 | 0.003023 | 0.02521483 | -1.9742088 |
| ABI3 | -3.29959 | 19.49747 | -3.03188 | 0.003026 | 0.02521483 | -1.9750209 |
| WRNIP1 | -2.04037 | 19.22224 | -3.03183 | 0.003026 | 0.02521483 | -1.9751337 |
| GRM4 | -1.7965 | 11.42169 | -3.03142 | 0.00303 | 0.02522578 | -1.9762717 |
| GPBAR1 | -1.7115 | 10.49265 | -3.03041 | 0.00304 | 0.02523424 | -1.9790121 |
| SPATS1 | -1.17507 | 7.653924 | -3.02941 | 0.003049 | 0.02525553 | -1.9817267 |
| INHBE | -1.00801 | 6.997162 | -3.02937 | 0.003049 | 0.02525553 | -1.9818181 |
| ARHGEF5 | -2.23447 | 13.86609 | -3.02787 | 0.003063 | 0.02534649 | -1.9858875 |
| HTR5A | -1.35258 | 7.795747 | -3.02765 | 0.003065 | 0.02534649 | -1.9865088 |
| GMPPA | -2.58431 | 17.65342 | -3.02686 | 0.003073 | 0.02538574 | -1.9886303 |
| NBEA | 3.512667 | 10.94119 | 3.025643 | 0.003084 | 0.02545058 | -1.9919411 |
| KLHL8 | -4.02231 | 21.19952 | -3.02449 | 0.003095 | 0.02550636 | -1.9950788 |
| GJA8 | -2.04358 | 10.41086 | -3.02084 | 0.00313 | 0.02576961 | -2.004949 |
| SLC28A3 | -1.20961 | 7.459638 | -3.01923 | 0.003145 | 0.02587495 | -2.0093173 |
| MEGF11 | -1.19356 | 7.427772 | -3.01672 | 0.003169 | 0.02605164 | -2.0161072 |
| NBL1 | -17.5334 | 65.73985 | -3.01389 | 0.003197 | 0.02625581 | -2.023785 |
| VPREB3 | -1.3574 | 8.609687 | -3.01328 | 0.003203 | 0.02628189 | -2.0254077 |
| LTBR | -2.25285 | 20.12151 | -3.01268 | 0.003208 | 0.026308 | -2.0270297 |
| PGPEP1 | -2.30109 | 16.61988 | -3.01184 | 0.003217 | 0.02635358 | -2.0293071 |
| CRYL1 | -5.03228 | 19.47399 | -3.01017 | 0.003233 | 0.02645898 | -2.0338168 |
| HS3ST4 | -2.03547 | 8.476405 | -3.00998 | 0.003235 | 0.02645898 | -2.0343297 |
| GPSM3 | -3.16165 | 15.23281 | -3.00842 | 0.00325 | 0.02656096 | -2.0385505 |
| SELT | -7.43523 | 44.64235 | -3.00814 | 0.003253 | 0.02656096 | -2.0393182 |
| RNF25 | -2.25176 | 13.8804 | -3.0079 | 0.003255 | 0.02656096 | -2.0399575 |
| TUSC2 | -2.56581 | 22.60477 | -3.00739 | 0.00326 | 0.02656477 | -2.0413331 |
| MELK | -2.93793 | 10.26608 | -3.00717 | 0.003263 | 0.02656477 | -2.0419204 |
| HMGA1 | -3.85801 | 17.84346 | -3.00703 | 0.003264 | 0.02656477 | -2.0422899 |
| KCNG4 | -1.69425 | 9.349185 | -3.00647 | 0.00327 | 0.02658363 | -2.0438036 |
| TES | -9.03943 | 34.28957 | -3.00626 | 0.003272 | 0.02658363 | -2.044387 |
| MAPK8IP2 | -1.18454 | 8.827094 | -3.00423 | 0.003292 | 0.02670361 | -2.0498504 |
| CABYR | -1.21541 | 10.15203 | -3.00385 | 0.003296 | 0.02671223 | -2.0508684 |
| C2 | -5.38492 | 15.70497 | -2.99913 | 0.003343 | 0.02703141 | -2.0636052 |
| KIFC3 | -3.05852 | 21.56656 | -2.99867 | 0.003348 | 0.02704698 | -2.0648452 |
| MMAA | -1.28483 | 8.716694 | -2.99804 | 0.003354 | 0.02707589 | -2.0665216 |
| FMR1NB | -1.23535 | 7.610448 | -2.99691 | 0.003366 | 0.02713811 | -2.0695757 |
| ZNF576 | -1.41803 | 13.73199 | -2.99613 | 0.003374 | 0.0271669 | -2.071681 |
| RAB9B | 1.950722 | 12.93764 | 2.995829 | 0.003377 | 0.02716917 | -2.0724807 |
| FOLR1 | -1.79788 | 8.017371 | -2.99355 | 0.0034 | 0.02733539 | -2.0786199 |
| C6orf136 | -1.70312 | 13.73785 | -2.99185 | 0.003418 | 0.02741132 | -2.0831927 |
| TIAL1 | -6.11109 | 42.27025 | -2.99158 | 0.003421 | 0.02741132 | -2.0838961 |
| DEDD2 | -6.57455 | 29.47852 | -2.99155 | 0.003421 | 0.02741132 | -2.0839754 |
| DIAPH1 | -7.11038 | 42.74433 | -2.99073 | 0.003429 | 0.0274574 | -2.0861878 |
| SLC5A6 | -3.7277 | 22.61534 | -2.9899 | 0.003438 | 0.02748754 | -2.0884158 |
| OBP2B | -1.11859 | 7.777521 | -2.98983 | 0.003439 | 0.02748754 | -2.0886034 |
| ESRRA | -2.16803 | 16.28349 | -2.9895 | 0.003442 | 0.02749305 | -2.0895013 |
| SYMPK | -4.91552 | 21.50506 | -2.98918 | 0.003446 | 0.02749721 | -2.0903551 |
| GRAP | -1.03824 | 7.627162 | -2.98824 | 0.003455 | 0.02755291 | -2.0928695 |
| NUDT15 | -1.694 | 11.64275 | -2.98783 | 0.00346 | 0.02756526 | -2.0939853 |
| SLC31A1 | -6.76366 | 23.65781 | -2.98749 | 0.003463 | 0.02757141 | -2.0949012 |
| GLYCTK | -1.22144 | 10.31863 | -2.987 | 0.003468 | 0.02758984 | -2.0962112 |
| EIF4G3 | -3.59328 | 27.62701 | -2.98588 | 0.00348 | 0.02766123 | -2.0992206 |
| FHOD1 | -4.57436 | 19.43155 | -2.97963 | 0.003546 | 0.02814237 | -2.1159498 |
| PPP1R9A | 3.493153 | 7.252453 | 2.977913 | 0.003565 | 0.02820378 | -2.1205556 |
| NADSYN1 | -2.09218 | 15.73598 | -2.97758 | 0.003568 | 0.02820378 | -2.1214497 |
| PRAME | -1.00883 | 6.496334 | -2.97659 | 0.003579 | 0.02822707 | -2.1240832 |
| TBX6 | -1.26769 | 8.853663 | -2.97651 | 0.00358 | 0.02822707 | -2.1243145 |
| DCLRE1A | -1.56005 | 11.49592 | -2.97609 | 0.003584 | 0.02824008 | -2.1254328 |
| TGM1 | -2.26208 | 11.90935 | -2.97422 | 0.003605 | 0.02837715 | -2.1304362 |
| MSI1 | -1.03916 | 8.012697 | -2.97394 | 0.003608 | 0.02837832 | -2.1311811 |
| SLC30A6 | -1.75374 | 14.38387 | -2.97185 | 0.003631 | 0.02851589 | -2.1367764 |
| ACVR1B | -1.74123 | 12.48 | -2.97107 | 0.003639 | 0.02855665 | -2.1388582 |
| LRRC1 | 4.009439 | 16.13789 | 2.969283 | 0.003659 | 0.02866498 | -2.143627 |
| AASS | 5.621995 | 19.29789 | 2.967461 | 0.003679 | 0.02879968 | -2.148488 |
| POFUT1 | -1.50194 | 13.39701 | -2.96657 | 0.003689 | 0.02885394 | -2.150861 |
| KLHL6 | -5.09755 | 11.87215 | -2.96581 | 0.003697 | 0.02889507 | -2.1528955 |
| KMO | -3.67222 | 8.536631 | -2.96557 | 0.0037 | 0.02889507 | -2.1535307 |
| COL2A1 | -1.10668 | 7.631869 | -2.96436 | 0.003713 | 0.02897787 | -2.1567697 |
| TOP3B | -1.26237 | 11.02663 | -2.96369 | 0.003721 | 0.02901264 | -2.1585344 |
| PRDM16 | 5.955769 | 14.99276 | 2.961925 | 0.003741 | 0.02914435 | -2.1632504 |
| NODAL | -1.1252 | 9.127233 | -2.96138 | 0.003747 | 0.02916944 | -2.1647137 |
| FOXI1 | -1.2481 | 10.62892 | -2.96073 | 0.003754 | 0.02920327 | -2.1664413 |
| NNAT | -3.74309 | 14.86757 | -2.95969 | 0.003766 | 0.02924779 | -2.16919 |
| SFXN2 | -1.16799 | 10.41636 | -2.95914 | 0.003772 | 0.02927371 | -2.1706737 |
| RBP1 | -5.38545 | 20.54947 | -2.9581 | 0.003784 | 0.02927859 | -2.1734235 |
| GALK1 | -1.82676 | 12.53066 | -2.95806 | 0.003784 | 0.02927859 | -2.1735498 |
| BARX2 | -1.20252 | 9.286079 | -2.95803 | 0.003785 | 0.02927859 | -2.1736099 |
| MPPE1 | -3.45067 | 33.0133 | -2.95711 | 0.003795 | 0.02933679 | -2.1760654 |
| CDS1 | -1.14587 | 8.52337 | -2.95482 | 0.003821 | 0.02947075 | -2.1821592 |
| CRYBB3 | -2.14219 | 11.17484 | -2.95191 | 0.003855 | 0.02970528 | -2.1899009 |
| SFXN1 | -3.62713 | 20.2067 | -2.95021 | 0.003875 | 0.02983346 | -2.1944075 |
| ACP2 | -7.92471 | 25.60144 | -2.94918 | 0.003887 | 0.02987929 | -2.197151 |
| RAMP3 | 3.656509 | 12.96896 | 2.948084 | 0.003899 | 0.02995397 | -2.2000531 |
| GDF10 | 3.01871 | 15.25706 | 2.947641 | 0.003905 | 0.02997042 | -2.2012298 |
| MRPL15 | -6.30995 | 30.58611 | -2.94629 | 0.00392 | 0.03004495 | -2.2048086 |
| TFG | -6.83694 | 50.78127 | -2.94529 | 0.003932 | 0.03011168 | -2.2074622 |
| ZAR1 | -1.06618 | 9.348955 | -2.94317 | 0.003957 | 0.03017733 | -2.2130771 |
| PNLIPRP1 | -1.11504 | 7.126621 | -2.9428 | 0.003962 | 0.03017733 | -2.2140613 |
| ICAM4 | -1.09127 | 7.418362 | -2.94275 | 0.003962 | 0.03017733 | -2.2141872 |
| TGFB1 | -4.29516 | 21.88495 | -2.94265 | 0.003963 | 0.03017733 | -2.2144543 |
| SPINK4 | -1.29503 | 8.339977 | -2.94257 | 0.003964 | 0.03017733 | -2.2146776 |
| PPP1R3F | -1.08811 | 10.67798 | -2.94252 | 0.003965 | 0.03017733 | -2.2148048 |
| SYNGR4 | -1.34177 | 9.187276 | -2.94249 | 0.003965 | 0.03017733 | -2.2148816 |
| ZNF442 | 1.458843 | 9.227889 | 2.941761 | 0.003974 | 0.03021537 | -2.2168186 |
| DNAJC5B | -1.82123 | 8.093645 | -2.94008 | 0.003994 | 0.03030235 | -2.2212743 |
| UBE2D1 | -3.52513 | 15.02024 | -2.93847 | 0.004013 | 0.0304248 | -2.2255262 |
| ZNF488 | -1.212 | 7.937067 | -2.93802 | 0.004019 | 0.03042853 | -2.2267251 |
| C1RL | -4.67925 | 21.81137 | -2.93792 | 0.00402 | 0.03042853 | -2.2269966 |
| PIGO | -1.89819 | 13.4983 | -2.9368 | 0.004033 | 0.03050412 | -2.2299597 |
| NEDD4 | 1.85977 | 11.40563 | 2.936573 | 0.004036 | 0.03050412 | -2.2305522 |
| NPPB | -1.19449 | 7.818569 | -2.93326 | 0.004076 | 0.03076064 | -2.2393213 |
| RAB5C | -2.72345 | 23.84021 | -2.93235 | 0.004087 | 0.03077328 | -2.2417169 |
| SYNGR3 | -4.42869 | 12.85564 | -2.93132 | 0.0041 | 0.03079709 | -2.2444286 |
| OR7C2 | -1.12069 | 7.359119 | -2.93043 | 0.004111 | 0.03083215 | -2.2467843 |
| ALDH1B1 | 10.62644 | 35.02568 | 2.929882 | 0.004117 | 0.03084041 | -2.2482319 |
| MCCC2 | -1.24652 | 11.7765 | -2.92966 | 0.00412 | 0.03084041 | -2.2488251 |
| PLXDC2 | -13.9383 | 49.60082 | -2.92958 | 0.004121 | 0.03084041 | -2.2490406 |
| SLC7A11 | -23.3269 | 263.0619 | -2.92906 | 0.004128 | 0.03084132 | -2.2504105 |
| DCTN2 | -3.3466 | 36.33406 | -2.92826 | 0.004137 | 0.03089089 | -2.2525023 |
| WDTC1 | -2.17088 | 19.68418 | -2.92674 | 0.004156 | 0.03096094 | -2.2565164 |
| H2AFY2 | -3.10099 | 22.59064 | -2.92634 | 0.004161 | 0.03097462 | -2.2575759 |
| GMEB1 | -2.70768 | 15.16121 | -2.92397 | 0.004191 | 0.03114744 | -2.2638341 |
| TRIM17 | -1.03405 | 7.355548 | -2.92189 | 0.004217 | 0.03129385 | -2.2693161 |
| CBX4 | -5.62871 | 22.84704 | -2.92086 | 0.00423 | 0.03132265 | -2.2720236 |
| SMO | -1.86295 | 14.96868 | -2.92032 | 0.004237 | 0.03134565 | -2.2734408 |
| TAF12 | -1.41186 | 16.24037 | -2.91869 | 0.004257 | 0.03146834 | -2.2777289 |
| PKIB | -2.85727 | 7.164736 | -2.9185 | 0.004259 | 0.03146834 | -2.2782233 |
| CPEB1 | 3.377677 | 11.92553 | 2.916466 | 0.004285 | 0.03161202 | -2.2835772 |
| SHBG | -2.01728 | 11.34433 | -2.91154 | 0.004349 | 0.03198699 | -2.2965145 |
| FAT3 | 8.438989 | 12.76528 | 2.911355 | 0.004351 | 0.03198699 | -2.2970066 |
| MTMR1 | -4.7767 | 24.1724 | -2.91114 | 0.004354 | 0.03198699 | -2.2975649 |
| ADAM9 | -16.9888 | 67.09251 | -2.91099 | 0.004356 | 0.03198699 | -2.2979669 |
| SNX27 | -2.92385 | 28.12036 | -2.9082 | 0.004392 | 0.03221829 | -2.305284 |
| PIGC | -3.16385 | 20.2932 | -2.90806 | 0.004394 | 0.03221829 | -2.3056526 |
| FLT3LG | -1.64146 | 14.37585 | -2.90746 | 0.004402 | 0.03223791 | -2.3072225 |
| PGS1 | -5.35687 | 23.21553 | -2.90656 | 0.004413 | 0.03224599 | -2.3095818 |
| FAM3D | -1.53217 | 9.414515 | -2.90652 | 0.004414 | 0.03224599 | -2.3096964 |
| EMCN | 3.073624 | 11.63123 | 2.904667 | 0.004438 | 0.03239952 | -2.3145493 |
| CTAG2 | -2.52744 | 16.6229 | -2.9041 | 0.004446 | 0.03242998 | -2.3160344 |
| C4BPB | -1.23131 | 7.770056 | -2.90377 | 0.00445 | 0.03243732 | -2.3168886 |
| GAD1 | 2.819431 | 8.548476 | 2.902055 | 0.004473 | 0.03257901 | -2.3213936 |
| KIAA0922 | 5.611134 | 21.19844 | 2.901445 | 0.004481 | 0.03261377 | -2.3229892 |
| MTA2 | -2.27139 | 18.26096 | -2.90077 | 0.00449 | 0.03265494 | -2.3247566 |
| SLC29A3 | -4.12682 | 15.50177 | -2.89993 | 0.004501 | 0.03271182 | -2.3269464 |
| AMPD3 | -7.19026 | 20.70586 | -2.89428 | 0.004577 | 0.03320234 | -2.3417213 |
| PPFIBP2 | -3.16784 | 20.79159 | -2.89267 | 0.004599 | 0.03332455 | -2.3459418 |
| ERAL1 | -1.364 | 13.75032 | -2.89201 | 0.004608 | 0.03334014 | -2.3476537 |
| FASN | -3.2707 | 19.08886 | -2.89054 | 0.004628 | 0.03343607 | -2.3514876 |
| SLC39A5 | -2.59601 | 14.76388 | -2.88983 | 0.004637 | 0.03346052 | -2.3533441 |
| NCBP2 | -6.1845 | 44.88527 | -2.88965 | 0.00464 | 0.03346052 | -2.3538146 |
| NPHP4 | -1.16208 | 10.48919 | -2.88955 | 0.004641 | 0.03346052 | -2.3540743 |
| PLA2R1 | -2.90764 | 15.05313 | -2.88904 | 0.004648 | 0.03348643 | -2.355404 |
| C6orf47 | -3.49834 | 26.98754 | -2.88762 | 0.004668 | 0.03360275 | -2.3591112 |
| ALPP | -1.35105 | 7.977074 | -2.88725 | 0.004673 | 0.03361509 | -2.3600805 |
| GPR135 | -2.01304 | 13.41098 | -2.88684 | 0.004678 | 0.03363055 | -2.3611311 |
| GALNT12 | -2.27553 | 9.078995 | -2.88428 | 0.004714 | 0.03386152 | -2.3678174 |
| FUK | -1.70693 | 14.62742 | -2.88347 | 0.004725 | 0.03391735 | -2.3699153 |
| MMP21 | -1.28284 | 8.653287 | -2.8827 | 0.004736 | 0.03397046 | -2.3719395 |
| METTL1 | -2.54823 | 15.4266 | -2.87945 | 0.004782 | 0.03419778 | -2.3803931 |
| RUVBL1 | -1.72144 | 13.1766 | -2.87752 | 0.004809 | 0.03435917 | -2.3854137 |
| RBPMS2 | 29.29163 | 129.402 | 2.877357 | 0.004811 | 0.03435917 | -2.3858286 |
| ICAM1 | -3.93973 | 15.44177 | -2.87706 | 0.004815 | 0.03436433 | -2.3866009 |
| KLK11 | -1.16289 | 7.715842 | -2.87653 | 0.004823 | 0.03437643 | -2.3879742 |
| CRYGD | -2.46861 | 13.30685 | -2.87631 | 0.004826 | 0.03437643 | -2.3885365 |
| ARRDC1 | -1.73951 | 13.35712 | -2.87596 | 0.004831 | 0.03437643 | -2.3894653 |
| SLC3A2 | -6.55185 | 43.15496 | -2.87413 | 0.004857 | 0.03453647 | -2.3942006 |
| TBL2 | -4.04856 | 29.85951 | -2.87359 | 0.004865 | 0.0345606 | -2.3956016 |
| PPP1R12C | 3.049337 | 21.46487 | 2.873407 | 0.004868 | 0.0345606 | -2.3960898 |
| WNT5A | -4.89782 | 14.55442 | -2.87083 | 0.004905 | 0.03476647 | -2.4027828 |
| DOCK3 | 1.685737 | 7.598621 | 2.870654 | 0.004907 | 0.03476647 | -2.403235 |
| NANOS1 | -1.0189 | 8.016383 | -2.86914 | 0.004929 | 0.03489661 | -2.4071651 |
| MRPL41 | -2.78779 | 26.18885 | -2.86772 | 0.00495 | 0.03501672 | -2.4108293 |
| NPAS2 | -1.11713 | 9.992715 | -2.86675 | 0.004964 | 0.03509205 | -2.4133578 |
| MAGEA10 | -1.02248 | 6.163523 | -2.86105 | 0.005048 | 0.03563291 | -2.4281097 |
| PGAM2 | 2.649236 | 13.82461 | 2.859507 | 0.00507 | 0.03576903 | -2.4321027 |
| DCBLD1 | -5.57314 | 21.58608 | -2.8533 | 0.005164 | 0.03631785 | -2.4481463 |
| CACNA1I | -1.44677 | 10.40126 | -2.85309 | 0.005167 | 0.03631785 | -2.4486751 |
| STIM2 | -1.45147 | 12.89442 | -2.85276 | 0.005172 | 0.03632674 | -2.4495196 |
| CDSN | -1.41039 | 9.815684 | -2.85142 | 0.005192 | 0.03636572 | -2.4529796 |
| MOG | -3.15082 | 7.491447 | -2.85115 | 0.005196 | 0.03636572 | -2.4536837 |
| IL18BP | -2.09564 | 14.22423 | -2.85109 | 0.005197 | 0.03636572 | -2.4538375 |
| ABCC10 | -3.65927 | 20.66234 | -2.85105 | 0.005198 | 0.03636572 | -2.4539421 |
| TST | -5.51481 | 25.52173 | -2.85094 | 0.005199 | 0.03636572 | -2.4542277 |
| MED8 | -2.66716 | 22.37702 | -2.85013 | 0.005212 | 0.03642646 | -2.4563237 |
| SUSD3 | -1.39093 | 9.48801 | -2.84902 | 0.005229 | 0.03649263 | -2.4591724 |
| ALG8 | -3.97965 | 25.74894 | -2.84849 | 0.005237 | 0.03652349 | -2.4605414 |
| COG1 | -1.45879 | 14.28556 | -2.84717 | 0.005257 | 0.03662653 | -2.4639327 |
| FOXQ1 | -4.89251 | 14.02303 | -2.84704 | 0.005259 | 0.03662653 | -2.464281 |
| PTPRA | -1.50665 | 16.79481 | -2.84644 | 0.005268 | 0.03662653 | -2.4658165 |
| ARHGDIG | -1.74302 | 11.61798 | -2.84643 | 0.005268 | 0.03662653 | -2.4658487 |
| ZNF572 | -1.02548 | 6.732305 | -2.84614 | 0.005273 | 0.03662653 | -2.4666063 |
| SYNJ2 | -2.78112 | 16.72751 | -2.84602 | 0.005275 | 0.03662653 | -2.4669027 |
| GSC | -1.15065 | 7.980016 | -2.84584 | 0.005278 | 0.03662653 | -2.4673799 |
| DND1 | -5.42213 | 27.74146 | -2.84505 | 0.00529 | 0.03668537 | -2.4694144 |
| HGF | -2.88602 | 13.5318 | -2.8448 | 0.005294 | 0.03668632 | -2.4700575 |
| IDE | -2.58288 | 18.26773 | -2.84423 | 0.005302 | 0.03670839 | -2.4715097 |
| SCN1B | -2.0351 | 14.00301 | -2.8437 | 0.00531 | 0.0367259 | -2.4728649 |
| SNAPC4 | -2.60827 | 18.80964 | -2.84242 | 0.00533 | 0.0368125 | -2.4761755 |
| FXYD2 | -2.19399 | 14.29336 | -2.8395 | 0.005376 | 0.03709209 | -2.4836839 |
| SLC6A3 | -2.22667 | 12.21345 | -2.83935 | 0.005379 | 0.03709209 | -2.4840732 |
| MPG | -1.87794 | 16.41387 | -2.83871 | 0.005388 | 0.03713467 | -2.4856997 |
| SLC22A2 | -1.6402 | 9.972586 | -2.83791 | 0.005401 | 0.03719564 | -2.4877603 |
| GRIA3 | -3.23271 | 7.589117 | -2.8367 | 0.00542 | 0.03727494 | -2.4908661 |
| JUNB | -20.1659 | 50.13178 | -2.83584 | 0.005434 | 0.03731668 | -2.4930803 |
| CACNB3 | -1.88477 | 14.41031 | -2.83514 | 0.005445 | 0.03736493 | -2.4948757 |
| LYPLA2 | -4.97745 | 31.68444 | -2.83283 | 0.005482 | 0.03756706 | -2.5008071 |
| RASA3 | -2.96394 | 20.15883 | -2.83012 | 0.005525 | 0.0378117 | -2.5077447 |
| ABHD8 | -1.08792 | 10.13075 | -2.82969 | 0.005532 | 0.03781492 | -2.508862 |
| C1QTNF4 | -1.12929 | 9.208306 | -2.82962 | 0.005533 | 0.03781492 | -2.509042 |
| DNM2 | -2.59591 | 16.85193 | -2.82868 | 0.005548 | 0.03789173 | -2.5114384 |
| CRYBB1 | -1.25983 | 8.777888 | -2.82759 | 0.005566 | 0.03796621 | -2.5142331 |
| HPS3 | -2.3964 | 13.92386 | -2.82742 | 0.005569 | 0.03796621 | -2.514667 |
| ACAA1 | -4.88085 | 32.82214 | -2.82659 | 0.005582 | 0.03796621 | -2.516789 |
| DUSP15 | -1.01675 | 9.802576 | -2.82638 | 0.005586 | 0.03796621 | -2.5173361 |
| PDGFB | -1.47093 | 11.31997 | -2.82634 | 0.005586 | 0.03796621 | -2.517428 |
| CD1A | -1.77964 | 10.64786 | -2.8245 | 0.005616 | 0.03810928 | -2.5221514 |
| HCK | -7.68184 | 18.47216 | -2.82437 | 0.005618 | 0.03810928 | -2.5224788 |
| DMBT1 | -1.16679 | 8.763628 | -2.82385 | 0.005627 | 0.03810928 | -2.5238021 |
| FIGN | 2.91315 | 9.518837 | 2.823751 | 0.005628 | 0.03810928 | -2.5240561 |
| APBA2 | -6.19844 | 15.39752 | -2.82363 | 0.00563 | 0.03810928 | -2.5243734 |
| MAP1LC3B | -6.56849 | 38.26797 | -2.82188 | 0.005659 | 0.03824804 | -2.5288534 |
| FAM20C | -3.28522 | 22.13991 | -2.82167 | 0.005663 | 0.03824804 | -2.5293825 |
| RTKN | -1.76201 | 11.1855 | -2.82073 | 0.005678 | 0.03832569 | -2.5317702 |
| NUPL2 | -1.9644 | 13.65841 | -2.82047 | 0.005682 | 0.03832608 | -2.5324431 |
| TRIM41 | -1.476 | 15.70549 | -2.81707 | 0.005739 | 0.03860278 | -2.541116 |
| PITX3 | -1.92696 | 11.49157 | -2.81622 | 0.005753 | 0.03864685 | -2.5433066 |
| UBQLN3 | -1.03288 | 8.076065 | -2.81621 | 0.005753 | 0.03864685 | -2.5433202 |
| SLC37A4 | -1.69887 | 13.23458 | -2.81574 | 0.005761 | 0.03867335 | -2.5445225 |
| LIMS1 | -10.0782 | 46.39054 | -2.81475 | 0.005777 | 0.038758 | -2.5470445 |
| MLN | -1.04177 | 7.674659 | -2.812 | 0.005824 | 0.0390153 | -2.55406 |
| SOX7 | 3.408622 | 13.09885 | 2.811621 | 0.00583 | 0.03902841 | -2.5550244 |
| THRA | 2.61937 | 17.80887 | 2.810863 | 0.005843 | 0.039033 | -2.5569553 |
| CSTF3 | -2.44452 | 23.64805 | -2.81068 | 0.005846 | 0.039033 | -2.5574155 |
| CHRNB2 | -2.86438 | 17.94093 | -2.81046 | 0.00585 | 0.039033 | -2.5579882 |
| KCNQ2 | -1.24413 | 7.44159 | -2.81035 | 0.005852 | 0.039033 | -2.558272 |
| APOL1 | -8.84407 | 29.54951 | -2.8102 | 0.005854 | 0.039033 | -2.5586354 |
| HDAC3 | -4.16799 | 36.67127 | -2.80963 | 0.005864 | 0.03907178 | -2.5601034 |
| ZNF575 | -2.19753 | 16.4552 | -2.8092 | 0.005871 | 0.03907597 | -2.5611889 |
| E2F7 | -1.85986 | 7.731791 | -2.80847 | 0.005884 | 0.03912339 | -2.5630464 |
| MRPL54 | -3.29846 | 19.6152 | -2.80608 | 0.005924 | 0.0393166 | -2.5691227 |
| C20orf27 | -1.50422 | 12.49865 | -2.80607 | 0.005925 | 0.0393166 | -2.5691556 |
| STARD8 | -1.85724 | 13.32871 | -2.80574 | 0.00593 | 0.03932723 | -2.5699853 |
| FABP6 | -1.15949 | 7.497243 | -2.8048 | 0.005946 | 0.03938131 | -2.5723759 |
| KIAA1462 | -2.90846 | 16.12321 | -2.80423 | 0.005956 | 0.03942039 | -2.5738384 |
| GPR34 | -7.18137 | 15.45456 | -2.80341 | 0.00597 | 0.03948365 | -2.575912 |
| LAMC3 | -10.2165 | 26.34812 | -2.80321 | 0.005974 | 0.03948365 | -2.5764282 |
| UNC119 | -3.61944 | 25.18924 | -2.7995 | 0.006038 | 0.03985574 | -2.5858491 |
| TBC1D16 | -2.09673 | 14.29555 | -2.79845 | 0.006057 | 0.03995047 | -2.5885234 |
| LST1 | -5.9521 | 22.90294 | -2.79698 | 0.006082 | 0.04009345 | -2.5922504 |
| RIMS3 | 4.168805 | 16.422 | 2.794897 | 0.006119 | 0.04028132 | -2.597532 |
| VPREB1 | -1.70677 | 7.735965 | -2.79333 | 0.006147 | 0.0403846 | -2.6015132 |
| TPO | 1.472583 | 7.314818 | 2.793316 | 0.006147 | 0.0403846 | -2.6015408 |
| GTPBP2 | -1.9749 | 17.76613 | -2.7926 | 0.00616 | 0.04039168 | -2.6033498 |
| CALR | -18.1376 | 114.6433 | -2.79257 | 0.00616 | 0.04039168 | -2.6034421 |
| KIR2DS1 | -1.20551 | 8.012205 | -2.79256 | 0.00616 | 0.04039168 | -2.6034509 |
| CDYL2 | -1.76745 | 12.36213 | -2.79166 | 0.006176 | 0.04046445 | -2.6057244 |
| CHRNG | -1.15446 | 8.588689 | -2.79148 | 0.00618 | 0.04046445 | -2.6062003 |
| TPCN1 | -2.0829 | 18.19941 | -2.79115 | 0.006186 | 0.04047575 | -2.6070293 |
| TLR5 | -3.56721 | 16.69911 | -2.79047 | 0.006198 | 0.04052779 | -2.6087411 |
| ZIC2 | -16.2393 | 36.33819 | -2.78916 | 0.006221 | 0.04065467 | -2.6120688 |
| TAC3 | -1.33391 | 10.73946 | -2.78806 | 0.006241 | 0.04072917 | -2.614841 |
| PI16 | 4.836247 | 20.1378 | 2.787815 | 0.006245 | 0.04073123 | -2.615467 |
| SPTBN5 | -1.11261 | 8.572111 | -2.78689 | 0.006262 | 0.04081337 | -2.6178171 |
| MYBPC2 | -1.17719 | 7.309239 | -2.78645 | 0.00627 | 0.04083794 | -2.6189263 |
| KLK5 | -2.74331 | 13.00697 | -2.78195 | 0.006352 | 0.04130993 | -2.6302836 |
| SALL4 | -1.68737 | 9.227146 | -2.78177 | 0.006355 | 0.04130993 | -2.6307542 |
| APOL2 | -6.29804 | 34.12157 | -2.78116 | 0.006366 | 0.04135528 | -2.6322963 |
| PPP2R2B | 4.278326 | 11.84466 | 2.780419 | 0.00638 | 0.04136856 | -2.634155 |
| GALNT6 | -2.53144 | 8.604793 | -2.7804 | 0.00638 | 0.04136856 | -2.6342092 |
| SLC35A4 | -2.82395 | 24.88247 | -2.78027 | 0.006383 | 0.04136856 | -2.6345398 |
| ITGB7 | -3.10434 | 16.09787 | -2.78013 | 0.006385 | 0.04136856 | -2.6348908 |
| HAP1 | -1.19159 | 9.240716 | -2.77967 | 0.006393 | 0.04139551 | -2.6360395 |
| SLC39A13 | -2.3598 | 18.99347 | -2.77852 | 0.006415 | 0.04150502 | -2.638935 |
| CYP26B1 | -2.63321 | 10.33159 | -2.77765 | 0.006431 | 0.04158259 | -2.6411498 |
| CSF2 | -1.04324 | 7.617175 | -2.77688 | 0.006445 | 0.04162829 | -2.6430801 |
| SFMBT1 | -1.21313 | 9.598833 | -2.77681 | 0.006446 | 0.04162829 | -2.6432652 |
| XPNPEP1 | -6.57242 | 49.85539 | -2.77077 | 0.006559 | 0.04224538 | -2.6584732 |
| MTA1 | -3.2835 | 20.15671 | -2.76606 | 0.006648 | 0.0427511 | -2.670324 |
| DLG2 | 2.202898 | 8.372958 | 2.765935 | 0.006651 | 0.0427511 | -2.670629 |
| LMNB2 | -2.84174 | 14.89359 | -2.76549 | 0.006659 | 0.04277811 | -2.6717551 |
| CHRD | -1.19257 | 10.44055 | -2.76477 | 0.006673 | 0.04281078 | -2.6735678 |
| BCL2L12 | -1.36649 | 13.09473 | -2.76434 | 0.006681 | 0.0428349 | -2.6746327 |
| CCL13 | -1.08876 | 8.529491 | -2.75942 | 0.006776 | 0.04324483 | -2.6869692 |
| IL2RB | -3.75024 | 14.1354 | -2.75823 | 0.006799 | 0.04336484 | -2.6899662 |
| FOXM1 | -1.19654 | 7.192356 | -2.75797 | 0.006804 | 0.04336903 | -2.6906186 |
| DDHD1 | -1.73707 | 13.05889 | -2.75675 | 0.006828 | 0.04346362 | -2.6936619 |
| CTSW | -1.07442 | 8.138559 | -2.75556 | 0.006851 | 0.04358381 | -2.6966486 |
| SMUG1 | -2.58728 | 20.94384 | -2.7545 | 0.006872 | 0.04360817 | -2.6993028 |
| CCK | -1.33729 | 8.354781 | -2.75412 | 0.006879 | 0.04360817 | -2.7002559 |
| PDE7A | -1.85609 | 9.497569 | -2.75411 | 0.006879 | 0.04360817 | -2.7002865 |
| UBE2J2 | -1.61329 | 14.99449 | -2.75406 | 0.00688 | 0.04360817 | -2.7003973 |
| PKMYT1 | -1.94417 | 12.06848 | -2.75401 | 0.006881 | 0.04360817 | -2.7005282 |
| PTPRE | -5.51903 | 15.57392 | -2.75319 | 0.006897 | 0.04367737 | -2.7025784 |
| CDT1 | -4.04361 | 20.54469 | -2.75291 | 0.006903 | 0.04367737 | -2.7032827 |
| AK1 | -2.44457 | 20.91753 | -2.75268 | 0.006908 | 0.04367737 | -2.7038699 |
| ECHDC3 | -1.22277 | 11.49164 | -2.75244 | 0.006912 | 0.04367737 | -2.7044659 |
| CKAP2 | -1.31199 | 8.007844 | -2.75233 | 0.006914 | 0.04367737 | -2.7047325 |
| CMKLR1 | -6.77938 | 26.38936 | -2.75165 | 0.006928 | 0.04370711 | -2.7064521 |
| PTPN9 | -1.4923 | 13.61132 | -2.74954 | 0.00697 | 0.04394201 | -2.7117092 |
| IPO4 | -2.54841 | 15.15855 | -2.74931 | 0.006974 | 0.04394271 | -2.7122839 |
| SPAG5 | -1.56221 | 11.13219 | -2.74829 | 0.006995 | 0.04404325 | -2.7148463 |
| PITX1 | -1.38634 | 8.478073 | -2.74726 | 0.007015 | 0.0441444 | -2.7174156 |
| AVEN | -5.12565 | 25.23028 | -2.74506 | 0.007059 | 0.04436948 | -2.7229117 |
| FBXW2 | -2.56374 | 23.0566 | -2.74424 | 0.007076 | 0.04442766 | -2.7249523 |
| RFX5 | -2.88095 | 24.2497 | -2.74412 | 0.007078 | 0.04442766 | -2.7252536 |
| FOXD1 | -7.51254 | 33.33843 | -2.74344 | 0.007092 | 0.04448527 | -2.7269466 |
| SCGN | -1.30537 | 7.092223 | -2.74311 | 0.007099 | 0.04449868 | -2.727768 |
| TPP2 | -3.96928 | 31.69595 | -2.74285 | 0.007104 | 0.04450364 | -2.7284229 |
| AKAP5 | -2.01564 | 7.541795 | -2.74148 | 0.007132 | 0.04464868 | -2.7318289 |
| ANKRD1 | -6.69831 | 12.56924 | -2.74069 | 0.007148 | 0.04472136 | -2.7338096 |
| KIAA1755 | -9.19681 | 27.38605 | -2.73962 | 0.007169 | 0.04479187 | -2.736462 |
| CHCHD6 | -1.33798 | 12.20122 | -2.73916 | 0.007179 | 0.04479187 | -2.7376267 |
| STMN1 | -3.76183 | 21.11339 | -2.73904 | 0.007181 | 0.04479187 | -2.7379255 |
| INSL3 | -1.0271 | 8.16346 | -2.73795 | 0.007204 | 0.04490013 | -2.7406424 |
| PPP2R1A | -5.92688 | 45.19805 | -2.73773 | 0.007208 | 0.04490013 | -2.7411876 |
| MAPK8 | -1.45389 | 14.48873 | -2.73736 | 0.007216 | 0.04491879 | -2.7421049 |
| CDCA5 | -1.7916 | 10.82637 | -2.73325 | 0.007301 | 0.04537199 | -2.7523379 |
| ALAS2 | 5.441485 | 11.03663 | 2.733161 | 0.007302 | 0.04537199 | -2.7525504 |
| SLC1A7 | -1.31098 | 9.363853 | -2.73198 | 0.007327 | 0.04549527 | -2.7554769 |
| CBX8 | -1.93617 | 12.41891 | -2.73035 | 0.007361 | 0.04564405 | -2.7595317 |
| FJX1 | -2.23865 | 11.74147 | -2.73017 | 0.007365 | 0.04564405 | -2.7599878 |
| BCAR1 | -2.04931 | 14.77566 | -2.72889 | 0.007391 | 0.04572574 | -2.7631548 |
| GFER | -1.55386 | 12.15121 | -2.72867 | 0.007396 | 0.04572574 | -2.7637029 |
| DGKG | 1.926405 | 10.15518 | 2.728653 | 0.007397 | 0.04572574 | -2.7637524 |
| PTGES | -1.57505 | 10.86512 | -2.72773 | 0.007416 | 0.04578615 | -2.7660522 |
| KCNB1 | 1.987171 | 10.21889 | 2.727145 | 0.007428 | 0.04578615 | -2.7674966 |
| IFNB1 | -1.11912 | 5.83083 | -2.72708 | 0.00743 | 0.04578615 | -2.7676493 |
| TERF2 | -2.93532 | 25.0158 | -2.72665 | 0.007439 | 0.04581411 | -2.7687307 |
| PPARD | -5.13267 | 23.97422 | -2.72468 | 0.00748 | 0.04601239 | -2.7736021 |
| APEX2 | -1.41259 | 13.89146 | -2.72428 | 0.007489 | 0.04603674 | -2.7746111 |
| TCF19 | -1.11957 | 10.40435 | -2.72157 | 0.007547 | 0.0463624 | -2.7813223 |
| RASD2 | -1.23115 | 10.38494 | -2.72116 | 0.007555 | 0.0463876 | -2.7823431 |
| SOX5 | 3.2357 | 10.52153 | 2.720546 | 0.007568 | 0.04643896 | -2.7838559 |
| ERCC1 | -5.73473 | 32.01529 | -2.71904 | 0.007601 | 0.04660886 | -2.7875941 |
| IGSF9 | -1.669 | 14.96392 | -2.71787 | 0.007626 | 0.04670477 | -2.7904798 |
| ARPC4 | -7.23777 | 55.44889 | -2.71609 | 0.007665 | 0.04688294 | -2.7948957 |
| FUT11 | -3.58344 | 20.31748 | -2.71475 | 0.007694 | 0.0470317 | -2.7982084 |
| NR5A1 | -1.17614 | 10.35701 | -2.71259 | 0.007741 | 0.04723104 | -2.8035318 |
| PEX16 | -2.72377 | 27.29327 | -2.71207 | 0.007752 | 0.04725501 | -2.804822 |
| OSBPL10 | -2.05075 | 15.62962 | -2.71172 | 0.00776 | 0.04725501 | -2.8056893 |
| PKLR | -1.01833 | 7.916644 | -2.71137 | 0.007767 | 0.04725501 | -2.8065428 |
| NLGN2 | -1.64207 | 13.6561 | -2.71135 | 0.007768 | 0.04725501 | -2.8065867 |
| ERBB4 | 2.797981 | 9.386105 | 2.710756 | 0.007781 | 0.04730081 | -2.8080653 |
| SPRR4 | -2.36607 | 17.03846 | -2.70977 | 0.007803 | 0.04737429 | -2.8105004 |
| CXCR6 | -1.09076 | 7.52558 | -2.70752 | 0.007852 | 0.04764671 | -2.8160497 |
| NSD1 | -1.00421 | 12.0556 | -2.70455 | 0.007918 | 0.04798127 | -2.8233634 |
| SNPH | -2.30246 | 12.59702 | -2.70445 | 0.007921 | 0.04798127 | -2.8236266 |
| TM9SF1 | -2.95041 | 20.94474 | -2.70439 | 0.007922 | 0.04798127 | -2.823777 |
| SHD | -1.03145 | 9.75497 | -2.70304 | 0.007952 | 0.0481346 | -2.8271015 |
| APLN | -1.96746 | 10.8357 | -2.70165 | 0.007984 | 0.04829392 | -2.8305248 |
| PEX26 | -1.39189 | 13.12462 | -2.69927 | 0.008037 | 0.04858872 | -2.8363768 |
| FAM20B | 6.477628 | 42.61502 | 2.698749 | 0.008049 | 0.04862988 | -2.8376522 |
| CXCR3 | -1.24794 | 8.794993 | -2.69717 | 0.008085 | 0.0487861 | -2.841524 |
| WISP2 | 17.66354 | 35.22232 | 2.69681 | 0.008093 | 0.04880619 | -2.8424186 |
| DHRS9 | -3.55409 | 9.817627 | -2.69659 | 0.008098 | 0.0488069 | -2.8429658 |
| NOTCH3 | 10.67163 | 41.22904 | 2.695986 | 0.008112 | 0.04885961 | -2.8444425 |
| LRRTM4 | 1.474891 | 7.167196 | 2.695498 | 0.008123 | 0.04889691 | -2.8456428 |
| ULBP2 | -1.17185 | 8.16257 | -2.69511 | 0.008132 | 0.04891525 | -2.8465863 |
| LMAN1 | -10.2821 | 60.2159 | -2.69493 | 0.008136 | 0.04891525 | -2.8470371 |
| ZNF23 | 2.937236 | 15.28104 | 2.69415 | 0.008154 | 0.04895623 | -2.8489523 |
| IL4I1 | -3.22815 | 13.56856 | -2.69398 | 0.008158 | 0.04895623 | -2.8493658 |
| NEGR1 | 2.8073 | 12.02662 | 2.693199 | 0.008176 | 0.04899892 | -2.851288 |
| CAPN10 | -1.42555 | 9.777543 | -2.69282 | 0.008184 | 0.04899892 | -2.8522123 |
| ADAT1 | -1.6263 | 14.68509 | -2.69045 | 0.008239 | 0.04926487 | -2.8580384 |
| GRB7 | -1.63543 | 11.3678 | -2.68737 | 0.008311 | 0.0496328 | -2.865592 |
| BCCIP | 2.478114 | 20.0123 | 2.68698 | 0.00832 | 0.04964838 | -2.8665414 |
| VDR | -2.59096 | 10.81268 | -2.68682 | 0.008323 | 0.04964838 | -2.8669238 |
| ZC3HAV1 | -6.64406 | 39.35727 | -2.68623 | 0.008337 | 0.04965688 | -2.8683794 |
| NDFIP2 | -1.74155 | 12.81601 | -2.68612 | 0.00834 | 0.04965688 | -2.8686575 |
| SLC16A2 | -1.37948 | 13.12075 | -2.68482 | 0.00837 | 0.04979685 | -2.871829 |
| FBXL8 | -1.09458 | 8.496269 | -2.68441 | 0.00838 | 0.04979685 | -2.8728351 |
| NPPC | -1.32028 | 10.68523 | -2.68354 | 0.008401 | 0.04981095 | -2.8749649 |
| TNFRSF10B | -6.59145 | 35.531 | -2.68213 | 0.008434 | 0.04997317 | -2.8784077 |
