# Supplementary material for: Genetic polymorphisms and transcription profiles associated with intracranial aneurysm: a key role for NOTCH3
Source: Aging (Albany NY). 2019 Jul 23;11(14):5173–91. doi: 10.18632/aging.102111 (PMC6682524; doi:10.18632/aging.102111)
Supplement: Supplementary Table 3 [file aging-11-102111-s001.docx]

**Supplementary Table 3. IA sample and cerebral artery sample of GEO.**

| **Sample ID** | **GSM_ID** | **Acession** | **Sample type(human)** | **Platform** | **Group** |
| --- | --- | --- | --- | --- | --- |
| 1 | GSM151655 | GSE6551 | intracranial aneurysm | GPL570 | IA Case |
| 2 | GSM151656 | GSE6551 | intracrania aneurysm | GPL570 | IA Case |
| 3 | GSM151657 | GSE6551 | intracranial artery | GPL2507 | Control |
| 4 | GSM151658 | GSE6551 | intracranial artery | GPL2507 | Control |
| 5 | GSM151659 | GSE6551 | intracranial artery | GPL2507 | Control |
| 6 | GSM151660 | GSE6551 | intracranial artery | GPL2507 | Control |
| 7 | GSM151661 | GSE6551 | intracranial artery | GPL2507 | Control |
| 8 | GSM151662 | GSE6551 | intracranial aneurysm | GPL570 | IA Case |
| 9 | GSM151663 | GSE6551 | intracranial aneurysm | GPL570 | IA Case |
| 10 | GSM151664 | GSE6551 | intracranial aneurysm | GPL570 | IA Case |
| 11 | GSM391277 | GSE15629 | Ruptured intracranial aneurysm | GPL6244 | IA Case |
| 12 | GSM391278 | GSE15629 | Unruptured intracranial aneurysm | GPL6244 | IA Case |
| 13 | GSM391279 | GSE15629 | Control, Rep1 | GPL6244 | Control |
| 14 | GSM391280 | GSE15629 | Control, Rep2 | GPL6244 | Control |
| 15 | GSM391281 | GSE15629 | Unruptured intracranial aneurysm | GPL6244 | IA Case |
| 16 | GSM391282 | GSE15629 | Control, Rep3 | GPL6244 | Control |
| 17 | GSM391283 | GSE15629 | Ruptured intracranial aneurysm | GPL6244 | IA Case |
| 18 | GSM391284 | GSE15629 | Unruptured intracranial aneurysm | GPL6244 | IA Case |
| 19 | GSM391285 | GSE15629 | Unruptured intracranial aneurysm | GPL6244 | IA Case |
| 20 | GSM391286 | GSE15629 | Control, Rep4 | GPL6244 | Control |
| 21 | GSM391287 | GSE15629 | Ruptured intracranial aneurysm | GPL6244 | IA Case |
| 22 | GSM391288 | GSE15629 | Unruptured intracranial aneurysm | GPL6244 | IA Case |
| 23 | GSM391289 | GSE15629 | Ruptured intracranial aneurysm | GPL6244 | IA Case |
| 24 | GSM391290 | GSE15629 | Ruptured intracranial aneurysm | GPL6244 | IA Case |
| 25 | GSM391291 | GSE15629 | Ruptured intracranial aneurysm | GPL6244 | IA Case |
| 26 | GSM391292 | GSE15629 | Unruptured intracranial aneurysm | GPL6244 | IA Case |
| 27 | GSM391293 | GSE15629 | Ruptured intracranial aneurysm | GPL6244 | IA Case |
| 28 | GSM391294 | GSE15629 | Ruptured intracranial aneurysm | GPL6244 | IA Case |
| 29 | GSM391295 | GSE15629 | Control, Rep5 | GPL6244 | Control |
| 30 | GSM337102 | GSE13353 | Ruptured intracranial aneurysm | GPL570 | IA Case |
| 31 | GSM337103 | GSE13353 | Ruptured_rep2 | GPL570 | IA Case |
| 32 | GSM337104 | GSE13353 | Ruptured_rep3 | GPL570 | IA Case |
| 33 | GSM337105 | GSE13353 | aneurysm_ruptured_rep4 | GPL570 | IA Case |
| 34 | GSM337106 | GSE13353 | aneurysm_ruptured_rep5 | GPL570 | IA Case |
| 35 | GSM337107 | GSE13353 | aneurysm_unruptured_rep1 | GPL570 | IA Case |
| 36 | GSM337108 | GSE13353 | aneurysm_unruptured_rep2 | GPL570 | IA Case |
| 37 | GSM337109 | GSE13353 | aneurysm_ruptured_rep6 | GPL570 | IA Case |
| 38 | GSM337110 | GSE13353 | aneurysm_unruptured_rep3 | GPL570 | IA Case |
| 39 | GSM337111 | GSE13353 | aneurysm_unruptured_rep4 | GPL570 | IA Case |
| 40 | GSM337112 | GSE13353 | aneurysm_unruptured_rep5 | GPL570 | IA Case |
| 41 | GSM337113 | GSE13353 | aneurysm_unruptured_rep6 | GPL570 | IA Case |
| 42 | GSM337114 | GSE13353 | aneurysm_unruptured_rep7 | GPL570 | IA Case |
| 43 | GSM337115 | GSE13353 | aneurysm_unruptured_rep8 | GPL570 | IA Case |
| 44 | GSM337116 | GSE13353 | aneurysm_ruptured_rep7 | GPL570 | IA Case |
| 45 | GSM337117 | GSE13353 | aneurysm_ruptured_rep8 | GPL570 | IA Case |
| 46 | GSM337118 | GSE13353 | aneurysm_ruptured_rep9 | GPL570 | IA Case |
| 47 | GSM337119 | GSE13353 | aneurysm_ruptured_rep10 | GPL570 | IA Case |
| 48 | GSM337120 | GSE13353 | aneurysm_ruptured_rep11 | GPL570 | IA Case |
| 49 | GSM663769 | GSE26969 | normal arterial tissue: sample #1 | GPL570 | Control |
| 50 | GSM663770 | GSE26969 | normal arterial tissue: sample #2 | GPL570 | Control |
| 51 | GSM663771 | GSE26969 | normal arterial tissue: sample #3 | GPL570 | Control |
| 52 | GSM663772 | GSE26969 | aneurysmal tissue: sample #1 | GPL570 | IA Case |
| 53 | GSM663773 | GSE26969 | aneurysmal tissue: sample #2 | GPL570 | IA Case |
| 54 | GSM663774 | GSE26969 | aneurysmal tissue: sample #3 | GPL570 | IA Case |
| 55 | GSM1128759 | GSE46337 | IA_rep1 [mRNA] | GPL6480 | IA Case |
| 56 | GSM1128760 | GSE46337 | IA_rep2 [mRNA] | GPL6480 | IA Case |
| 57 | GSM1128761 | GSE46337 | MMA_rep1 [mRNA] | GPL6480 | Control |
| 58 | GSM1128762 | GSE46337 | MMA_rep2 [mRNA] | GPL6480 | Control |
| 59 | GSM1306885 | GSE54083 | Ruptured intracranial aneurysm, sample 1 | GPL4133 | IA Case |
| 60 | GSM1306886 | GSE54083 | Ruptured intracranial aneurysm, sample 2 | GPL4133 | IA Case |
| 61 | GSM1306887 | GSE54083 | Ruptured intracranial aneurysm, sample 3 | GPL4133 | IA Case |
| 62 | GSM1306888 | GSE54083 | Ruptured intracranial aneurysm, sample 4 | GPL4133 | IA Case |
| 63 | GSM1306889 | GSE54083 | Ruptured intracranial aneurysm, sample 5 | GPL4133 | IA Case |
| 64 | GSM1306890 | GSE54083 | Ruptured intracranial aneurysm, sample 6 | GPL4133 | IA Case |
| 65 | GSM1306891 | GSE54083 | Ruptured intracranial aneurysm, sample 7 | GPL4133 | IA Case |
| 66 | GSM1306892 | GSE54083 | Ruptured intracranial aneurysm, sample 8 | GPL4133 | IA Case |
| 67 | GSM1306893 | GSE54083 | Unruptured intracranial aneurysm, sample 1 | GPL4133 | IA Case |
| 68 | GSM1306894 | GSE54083 | Unruptured intracranial aneurysm, sample 2 | GPL4133 | IA Case |
| 69 | GSM1306895 | GSE54083 | Unruptured intracranial aneurysm, sample 3 | GPL4133 | IA Case |
| 70 | GSM1306896 | GSE54083 | Unruptured intracranial aneurysm, sample 4 | GPL4133 | IA Case |
| 71 | GSM1306897 | GSE54083 | Unruptured intracranial aneurysm, sample 5 | GPL4133 | IA Case |
| 72 | GSM1306898 | GSE54083 | Superficial temporal artery, sample 1 | GPL4133 | Control |
| 73 | GSM1306899 | GSE54083 | Superficial temporal artery, sample 2 | GPL4133 | Control |
| 74 | GSM1306900 | GSE54083 | Superficial temporal artery, sample 3 | GPL4133 | Control |
| 75 | GSM1306901 | GSE54083 | Superficial temporal artery, sample 4 | GPL4133 | Control |
| 76 | GSM1306902 | GSE54083 | Superficial temporal artery, sample 5 | GPL4133 | Control |
| 77 | GSM1306903 | GSE54083 | Superficial temporal artery, sample 6 | GPL4133 | Control |
| 78 | GSM1306904 | GSE54083 | Superficial temporal artery, sample 7 | GPL4133 | Control |
| 79 | GSM1306905 | GSE54083 | Superficial temporal artery, sample 8 | GPL4133 | Control |
| 80 | GSM1306906 | GSE54083 | Superficial temporal artery, sample 9 | GPL4133 | Control |
| 81 | GSM1306907 | GSE54083 | Superficial temporal artery, sample 10 | GPL4133 | Control |
| 82 | GSM1619668 | GSE66238 | hsa_BrainAneurysmKB20_RNASeq | GPL17303 | IA Case |
| 83 | GSM1619669 | GSE66238 | hsa_BrainAneurysmKB3_RNASeq | GPL17303 | IA Case |
| 84 | GSM1619670 | GSE66238 | hsa_BrainAneurysmKB5_RNASeq | GPL17303 | IA Case |
| 85 | GSM1619671 | GSE66238 | hsa_BrainAneurysmKB6_RNASeq | GPL17303 | IA Case |
| 86 | GSM1619672 | GSE66238 | hsa_BrainAneurysmKB7_RNASeq | GPL17303 | IA Case |
| 87 | GSM1619673 | GSE66238 | hsa_BrainAneurysmKB8_RNASeq | GPL17303 | IA Case |
| 88 | GSM1619674 | GSE66238 | hsa_ControlKBC10_RNASeq | GPL17303 | Control |
| 89 | GSM1619675 | GSE66238 | hsa_ControlKBC11_RNASeq | GPL17303 | Control |
| 90 | GSM1619676 | GSE66238 | hsa_ControlKBC12_RNASeq | GPL17303 | Control |
| 91 | GSM1619677 | GSE66238 | hsa_ControlKBC13_RNASeq | GPL17303 | Control |
| 92 | GSM1619678 | GSE66238 | hsa_ControlKBC14_R1_RNASeq | GPL17303 | Control |
| 93 | GSM1619679 | GSE66238 | hsa_ControlKBC14_R2_RNASeq | GPL17303 | Control |
| 94 | GSM1619680 | GSE66238 | hsa_ControlKBC15_RNASeq | GPL17303 | Control |
| 95 | GSM1619681 | GSE66238 | hsa_ControlKBC16_RNASeq | GPL17303 | Control |
| 96 | GSM1619682 | GSE66238 | hsa_ControlKBC3_RNASeq | GPL17303 | Control |
| 97 | GSM1619683 | GSE66238 | hsa_ControlKBC7_R1_RNASeq | GPL17303 | Control |
| 98 | GSM1619684 | GSE66238 | hsa_ControlKBC7_R2_RNASeq | GPL17303 | Control |
| 99 | GSM1619685 | GSE66238 | hsa_ControlKBC8_RNASeq | GPL17303 | Control |
| 100 | GSM1955147 | GSE75436 | intracranial aneurysm1 matched superficial temporal artery | GPL570 | Control |
| 101 | GSM1955148 | GSE75436 | intracranial aneurysm1 | GPL570 | IA Case |
| 102 | GSM1955149 | GSE75436 | intracranial aneurysm2 matched superficial temporal artery | GPL570 | Control |
| 103 | GSM1955150 | GSE75436 | intracranial aneurysm2 | GPL570 | IA Case |
| 104 | GSM1955151 | GSE75436 | intracranial aneurysm3 matched superficial temporal artery | GPL570 | Control |
| 105 | GSM1955152 | GSE75436 | intracranial aneurysm3 | GPL570 | IA Case |
| 106 | GSM1955153 | GSE75436 | intracranial aneurysm4 | GPL570 | IA Case |
| 107 | GSM1955154 | GSE75436 | intracranial aneurysm4 matched superficial temporal artery | GPL570 | Control |
| 108 | GSM1955155 | GSE75436 | intracranial aneurysm5 | GPL570 | IA Case |
| 109 | GSM1955156 | GSE75436 | intracranial aneurysm5 matched superficial temporal artery | GPL570 | Control |
| 110 | GSM1955157 | GSE75436 | intracranial aneurysm6 | GPL570 | IA Case |
| 111 | GSM1955158 | GSE75436 | intracranial aneurysm6 matched superficial temporal artery | GPL570 | Control |
| 112 | GSM1955159 | GSE75436 | intracranial aneurysm7 | GPL570 | IA Case |
| 113 | GSM1955160 | GSE75436 | intracranial aneurysm7 matched superficial temporal artery | GPL570 | Control |
| 114 | GSM1955161 | GSE75436 | intracranial aneurysm8 | GPL570 | IA Case |
| 115 | GSM1955162 | GSE75436 | intracranial aneurysm8 matched superficial temporal artery | GPL570 | Control |
| 116 | GSM1955163 | GSE75436 | intracranial aneurysm9 | GPL570 | IA Case |
| 117 | GSM1955164 | GSE75436 | intracranial aneurysm9 matched superficial temporal artery | GPL570 | Control |
| 118 | GSM1955165 | GSE75436 | intracranial aneurysm10 | GPL570 | IA Case |
| 119 | GSM1955166 | GSE75436 | intracranial aneurysm10 matched superficial temporal artery | GPL570 | Control |
| 120 | GSM1955167 | GSE75436 | intracranial aneurysm11 | GPL570 | IA Case |
| 121 | GSM1955168 | GSE75436 | intracranial aneurysm11 matched superficial temporal artery | GPL570 | Control |
| 122 | GSM1955169 | GSE75436 | intracranial aneurysm12 | GPL570 | IA Case |
| 123 | GSM1955170 | GSE75436 | intracranial aneurysm12 matched superficial temporal artery | GPL570 | Control |
| 124 | GSM1955171 | GSE75436 | intracranial aneurysm13 | GPL570 | IA Case |
| 125 | GSM1955172 | GSE75436 | intracranial aneurysm13 matched superficial temporal artery | GPL570 | Control |
| 126 | GSM1955173 | GSE75436 | intracranial aneurysm14 | GPL570 | IA Case |
| 127 | GSM1955174 | GSE75436 | intracranial aneurysm14 matched superficial temporal artery | GPL570 | Control |
| 128 | GSM1955175 | GSE75436 | intracranial aneurysm15 | GPL570 | IA Case |
| 129 | GSM1955176 | GSE75436 | intracranial aneurysm15 matched superficial temporal artery | GPL570 | Control |
